# Hotspot analyses indicate significant conservation gaps for evergreen broadleaved woody plants in China

**DOI:** 10.1038/s41598-017-02098-0

**Published:** 2017-05-12

**Authors:** Yue Xu, Zehao Shen, Lingxiao Ying, Zhiheng Wang, Jihong Huang, Runguo Zang, Youxu Jiang

**Affiliations:** 1 Department of Ecology, MOE Key Laboratory on Earth Surface Processes, College of Urban and Environmental Science, Peking University, Beijing, 100871 China; 2 Institute of Forest Ecology, Environment and Protection, Chinese Academy of Forestry, Beijing, 100093 China; 3 Co-Innovation Center for Sustainable Forestry in Southern China, Nanjing Forestry University, Nanjing, 210037 China

## Abstract

Evergreen broadleaved woody plants (EBWPs) are dominant components in forests and savanna of the global tropic and subtropic regions. Southern China possesses the largest continuous area of subtropical EBWPs distribution, harboring a high proportion of endemic species. Hotspot and gap analyses are effective methods for analyzing the spatial pattern of biodiversity and conservation and were used here for EBWPs in China. Based on a distribution data set of 6,265 EBWPs with a spatial resolution of 50 × 50 km, we measured diversity of EBWPs in China using four indices: species richness, corrected weighted endemism, relative phylogenetic diversity, and phylogenetic endemism. According to the results based on 10% threshold, 15.73% of China’s land area was identified as hotspots using at least one diversity index. Only 2.14% of China’s land area was identified as hotspots for EBWPs by all four metrics simultaneously. Most of the hotspots locate in southern mountains. Moreover, we found substantial conservation gaps for Chinese EBWPs. Only 25.43% of the hotspots are covered by existing nature reserves by more than 10% of their area. We suggest to promote the establishment and management of nature reserve system within the hotspot gaps.

## Introduction

Evergreen broadleaved woody plants (EBWPs) widely distribute over the world, and dominate in major types of forests, shrubs and savanna in tropical and subtropical regions^1^, supporting the persistence of other biodiversity components in great quantity. Most of the 35 global hotspots are distributed in tropical and subtropical regions dominated by EBWPs^2^, indicating the critical role of EBWPs in biodiversity conservation.

Along with the climate warming in the last decades, the number and frequency of evergreen broadleaved species have been repeatedly observed to increase in the temperate forests in Europe, indicating that the detected climatic change might favors EBWPs^3–5^. Therefore, EBWPs can be used as indicators for climate change^6^. On the other hand, intensified human activities in the past century has led to a prominent loss of tropic and subtropic forests. As a major hotspot in tropic, deforestation of the Amazon was at an alarming pace of 1.95 × 10^4^ km^2^/year from 1996 to 2005^7^. In China, most of the subtropical evergreen broadleaved primary forests have degraded to secondary forests and shrubs^8^.

Due to aggravated global climate change and habitat loss caused by human activities, the rate of species extinctions has been increasing more and more rapidly. Thus, improving the contemporary conservation strategies is crucial for minimizing loss of biodiversity^9^. Facing the limited time, funds, and human effort allocated to species conservation, a critical challenge is to prioritize the taxa and regions forconservation^10^. Hotspot analysis and gap analysis, which synthesize species diversity, endemism, and the distribution characteristics in habitats, are the two most commonly used methods for setting priorities^11, 12^.

Hotspot analyses are usually conducted at global scales^11, 13, 14^. Currently, the 35 biodiversity hotspots qualified by Conservation International cover 15.9% of the Earth’s land surface, contain 77% of all endemic plant species, 43% of vertebrates, and 80% of all threatened amphibians^2, 15^. However, diversity maps with finer grain size are needed for practical conservation implementation of conservation at local or regional scales^16^. In China, studies on the priority areas or hotspots for plant diversity have gradually increased, although limited by data source, early results were more based on higher taxonomic units (e.g. genus or family)^17–19^, or restricted to endemic or threatened species^20–22^. It is revealed that hotspot patterns were similar but not identical at different taxonomic levels^19^. Higher taxa (family or genus) or threatened plants alone may not be appropriate for identifying hotspots to facilitate biodiversity conservation at the regional or national level. Furthermore, a growing numbers of diversity indices are used in ecological studies, but it is not clear whether these different indices agree on the pattern of biodiversity hotspots^16^.

China is the home to more than 33,000 vascular plant species^23^, 52.1% of which are endemic species^24^ mainly occurring in southern China^25^. China is also the world’s largest continuous area of EBWPs distribution^26^ harboring a high proportion of narrow-range endemic species^27, 28^, and four of the 35 global biodiversity hotspots are located wholly or partly in the tropical and subtropical regions of China^2^. However, due to its high density of human population, China is also one of countries with the most threatened species in the world^29^. Near-threatened and threatened plants are mainly concentrated in southwestern China where the level of anthropogenic activities was relatively low in the history but has prominently intensified in recent decades^30^. With increasing reorganization of the importance of biodiversity conservation, China has set up a hierarchical system of natural reserves in the past decades, which is constituted by 2,640 nature reserves covering 14.9% of its total land area^31^. These reserves contained 85% of wild animal species and 80.7% of the natural vegetation types in China^32, 33^. Nevertheless, the coverage of nature reserves is fairly low in southern and eastern China^34, 35^, and few previous study has comprehensively investigated the distribution of diversity and performed conservation gap analyses with regard to the existing system of nature reserves^22, 35^. Thus, biodiversity hotspot assessments and gap analyses are urgently required to enhance the efficiency of the nature reserve network in China, and to provide valuable insights that might improve its management, especially for the conservation of EBWPs.

In this study, we analyzed the diversity patterns of EBWPs in China at the national level, and compared with the spatial pattern of existing system of natural reserves. We aimed to achieve the following goals: (1) identifying the characteristics of spatial distribution of SR and endemism for EBWPs in China; (2) identifying the biodiversity hotspots for EBWPs in China based on multiple diversity indices; and (3) assessing conservation gaps in the current nature reserve system of EBWPs hotspots. In order to answer above questions, we identified the hotspots of EBWPs and detected conservation gaps for hotspots in Chinese EBWPs. These results would provide a solid support for promoting conservation of EBWPs globally.

## Results

### Distribution patterns of EBWPs diversity

**Figure 1:**
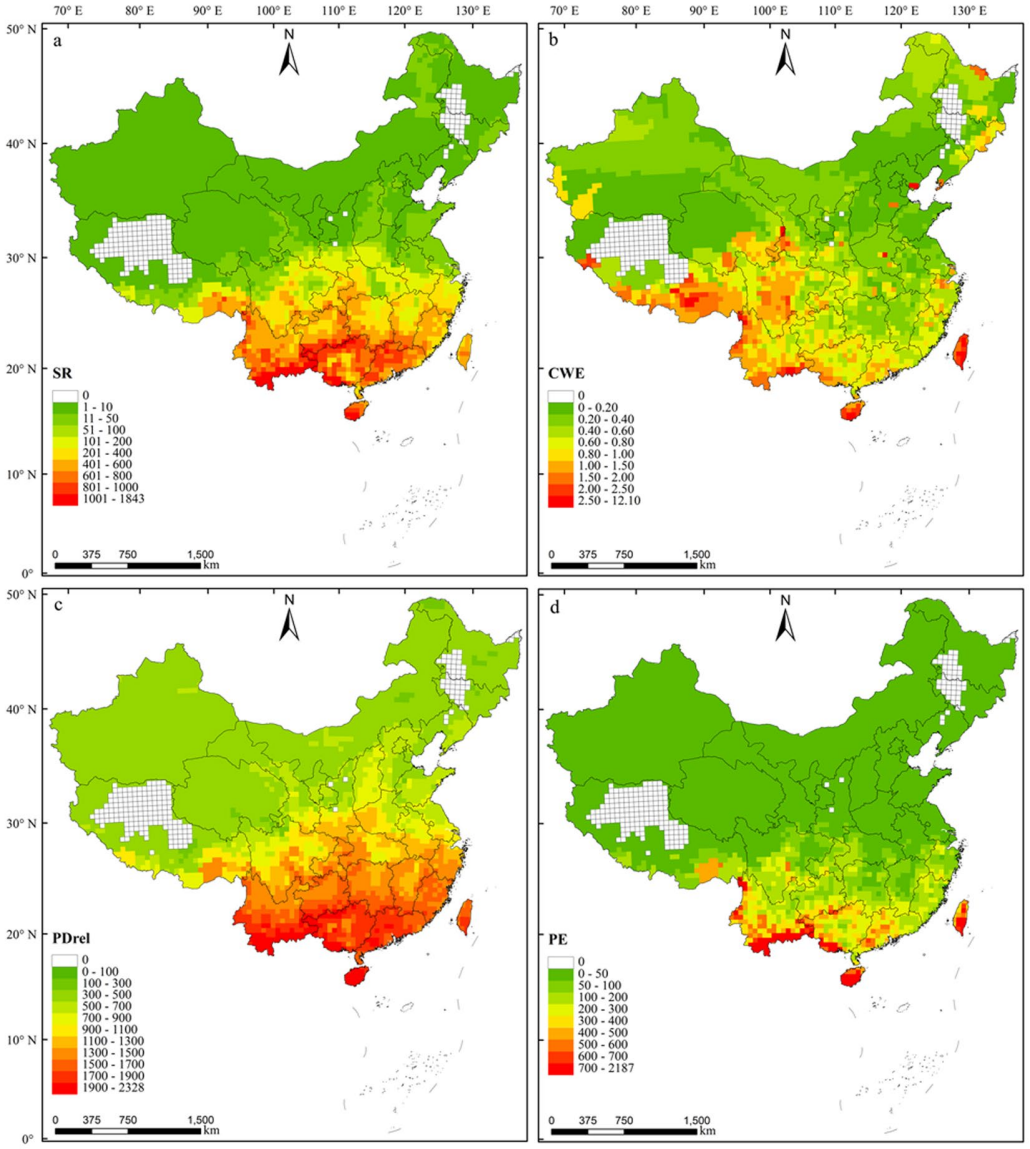
Distribution patterns for (**a**) SR, (**b**) CWE, (**c**) PD_rel_, and (**d**) PE for EBWPs in China. Albers equal-area conic projection. The map was generated using ArcGIS 10.1 (ESRI, Redlands, CA, USA; http://www.esri.com).

The SR pattern of Chinese EBWPs appeared to differ significantly between the south and north, where there was a decreasing trend in SR as the latitude increased. The regions with high SR were mainly distributed in mountainous areas, such as Hengduan Mountains, the mountainous area in south of Yunnan Province, Miaoling Mountains, Nanling Mountains, and Wuzhi Mountains (Fig. 1a). By contrast, the latitude gradient was not significant for the CWE of EBWPs. Regions with high endemism were distributed mainly in the north of Xiaoxinganling, south of Qinghai-Tibet Plateau, Hengduan Mountains, southeast Yunnan Province, Hainan Island, and Taiwan Island (Fig. 1b). There was also a decreasing trend in PD_rel_ as the latitude increased. The regions with high PD_rel_ were distributed mainly in southwest China and the regions with low PD_rel_ were distributed mainly in north and west China. It should be mentioned that some areas with relatively low SR for EBWPs, such as Qinling Mountains, had a higher PD_rel_, which reflected the distant phylogenetic relationships between the species in these regions (Fig. 1c). The regions with high PE were distributed mainly in the south Qinghai-Tibet Plateau, Hengduan Mountains, Sichuan Basin, the south and east of Yunnan-Guizhou plateau, Nanling Mountains, Hainan Island, and Taiwan Island (Fig. 1d).

### Distribution patterns of hotspots

**Figure 2:**
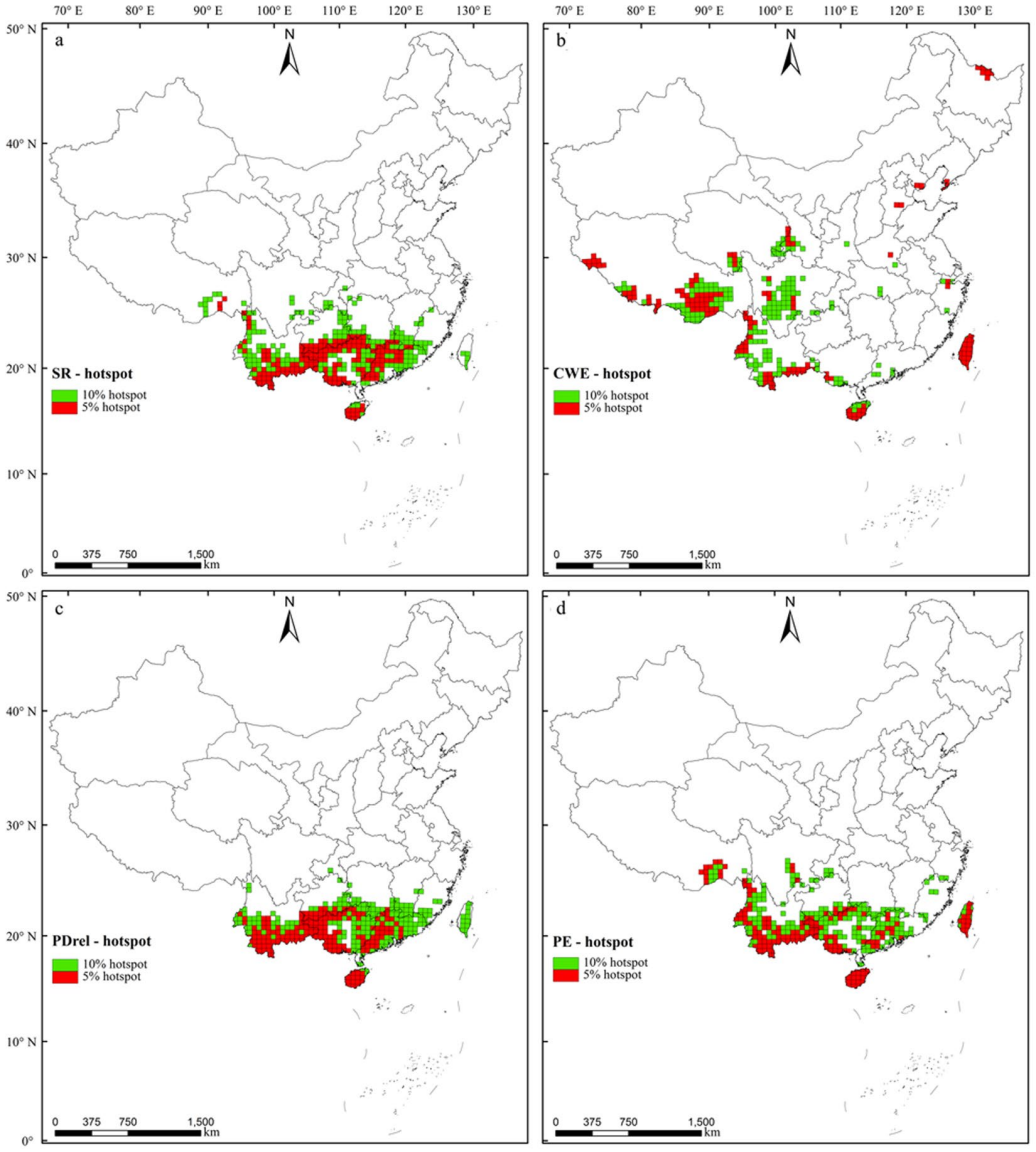
Geographic distribution of hotspots identified by (**a**) SR, (**b**) (CWE, (**c**) PD_rel_, and (**d**) PE for Chinese EBWPs. For each diversity index, the hotspots were defined with the local maximization method at the 5% (red) or 10% (green) levels of the grids. Albers equal-area conic projection. The map was generated using ArcGIS 10.1 (ESRI, Redlands, CA, USA; http://www.esri.com).

According to the results for the 5% hotspots and 10% hotspots, 380 and 700 grids (Fig. 2), respectively, were identified as hotspots by at least one diversity index. The total areas of these hotspots were 7.76 × 10^5^ km^2^ and 1.51 × 10^6^ km^2^, i.e., 8.08% and 15.73% of China’s total land area, respectively. The hotspot complexes contained 5,823 and 6,163 EBWPs, i.e., 92.93% and 98.36% of the total, respectively. Considering that the area coverage for the current nature reserves in China was similar to the total area of the 10% hotspots, we only provide the detailed results for the10% hotspots in the following.

There were great differences in the geographic locations of the hotspots identified by each index (Fig. 2). SR hotspots were mainly linked together and distributed to the south of 30°N. These grids were distributed mostly in the northern part of the Yunnan-Guizhou Plateau, Nanling Mountains, Wuyi Mountains, and Hainan Island (Fig. 2a). CWE hotspots were distributed widely, but they were more prevalent in southwest China. CWE hotspots were distributed mainly in the south Tibetan Plateau, Hengduan Mountains, southwestern and southeastern Yunnan Province, Hainan Island, and Taiwan Island. In addition, a few grids were located in the northern part of the Northeast China Plain and North China Plain (Fig. 2b). PD_rel_ hotspots were all found south of 25°N and they agreed with the SR hotspots, except for some scattered grids (Fig. 2c).The PE hotspots were distributed mainly in the southeast Qinghai-Tibet Plateau, Hengduan Mountains, south Yunnan-Guizhou Plateau, Nanling Mountains, Wuyi Mountains, Hainan Island, and Taiwan Island (Fig. 2d).

Among the 10% hotspots, the hotspots identified using SR, CWE, PD_rel_, and PE contained 5,943, 5,733, 5,545, and 5,996 of the EBWPs, respectively. In addition, the similarity of the species composition in the hotspots identified using different indices was all higher than 90%. The numbers of distinct species in hotspots defined by the SR, CWE, PD_rel_, and PE indices were17, 118, four, and nine, respectively. These 148 species were all narrow-range species, 92.57% of which were distributed in less than 50 grids. This reflects the mutual supplementary nature of each diversity indicator, especially the significance of CWE for protecting range-restricted species. The greatest spatial overlaps between pairs of metrics were for: (1) SR and PD_rel_ (85%), (2) SR and PE (81%), and (3) PD_rel_ and PE (80%). However, the spatial overlap between CWE and SR, PD_rel_, PE was 36%, 34% and 50%, respectively.

**Figure 3:**
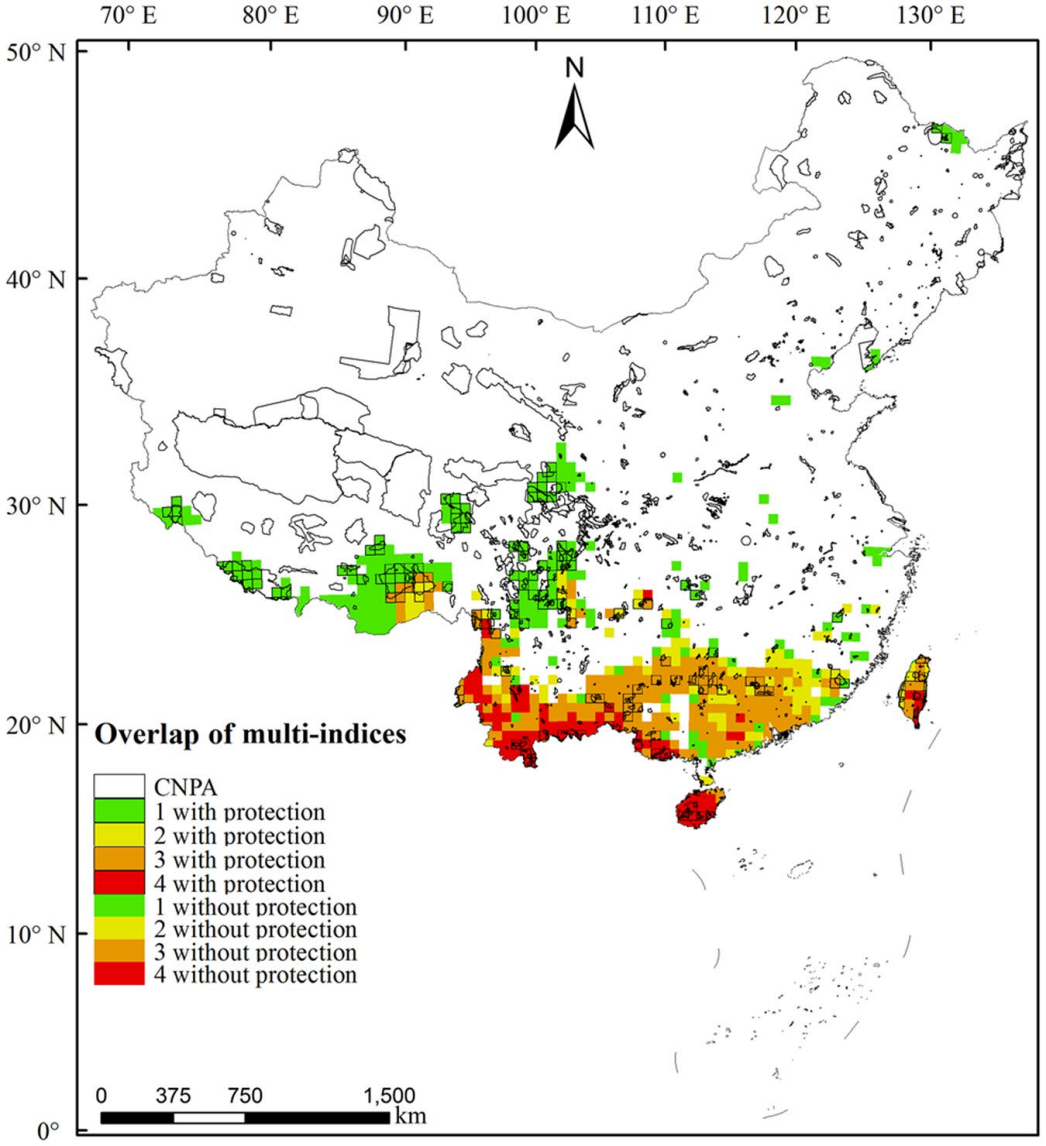
Overlaps of 10% hotspots among the four diversity metrics for Chinese EBWPs. Hotspots with protection denote overlap areas for hotspots and nature reserves exceed10% of the total area of the hotspot, and vice versa. Albers equal-area conic projection. The map was generated using ArcGIS 10.1 (ESRI, Redlands, CA, USA; http://www.esri.com).

We identified 113 grids in the south Hengduan Mountains, south Yunnan-Guizhou Plateau, Hainan Island, and central and southeast Taiwan Island as hotspots using all four diversity indices (Fig. 3). These grid cells had a total area of 2.05 × 10^5^ km^2^, which comprises 2.14% of the total area of China. In total, the 5,006 EBWPs in these hotspots accounted for 79.89% of the total EBWPs in China. In addition, only 5.64% of the grids were identified as hotspots by three metrics and only 2.68% by two metrics.

### Distribution patterns of conservation gaps

**Figure 4:**
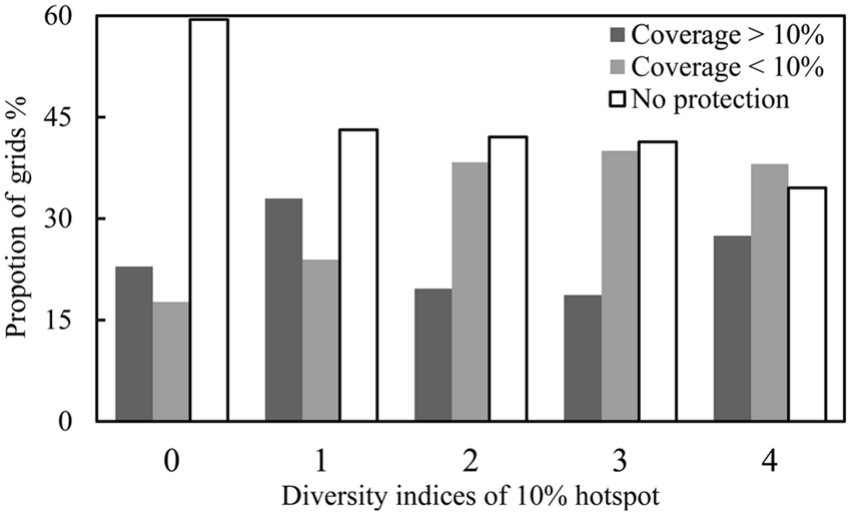
Summary plot of the areal proportion of the study area where overlaps occurred between the four metrics. Numbers on the x-axis represent the hotspots identified with one, two, three, and four diversity indices.

The spatial distributions of the current nature reserves and hotspots for EBWPs in China differed significantly (Fig. 3).These reserves and hotspots had common areas of 1.62 × 10^5^ km^2^, which comprise 1.68% of the total area of China and 12.97% of the total area of China’s nature reserves. In addition, 41.00% of the hotspots were not covered by nature reserves, and 33.57% of the hotspots were covered by nature reserves with less than 10% of the grid area. Only 25.43% of the hotspots were covered by nature reserves with more than 10% of the grid area. Therefore, most of the hotspots for Chinese EBWPs are not well protected. The conservation gaps for hotspots according to four, three, two, and one diversity indices comprised 72.57%, 81.33%, 80.37%, and 67.06% of the hotspot areas, respectively (Fig. 4).

## Discussion

**Figure 5:**
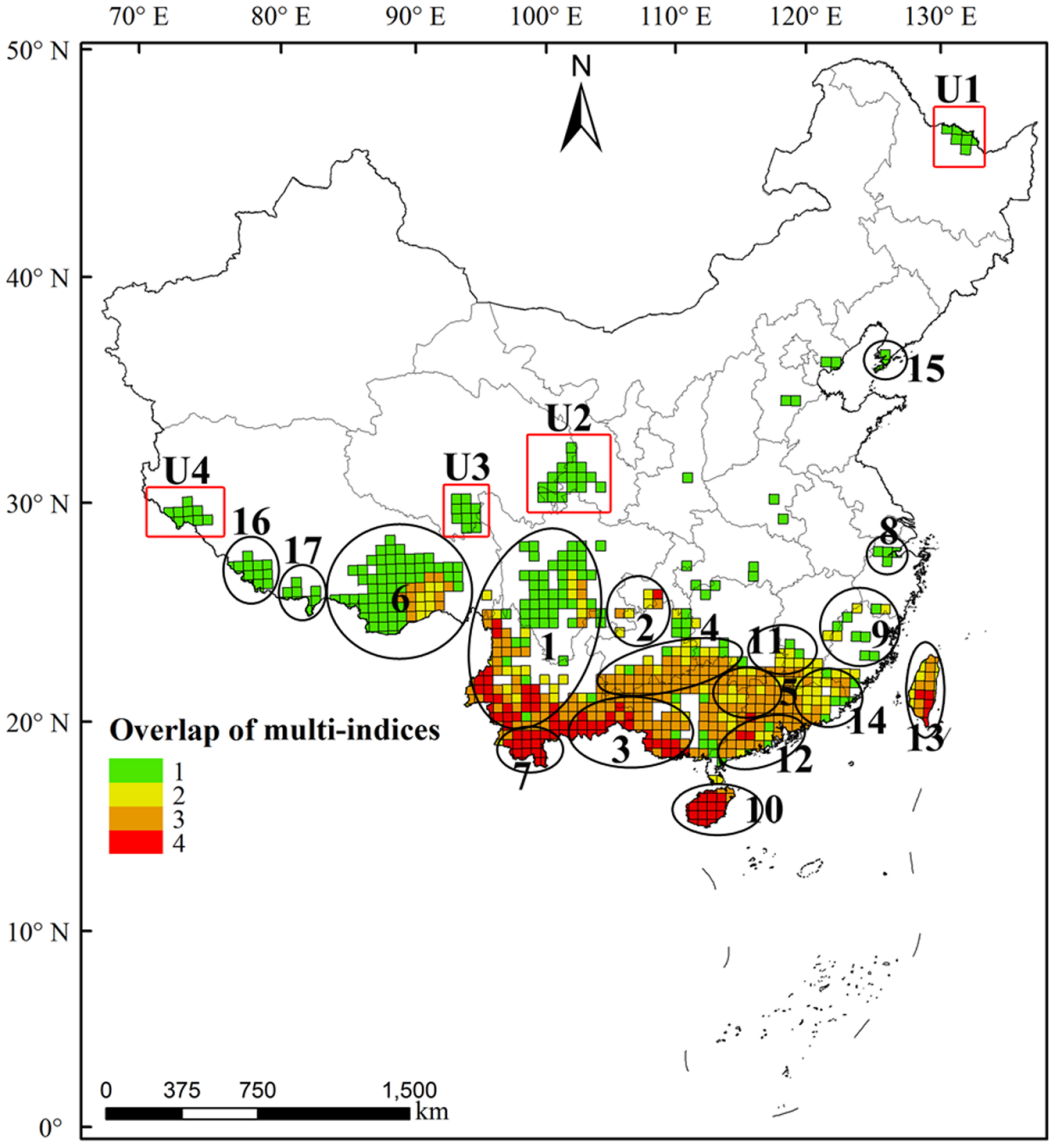
Geographic distributions of hotspots for Chinese EBWPs. Red squares are unique hotspots identified in the present study. Albers equal-area conic projection. The map was generated using ArcGIS 10.1 (ESRI, Redlands, CA, USA; http://www.esri.com).

Twenty-one hotspots in total were identified based on Chinese EBWPs. The hotspots identified in this study covered most of the key areas for biodiversity conservation defined previously in China. Seventeen hotspots coincided with the hotspots identified based on Chinese endemic woody seed plants^21^. These 17 hotspots areas follows (Fig. 5 and Supplementary Table S1): 1. Hengduan Mountains; 2. mountainous areas of south Chongqing; 3. mountainous areas of east Yunnan and west Guangxi; 4. mountainous areas of north Guangxi, south Guizhou, and southwest Hunan; 5. Nanling Mountains; 6. mountainous areas of southeast Tibet; 7. Xishuangbanna region; 8. mountainous areas of north Zhejiang; 9. mountainous areas of south Zhejiang, northwest Fujian, and southeast Jiangxi; 10. Hainan Island; 11. mountainous areas of west Jiangxi and east Hunan; 12. mountainous areas of central and west Guangdong; 13. Taiwan Island; 14. mountainous areas of south Fujian and north Guangdong; 15. Changbai Mountain; 16. Nielamu region; and 17. Yadong region. We also identified four unique hotspots in the present study (Fig. 5): U1. north of Xiaoxinganling; U2. west of Longzhong plateau; U3. origin of Lancang River; U4. northwest of Himalaya Mountains. These four hotspots identified mainly by CWE were distributed in different regions of north China, which contained nine, 56, 34, and six EBWPs, respectively. The species in these hotspots were range-restricted shrubs, 62.07% of which belonged to families such as Ericaceae, Viscoideae, Fagaceae, Oleaceae, and Berberidaceae. We should point out that endemism is a scale-dependent and relative concept which is closely related to study area^36^. Therefore, the identification of endemism hotspots also depends on research area. Physical geography regions barely have clear boundaries, meanwhile biodiversity conservation measures are implemented mainly on a country basis. Consequently endemism hotspot in this research are identified only in China’s administrative borders, which may cause underestimated range size of species and high endemism on the Chinese borders such as in the U1 hotspot.

In addition, the hotspots defined in this study covered 13 of the 14 key terrestrial areas for the conservation of biodiversity in China using extraordinary species and genetic diversity along with high richness of endemic or threatened species^37^; all three hotspots defined using Chinese seed plants based on published floristic data^28^; nine of the 10 hotspot ecoregions identified by county level plant genus and vertebrate species distribution databases^18^; and all eight hotspots for threatened plants in China^20^. The hotspots also covered 16 of the 19 biodiversity hotspots identified based on Chinese endemic seed plants^22^, including the Longzhong plateau hotspot and the origin of Lancang River hotspot. The consistent results obtained in this and previous studies indicate that EBWPs comprise a valuable indicator group for identifying biodiversity hotspots to facilitate conservation in China, they also emphasize the importance of the area south of latitude 35°N (the main distribution area for EBWPs) for biodiversity protection in China.

The intersections between the hotspots obtained by all four indices are very important for biodiversity protection because all the values of the indices were highest in these hotspots. However, hotspots defined by one or several indicators are also important because different indicators highlight different aspects of biodiversity, such as SR, geographic range, and phylogeny^21^. For example, targeting SR hotspots would protect the greatest number of species per unit area. Alternatively, if the goal is to maintain areas with ancient flora, PD_rel_ hotspots would be appropriate targets. Focusing management on CWE hotspots would stress the conservation of range-restricted species. Areas with high endemism may include many species with a small geographic distribution, where the similarity of the species composition is low compared with other regions. PE hotspots including lineages that are both evolutionarily distinct and geographically restricted may be a great conservation concern^38^.These distinctive hotspots are likely to be neglected if we only consider one aspect when identifying hotspots. For example, compared with the hotspots identified by SR and PD_rel_, those identified using CWE and PE included Taiwan Island. Thus, measures that incorporate the geographic ranges of species are important for selecting hotspots with fewer total species but relatively high proportion of narrow-ranged species.

It has been suggested that focusing on hotspots is the most cost-efficient approach to biodiversity conservation by targeting efforts at regions with the most vulnerable species^13, 14^. However, biodiversity can be defined in so many ways that there may be little overlap among the hotspots for different taxa or for hotspots defined by different diversity indices, thereby making it difficult to identify the ranges and boundaries of hotspots^39, 40^. For example, by using the species density, species evenness, taxonomic distinctness, functional divergence, and total biomass to map demersal fish hotspots in the California Current Large Marine Ecosystem, it was found that there was minimal spatial overlap among hotspots for the five indices and no area was identified as a hotspot by all five metrics^16^. We also showed that only 16.14% of the 10% hotspots were identified by all of the four metrics used in this study. Therefore, we must emphasize that it is necessary to define a clear and agreed objective before choosing relevant metrics for a specific problem.

The species richness and composition, and the values of the diversity indicators varied greatly among the different hotspots for EBWPs in China (Supplementary Table S1). This is because the 21 hotspots identified by different indices varied in their underlying conservation importance. For example, the Hengduan Mountains hotspot was one of the hotspots^14^ identified earliest in global hotspot research, and it also had the most EBWPs among all 21 of our hotspots. Furthermore, the species/families ratio (28.16) and species/genus ratio (5.64) were both highest in Hengduan Mountains, which indicates that this hotspot is one of the regions with the strongest species diversification for EBWPs. Due to the uplift of the Qinghai-Tibet plateau from the Eocene to the Pliocene and climatic fluctuations within the glacial/inter-glacial cycle, the environmental conditions in this area may have been most suitable for the evolution of modern EBWPs^41, 42^. Comparing the two island hotspots, the SR in Taiwan Island (739) was significantly lower than that in Hainan Island (1,365), but the mean CWE of the former (2.48) was higher than that of the latter (1.95). These differences might be related to the geological history and geographic locations of these two islands. Taiwan Island is further from the mainland and it separated from the continent earlier. Moreover, the latitude of Taiwan Island is further north than Hainan Island, with more temperate genera among the flora. By contrast, Hainan Island is near to the relic center of endemic plants in southeast Yunnan-west Guangxi, and it was in contact with the mainland for several times due to the growth and decline of glaciers. Consequently, the percentage of EBWPs in Taiwan Island is much lower than that in Hainan Island, but the extent of the endemic flora in Taiwan is more complex than that in Hainan^43^.The Xishuangbanna hotspot had the greatest number of species per grid (149.5 species/grid cell) and it was the only hotspot where all the grid cells were identified by all four diversity metrics.

China has already established a large number of nature reserves, but the efficiency of these reserves is not ideal^33, 44^,which is mainly due to weaknesses in the programming and construction of nature reserves. Moreover, we found that most of the hotspots for Chinese EBWPs were protected poorly by the current network of nature reserves (Supplementary Fig. S1). The existing nature reserves cover almost 15% of China’s total land area but the area covered is very limited for EBWPs hotspots, especially in southeastern China. Thus, there are still considerable conservation gaps among the hotspots for Chinese EBWPs. Consequently, we suggest that the effectiveness of the established nature reserves should be improved and new protection areas should be planned in the conservation gap areas for Chinese EBWPs. In particular, we make the following suggestions: (1) For hotspots with better protection and less gaps, but that still harbor inherently threatened species, such as H16 and U3, the strategies should focus on *in situ* conservation, population management, and range size expansion for the species distributed within these locations. (2) The hotspots undergoing severe spatial fragmentation in southeastern China, such as H5, H8, H9, H11, H12, and H14, may have a high risk of local extinction and rapid degradation of ecosystem functions. Reducing habitat disturbance and reconstructing dispersal corridors are the preferred conservation strategies for these areas. (3) For the scattered hotspots identified mainly by species endemism, such as H6, H15, H16, U1, U2, U3, and U4, *ex situ* strategies are required to protect biodiversity, as well as the development of propagation and reintroduction programs because numerous narrow-range and vulnerable species are distributed in these regions. (4) For the small and scattered hotspots, such as H2, H9, and the hotspot grids distributed in central China, relatively low-level alternate priority areas should also be considered if time and resources permit. (5) Hotspots in southwestern China, such as H1, H3, and H7, are predicted to undergo substantial change and spatially shifted bioclimatic conditions^45^. Therefore, a non-static nature reserve network could buffer against the potential impacts of climate change. The boundaries and ranges of nature reserves should be adjusted accordingly to monitor species migration on an ongoing basis.

## Methods

### Data set

The species distribution data were taken from the “*Atlas of Woody Plants in China: Distribution and Climate*,” published in 2011^46^, which is now the most complete atlas of woody seed plants in China and contains county level distribution maps for all 11,405 native woody species. The species of evergreen broadleaved woody plants (EBWPs) was selected from the atlas using information from the Flora of China (http://www.efloras.org/), Higher Plants in China^47^, and suggestions from taxonomic experts. Finally, a database of 6,265 EBWPs were built for the following analyses. There are 2,408 county-level units in total, which had a median area of 2,081 km^2^ in the database^48^. To eliminate the effect of area on the estimation of diversity, maps based on grid cells of 50 km × 50 km, which is approximate to the median area of counties were used as spatially operational geographic units. We excluded units with a land area smaller than 1,250 km^2^ because grid cells located along coasts or on borders are often incomplete.

We used Phylomatic (http://phylodiversity.net/phylomatic/) and employed the Angiosperm Phylogeny Group III classification as a backbone to construct a phylogenetic supertree for the Chinese EBWPs. The branch lengths in the phylogenetic tree were adjusted using the BLADJ algorithm with the differentiation time for angiosperm plants^49^.This approach has been used widely in analyses of large-scale spatial patterns of plant phylogenetic diversity^50, 51^.

A recently updated version of the map of nature reserves in China was digitized to indicate the current conservation status in China. The dataset was integrated from the most recent official list of nature reserves published by China Ministry of Environment Protection and ProtectedPlanet.net (http://www.protectedplanet.net/). Each nature reserve is administered by one of three different levels of government departments: national, provincial, and municipal or prefectural. The nature reserve system used in this study covered a total area of 1.25 × 10^6^ km^2^, which is about 13.1% of China’s total land area.

### Diversity indices

SR is the universal index generally employed in biodiversity studies^52^. In the present study, the total count of EBWPs within a grid cell was defined as SR.

Increasing evidences indicate that SR hotspots are not the same as endemism hotspots all the time^31, 53, 54^. Thus, concentrations of local endemic species may exist in areas with low richness^54^. In order to exclude the effect of widespread species on the identification of biodiversity hotspots, we also calculated the spatial pattern of endemism, which contained large numbers of rare and endangered range-restricted species^55^. It is generally acknowledged that a sampling effect due to species numbers can lead to high correlations between richness and endemism, so we employed corrected weighted endemism (CWE) to represent species endemism, which is defined as:

1 $${\rm{CWE}}=\sum _{{\rm{j}}=1}^{S}\frac{1}{{R}_{{\rm{j}}}}/S\times 100 \% ,$$

where *S* is the number of taxa (i.e., EBWPs in this study) in a grid cell and *R*_j_ is the range size of the j_th_ species in a grid cell, which is the sum of the grid cells where the focal species is recorded.

Species are not all equal and more evolutionarily distinct species should be considered more valuable^10^. By integrating SR and phylogenetic information, phylogenetic diversity (Faith’s PD) can comprehensively assess the biodiversity information for study taxa and this measure is used widely in biodiversity pattern analysis and priority area identification^56–58^. PD calculates the sum of the lengths of all branches in a grid, so when there are increasing numbers of terminal taxa, the correlation between PD and SR becomes increasingly stronger and the evolutionary information may be obscured^59^. Therefore, we conducted an analysis where we standardized the PD value by the logarithmical taxa number, which we defined as the relative phylogenetic diversity (PD_rel_). Due to the over-dispersed variation of SR values for the EBWPs, ranging 1–1843 across grid cells of calculation, the equation for PD_rel_ from Davis *et al*.^60^ was modified as follows:

2 $${{\rm{PD}}}_{{\rm{rel}}}={\rm{Faith}}\mbox{'}{\rm{s}}\,{\rm{PD}}/{\mathrm{Log}}_{10}S,$$

where *S* is the number of taxa (i.e., EBWPs in this study) in a grid, Faith’s PD is the sum of the lengths of all branches that are members of the corresponding minimum spanning path.

PD_rel_ focuses on the phylogenetic features of a taxonomic group, but it ignores the spatial distribution range of taxa, which is an important factor that needs to be considered when identifying conservation priorities. Thus, a new index defined as phylogenetic endemism (PE) was developed to combine both evolutionary and spatial features^58^. PE measures relative changes in the distribution range of plant taxa, but it also reflects the dispersion level among taxa on the phylogenetic tree. The equation for calculating PE is as follows:

3 $${\rm{PE}}=\sum _{{\rm{c}}\in {\rm{C}}}\frac{{L}_{{\rm{c}}}}{{R}_{{\rm{c}}}},$$

where C is the set of branches in the path connecting the taxa to the root of the tree, c is a branch in path C, *L*_c_ is the length of branch c, and *R*_c_ is the clade range, which is defined as the total range of the taxa descended in the phylogeny tree from branch c and occurring in a grid, where overlapping areas are counted only once.

### Identifying biodiversity hotspots

We compared the maps using the four metrics to identify locations with high diversity. Any given 50 km × 50 km grid cell was marked as a hotspot if the value for a given index was in the top 5% or 10% of its range (referred to as 5% and 10% hotspots, respectively, in the following)^16, 18, 21, 22^. We produced maps of the overlapping between the 10% hotspots in order to illustrate the grid cells with multi-metric hotspots. Finally, we overlaid the hotspot maps with the distribution of nature reserves to identify conservation gap areas for the hotspots of EBWPs across China. According to international conservation organizations, each type of ecosystem should have 10% to 12% of the land area under protection to ensure effective conservation^61, 62^. Thus, we identified conservation gaps as hotspot grid cells with nature reserve coverage of less than 10% of the grid cell area.

We calculated the four diversity indices for each grid cell using two R packages, “Ape” and “Picante” in R 3.2.3 (R Core Team; available at http://www.r-project.org/), and we generated the maps of the diversity patterns using ArcGIS 10.0 (ESRI, Redlands, CA, USA).

## Electronic supplementary material

Supplementary information

Dataset 1

Dataset 2
