# Supplementary material for: Hotspot analyses indicate significant conservation gaps for evergreen broadleaved woody plants in China
Source: Sci Rep. 2017 May 12;7:1859. doi: 10.1038/s41598-017-02098-0 (PMC5431964; doi:10.1038/s41598-017-02098-0)
Supplement: Supplementary file 1 — Supplementary information [file 41598_2017_2098_MOESM1_ESM.pdf]

Hotspot analyses indicate conservation gaps for evergreen broadleaved woody plants in China

Yue Xu[1,2], ZeHao Shen [1,*], LingXiao Ying[1], Zhiheng Wang[1], Jihong Huang[2,3], Runguo Zang[2,3] & YouXu Jiang[2]

[1]Department of Ecology, MOE Key Laboratory on Earth Surface Processes, College of Urban and Environmental Science, Peking University, Beijing 100871,China

[2]Institute of Forest Ecology, Environment and Protection, Chinese Academy of Forestry, Beijing 100093, China

[3]Co-Innovation Center for Sustainable Forestry in Southern China, Nanjing Forestry University, Nanjing 210037, China

Appendix S1 Basic statistical information for the 21 hotspots.

Hotspots	Grids	Species	Threatened species Number (percentage %)	Families	Genus	SR		CWE		Pd_{rel}		PE	
						Min	Max	Min	Max	Min	Max	Min	Max
H1	127	3210	656(20.44)	114	569	19	1410	0.74	2.57	559.24	2245.87	13.32	1204.89
H2	8	986	114(11.56)	84	241	516	675	0.97	1.28	1510.83	1538.36	243.50	344.01
H3	86	2553	638(24.99)	120	728	457	1843	0.46	2.92	1472.18	2328.27	138.87	2186.64
H4	84	2479	617(24.89)	114	478	394	1108	0.47	1.13	1555.42	1908.32	146.07	466.05
H5	37	1487	172(11.57)	99	350	686	1006	0.56	0.96	1690.26	1879.20	218.74	426.04
H6	86	897	174(19.40)	83	233	5	803	1.21	12.10	159.08	1488.76	2.95	503.48
H7	14	1547	296(19.13)	108	486	1079	1444	1.58	1.95	2128.93	2279.42	895.12	1335.28
H8	5	297	14(4.71)	55	121	8	250	1.24	1.84	489.34	1325.61	4.73	229.62
H9	11	938	78(8.32)	93	266	411	596	0.51	1.24	1441.18	1642.36	152.96	328.39
H10	19	1365	245(17.95)	109	451	512	1243	1.28	3.24	1760.72	2150.00	405.87	1772.22
H11	20	1412	159(11.26)	97	325	541	966	0.49	0.78	1558.38	1850.74	160.44	361.48
H12	51	1792	247(13.78)	109	438	458	998	0.64	1.50	1619.56	1976.18	189.66	683.65
H13	22	739	77(10.42)	96	303	189	660	1.56	4.11	1286.51	1782.78	181.89	1270.05
H14	51	1191	128(10.75)	97	329	486	753	0.46	0.82	1571.25	1758.37	144.54	271.09
H15	6	11	0(0.00)	9	9	11	11	1.66	1.66	672.16	672.16	37.46	37.46
H16	14	139	17(12.23)	42	72	60	124	1.44	1.92	924.60	1011.27	91.36	147.08
H17	11	104	12(11.54)	26	48	55	72	1.59	1.89	694.00	777.56	51.58	108.94
U1	8	9	3(33.33)	4	8	7	10	1.64	2.47	312.24	412.24	17.20	18.78
U2	18	56	4(7.14)	20	27	11	28	1.21	3.52	399.62	668.54	6.79	30.03
U3	10	34	1(2.94)	14	16	16	32	1.32	1.78	368.26	668.09	8.91	24.26
U4	12	6	0(0.00)	6	6	4	5	1.89	2.29	492.75	550.55	24.50	25.26

* Threatened species were identified using China Biodiversity Red List (http://www.zhb.gov.cn/gkml/hbb/bgg/201309/t20130912 _260061.htm).

Appendix S2 List of China’s major mountain ranges[1].

No.	Name	No.	Name
1	Daxing'anling Mountains	34	Danggula Mountains
2	Xiaoxing'anling Mountains	35	Gangdisi Mountains
3	Changbai Mountains	36	Nyainqntanglha Mountains
4	Zhangguangcailing Mountains	37	Himalayas
5	Longgang Mountains	38	Boshula Mountains
6	Yinshan Mountains	39	Gaoligong Mountains
7	Yanshan Mountains	40	Taniantaweng Mountains
8	Daqing Mountains	41	Nushan Mountains
9	Altai Mountains	42	Yunling Mountains
10	Poluokenu Mountains	43	Shaluli Mountains
11	South branch of Tianshan Mountains	44	Daxue Mountains
12	Tianshan Mountains	45	Qionglai Mountains
13	Bogeda Mountains	46	Wulian Mountains
14	Taihang Mountains	47	Wumeng Mountains
15	Luliang Mountains	48	Ailao Mountains
16	Zhongtiao Mountains	49	Wuliang Mountains
17	Helan Mountains	50	Wuling Mountains
18	Liupan Mountains	51	Xuefeng Mountains
19	Aerjin Mountains	52	Dalou Mountains
20	Qilian Mountains	53	Mufu Mountains
21	Qinghainan Mountains	54	Jiuling Mountains
22	Laji Mountains	55	Luoxiao Mountains
23	Kunlun Mountains	56	Daiyun Mountains
24	Animaqin Mountains	57	Xianxialing Mountains
25	Kekexili Mountains	58	Tianmu Mountains
26	Bayankala Mountains	59	Yandang Mountains
27	Minshan Mountains	60	Wuyi Mountains
28	Qinling Mountains	61	Nanling Mountains
29	Funiu Mountians	62	Dayao Mountains
30	Daba Mountains	63	Yunwu Mountains
31	Wudang Mountains	64	Yushan Mountains
32	Dabie Mountains	65	Wuzhi Mountains
33	Karakorum Mountains		

Appendix S3. Locations of main mountain ranges in China. Black numbers indicate main mountain ranges (Numbers are consistent with Supplementary Table S2). The map was generated using ArcGIS 10.1 (ESRI, Redlands, CA, USA; http://www.esri.com).

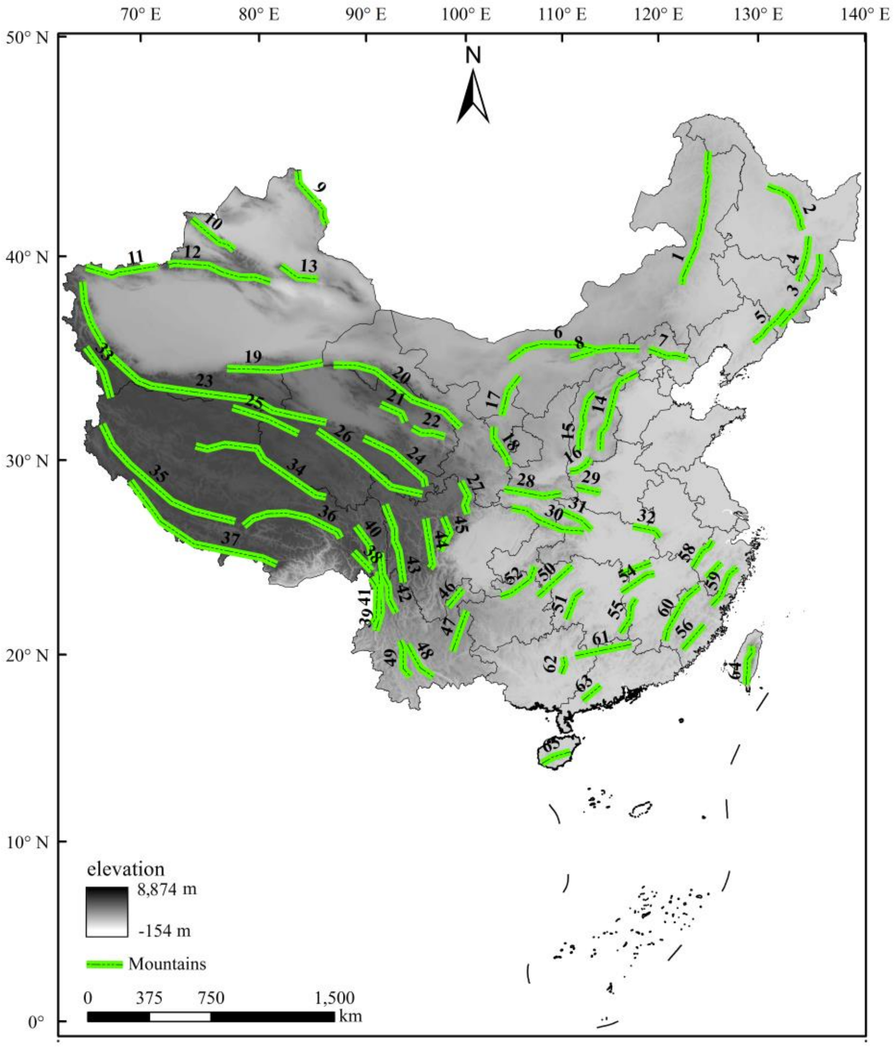

Appendix S4 Percentage of multi-index hotspots (a) and the protection status (b) of the 21 hotspots identified for Chinese evergreen broadleaved woody plant species.

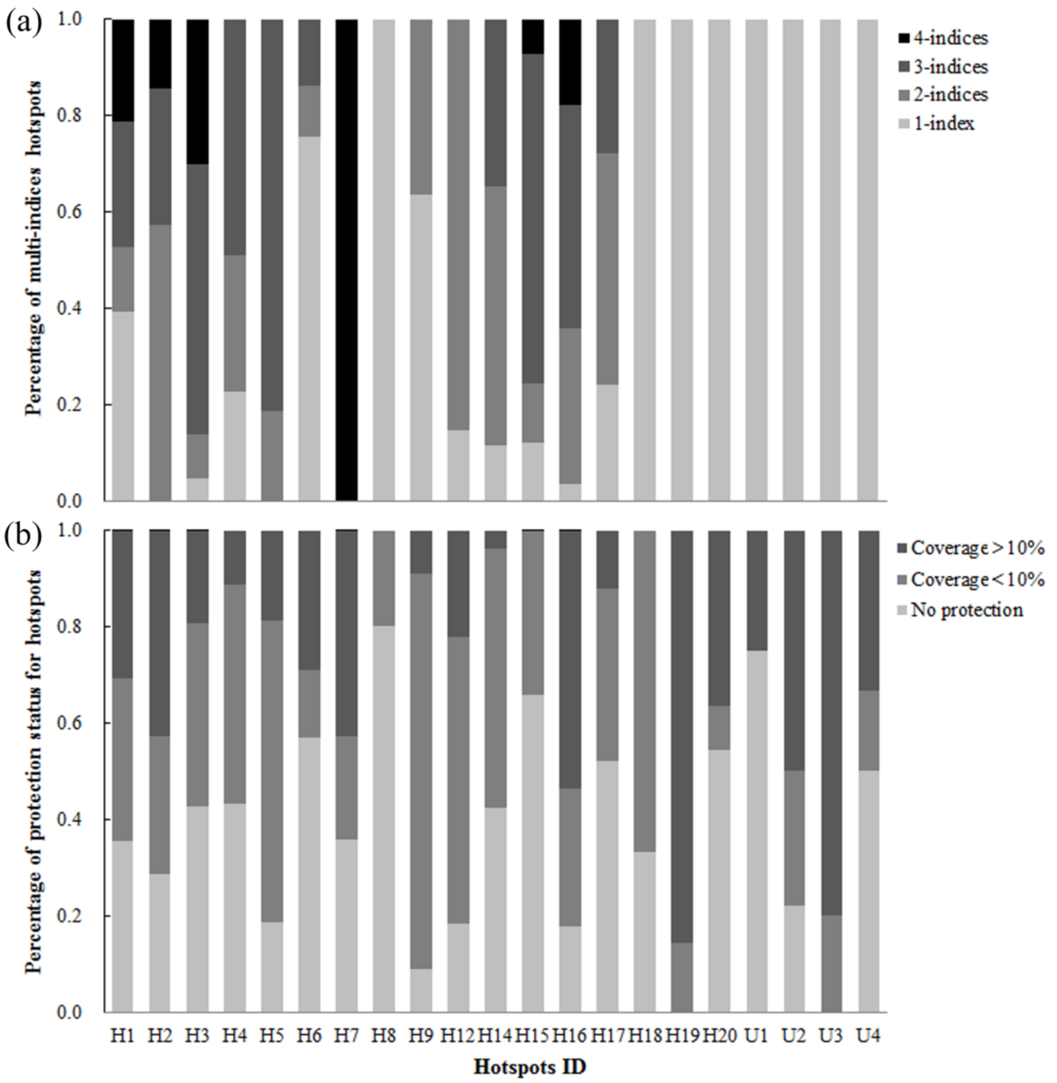

Reference

1. Wang, X. P., Wang, Z. H., Fang, J. Y. Mountains ranges and peaks in China. *Biodivers. Sci.* **12**, 206–212 (2004).
