# Supplementary material for: Hotspot analyses indicate significant conservation gaps for evergreen broadleaved woody plants in China
Source: Sci Rep. 2017 May 12;7:1859. doi: 10.1038/s41598-017-02098-0 (PMC5431964; doi:10.1038/s41598-017-02098-0)
Supplement: Supplementary file 3 — Dataset 2 [file 41598_2017_2098_MOESM3_ESM.doc]

Appendix **S3**. The phylogenetic tree of Chinese evergreen broadleaved woody plant species (EBWPs).

((((((((((((((((((((((Gigantochloa_felix:56.521740,Gigantochloa_nigrociliata:56.521740,Gigantochloa_levis:56.521740,Gigantochloa_parviflora:56.521740,Gigantochloa_albociliata:56.521740)Gigantochloa:56.521740,(Acidosasa_notata:56.521740,Acidosasa_chienouensis:56.521740,Acidosasa_lingchuanensis:56.521740,Acidosasa_purpurea:56.521740,Acidosasa_chinensis:56.521740,Acidosasa_nanunica:56.521740)Acidosasa:56.521740,(Ampelocalamus_microphyllus:56.521740,Ampelocalamus_hirsutissimus:56.521740,Ampelocalamus_calcareus:56.521740,Ampelocalamus_mianningensis:56.521740,Ampelocalamus_melicoideus:56.521740,Ampelocalamus_mnaibunensis:56.521740,Ampelocalamus_sacndens:56.521740,Ampelocalamus_actinotrichus:56.521740,Ampelocalamus_luodianensis:56.521740,Ampelocalamus_saxatilis:56.521740,Ampelocalamus_yongshanensis:56.521740)Ampelocalamus:56.521740,(Chimonobambusa_szechuanensis:56.521740,Chimonobambusa_purpurea:56.521740,Chimonobambusa_pachystachys:56.521740,Chimonobambusa_damingshanensis:56.521740,Chimonobambusa_grandifolia:56.521740,Chimonobambusa_macrophylla:56.521740,Chimonobambusa_brevinoda:56.521740,Chimonobambusa_quadrangularis:56.521740,Chimonobambusa_luzhiensis:56.521740,Chimonobambusa_marmorea:56.521740,Chimonobambusa_hejiangensis:56.521740,Chimonobambusa_macrophylla_var.leiboensis:56.521740,Chimonocalamus_fimbriatus:56.521740,Chimonobambusa_hirtinoda:56.521740,Chimonobambusa_armata:56.521740,Chimonobambusa_metuoensis:56.521740,Chimonobambusa_communis:56.521740,Chimonobambusa_tumidissinoda:56.521740,Chimonobambusa_puberula:56.521740,Chimonobambusa_lactistriata:56.521740,Chimonobambusa_opienensis:56.521740,Chimonobambusa_pubescens:56.521740,Chimonobambusa_rigidula:56.521740,Chimonocalamus_griffithianus:56.521740,Chimonobambusa_hsuehiana:56.521740,Chimonobambusa_angustifolia:56.521740,Chimonobambusa_convoluta:56.521740,Chimonobambusa_microfloscula:56.521740,Chimonobambusa_sichuanensis:56.521740)Chimonobambusa:56.521740,(Melocalamus_arrectus:56.521740,Melocalamus_compactiflorus:56.521740,Melocalamus_elevatissimus:56.521740)Melocalamus:56.521740,(Melocanna_baccifera:56.521740)Melocanna:56.521740,(Chimonocalamus_pallens:56.521740,Chimonocalamus_makuanensis:56.521740,Chimonocalamus_montanus:56.521740,Chimonocalamus_delicatus:56.521740,Chimonocalamus_dumosus:56.521740,Chimonocalamus_longiusculus:56.521740,Chimonocalamus_longiligulatus:56.521740)Chimonocalamus:56.521740,(Drepanostachyum_fractiflexum:56.521740,Drepanostachyum_semiorbiculatum:56.521740,Drepanostachyum_ampullare:56.521740)Drepanostachyum:56.521740,(Himalayacalamus_collaris:56.521740,Himalayacalamus_falconeri:56.521740)Himalayacalamus:56.521740,(Bambusa_basihirsuta:56.521740,Bambusa_sinospinosa:56.521740,Bambusa_pervariabilis:56.521740,Bambusa_emeiensis:56.52174,Bambusa_pallida:56.52174,Bambusa_macrotis:56.52174,Bambusa_grandis:56.52174,Bambusa_beecheyana_var.pubescens:56.52174,Bambusa_eutuldoides:56.52174,Bambusa_cerosissima:56.52174,Bambusa_diaoluoshanensis:56.52174,Bambusa_variostriata:56.52174,Bambusa_beecheyana:56.52174,Bambusa_corniculata:56.52174,Bambusa_chungii:56.52174,Bambusa_ventricosa:56.52174,Bambusa_tulda:56.52174,Bambusa_glabro-vagina:56.52174,Bambusa_guangxiensis:56.52174,Bambusa_pervariabilis_var.viridistriata:56.52174,Bambusa_albo-lineata:56.52174,Bambusa_chunii:56.52174,Bambusa_stenoaurita:56.52174,Bambusa_mutabilis:56.52174,Bambusa_polymorpha:56.52174,Bambusa_funghomii:56.52174,Bambusa_remotiflora:56.52174,Bambusa_subaequalis:56.52174,Bambusa_blumeana:56.52174,Bambusa_insularis:56.52174,Bambusa_distegia:56.52174,Bambusa_vulgaris:56.52174,Bambusa_oldhamii:56.52174,Bambusa_boniopsis:56.52174,Bambusa_malingensis:56.52174,Bambusa_multiplex_var.incana:56.52174,Bambusa_bicicatricata:56.52174,Bambusa_pachinensis:56.52174,Bambusa_intermedia:56.52174,Bambusa_burmanica:56.52174,Bambusa_rutila:56.52174,Bambusa_ramispinosa:56.52174,Bambusa_gibba:56.52174,Bambusa_mollis:56.52174,Bambusa_prominens:56.52174,Bambusa_duriuscula:56.52174,Bambusa_contracta:56.52174,Bambusa_tuldoides:56.52174,Bambusa_textilis:56.52174,Bambusa_eutuldoides_var.viridi-vittata:56.52174,Bambusa_piscatorum:56.52174,Bambusa_papillata:56.52174,Bambusa_hainanensis:56.52174,Bambusa_wenchouensis:56.52174,Bambusa_odashimae:56.52174,Bambusa_utilis:56.52174,Bambusa_angustissima:56.52174,Bambusa_angustiaurita:56.52174,Bambusa_xiashanensis:56.52174,Bambusa_indigena:56.52174,Bambusa_flexuosa:56.52174,Bambusa_multiplex:56.52174,Bambusa_subtruncata:56.52174,Bambusa_rigida:56.52174,Bambusa_lapidea:56.52174,Bambusa_surrecta:56.52174,Bambusa_gibboides:56.52174,Bambusa_pachinensis_var.hirsutissima:56.52174,Bambusa_dolichoclada:56.52174,Bambusa_crispiaurita:56.52174,Bambusa_longipalea:56.52174)Bambusa:56.521740,(Phyllostachys_parvifolia:56.521740,Phyllostachys_dulcis:56.521740,Phyllostachys_lofushanensis:56.521740,Phyllostachys_glauca:56.52174,Phyllostachys_virella:56.52174,Phyllostachys_viridi-glaucescens:56.52174,Phyllostachys_nigella:56.52174,Phyllostachys_sulphurea_var.viridis:56.52174,Phyllostachys_prominens:56.52174,Phyllostachys_guizhouensis:56.52174,Phyllostachys_reticulata:56.52174,Phyllostachys_rivalis:56.52174,Phyllostachys_rubromarginata:56.52174,Phyllostachys_iridescens:56.52174,Phyllostachys_rubicunda:56.52174,Phyllostachys_incarnata:56.52174,Phyllostachys_nidularia:56.52174,Phyllostachys_carnea:56.52174,Phyllostachys_glabrata:56.52174,Phyllostachys_angusta:56.52174,Phyllostachys_platyglossa:56.52174,Phyllostachys_nuda:56.52174,Phyllostachys_kwangsiensis:56.52174,Phyllostachys_acuta:56.52174,Phyllostachys_fimbriligula:56.52174,Phyllostachys_sulphurea:56.52174,Phyllostachys_stimulosa:56.52174,Phyllostachys_meyeri:56.52174,Phyllostachys_nigra_var.henonis:56.52174,Phyllostachys_circumpilis:56.52174,Phyllostachys_edulis:56.52174,Phyllostachys_mannii:56.52174,Phyllostachys_rutila:56.52174,Phyllostachys_flexuosa:56.52174,Phyllostachys_aurea:56.52174,Phyllostachys_bissetii:56.52174,Phyllostachys_arcana:56.52174,Phyllostachys_heteroclada:56.52174,Phyllostachys_makinoi:56.52174,Phyllostachys_tianmuensis:56.52174,Phyllostachys_elegans:56.52174,Phyllostachys_vivax:56.52174,Phyllostachys_atrovaginata:56.52174,Phyllostachys_varioauriculata:56.52174,Phyllostachys_robustiramea:56.52174,Phyllostachys_yunhoensis:56.52174,Phyllostachys_propinqua:56.52174,Phyllostachys_violascens:56.52174,Phyllostachys_verrucosa:56.52174,Phyllostachys_nigra:56.52174)Phyllostachys:56.521740,(Sasa_longiligulata:56.521740,Sasa_guangxiensis:56.521740,Sasa_rubrovaginata:56.521740,Sasa_hubeiensis:56.521740,Sasa_sinica:56.521740,Sasa_oblongula:56.521740,Sasa_qingyuanensis:56.521740,Sasa_tomentosa:56.521740)Sasa:56.521740,(Bonia_saxatilis:56.521740,Bonia_levigata:56.521740,Bonia_parvifloscula:56.521740,Bonia_amplexicaulis:56.521740)Bonia:56.521740,(Brachystachyum_densiflorum:56.521740)Brachystachyum:56.521740,(Fargesia_semicoriacea:56.521740,Fargesia_scabrida:56.521740,Fargesia_zayuensis:56.521740,Fargesia_perlonga:56.52174,Fargesia_altior:56.52174,Fargesia_grossa:56.52174,Fargesia_mairei:56.52174,Fargesia_contracta:56.52174,Fargesia_frigida:56.52174,Fargesia_hsuehiana:56.52174,Fargesia_sagittatinea:56.52174,Fargesia_ferax:56.52174,Fargesia_gongshanensis:56.52174,Fargesia_robusta:56.52174,Fargesia_glabrifolia:56.52174,Fargesia_hainanensis:56.52174,Fargesia_melanostachys:56.52174,Fargesia_porphyrea:56.52174,Fargesia_nitida:56.52174,Fargesia_acuticontracta:56.52174,Fargesia_cuspidata:56.52174,Fargesia_caduca:56.52174,Fargesia_jiulongensis:56.52174,Fargesia_circinata:56.52174,Fargesia_edulis:56.52174,Fargesia_extensa:56.52174,Fargesia_farcta:56.52174,Fargesia_dracocephala:56.52174,Fargesia_conferta:56.52174,Fargesia_lushuiensis:56.52174,Fargesia_communis:56.52174,Fargesia_mali:56.52174,Fargesia_dura:56.52174,Fargesia_decurvata:56.52174,Fargesia_concinna:56.52174,Fargesia_plurisetosa:56.52174,Fargesia_fungosa:56.52174,Fargesia_emaculata:56.52174,Fargesia_praecipua:56.52174,Fargesia_albo-cerea:56.52174,Fargesia_qinlingensis:56.52174,Fargesia_rufa:56.52174,Fargesia_denudata:56.52174,Fargesia_pauciflora:56.52174,Fargesia_murielae:56.52174,Fargesia_adpressa:56.52174,Fargesia_obliqua:56.52174,Fargesia_brevissima:56.52174,Fargesia_macclureana:56.52174,Fargesia_hygrophila:56.52174,Fargesia_stenoclada:56.52174,Fargesia_declivis:56.52174,Fargesia_lincangensis:56.52174,Fargesia_canaliculata:56.52174,Fargesia_angustissima:56.52174,Fargesia_papyrifera:56.52174,Fargesia_longiuscula:56.52174,Fargesia_orbiculata:56.52174,Fargesia_pleniculmis:56.52174)Fargesia:56.521740,(Neomicrocalamus_prainii:56.521740,Neomicrocalamus_yunnanensis:56.521740)Neomicrocalamus:56.521740,(Indocalamus_hirsutissimus:56.521740,Indocalamus_emeiensis:56.521740,Indocalamus_wilsoni:56.521740,Indocalamus_quadratus:56.521740,Indocalamus_guangdongensis:56.521740,Indocalamus_longianritus_var.hengshanensis:56.521740,Indocalamus_hunanensis:56.521740,Indocalamus_pseudosinicus:56.521740,Indocalamus_latifolius:56.521740,Indocalamus_hirtivaginatus:56.521740,Indocalamus_decorus:56.521740,Indocalamus_pseudosinicus_var.densinervillus:56.521740,Indocalamus_barbatus:56.521740,Indocalamus_guangdongensis_var.mollis:56.521740,Indocalamus_longiauritus:56.521740,Indocalamus_tessellatus:56.521740,Indocalamus_victorialis:56.521740,Indocalamus_sinicus:56.521740,Indocalamus_longianritus_var.yiyangensis:56.521740,Indocalamus_hispidus:56.521740,Indocalamus_inaequilaterus:56.521740,Indocalamus_herklotsii:56.521740,Indocalamus_bashanensis:56.521740,Indocalamus_longianritus_var.semifalcatus:56.521740)Indocalamus:56.521740,(Indosasa_shibataeoides:56.521740,Indosasa_ingens:56.521740,Indosasa_crassiflora:56.521740,Indosasa_singulispicula:56.521740,Indosasa_gigantea:56.521740,Indosasa_patens:56.521740,Indosasa_spongiosa:56.521740,Indosasa_lipoensis:56.521740,Indosasa_longispicata:56.521740,Indosasa_hispida:56.521740,Indosasa_glabrata:56.521740,Indosasa_angustata:56.521740,Indosasa_triangulata:56.521740,Indosasa_parvifolia:56.521740)Indosasa:56.521740,(Thyrsostachys_siamensis:56.521740)Thyrsostachys:56.521740,(Sinobambusa_farinosa:56.521740,Sinobambusa_spathiflorus_var.crassinodus:56.521740,Sinobambusa_dushanensis:56.521740,Sinobambusa_rubroligula:56.521740,Sinobambusa_tootsik_var.dentata:56.521740,Sinobambusa_henryi:56.521740,Sinobambusa_intermedia:56.521740,Sinobambusa_tootsik_var.laeta:56.521740,Sinobambusa_nephroaurita:56.521740,Sinobambusa_tootsik:56.521740,Sinobambusa_spathiflorus:56.521740,Sinobambusa_yixingensis:56.521740,Sinobambusa_humilis:56.521740)Sinobambusa:56.521740,((((((((Pseudosasa_viridula:12.560387,Pseudosasa_amabilis:12.560387,Pseudosasa_longiligula:12.560387,Pseudosasa_hindsii:12.560387,Pseudosasa_maculifera:12.560387,Pseudosasa_acutivagina:12.560387,Pseudosasa_subsolida:12.560387,Pseudosasa_aeria:12.560387,Pseudosasa_pubiflora:12.560387,Pseudosasa_orthotropa:12.560387,Pseudosasa_japonica:12.560387,Pseudosasa_cantori:12.560387,Pseudosasa_wuyiensis:12.560387,Pseudosasa_guangxianensis:12.560387,Pseudosasa_yuelushanensis:12.560387)pseudosasa:12.560387):12.560387):12.560387):12.560387)bep:12.560387):12.560387):12.560387):12.560387,(Pseudostachyum_polymorphum:56.521740)Pseudostachyum:56.521740,(Dendrocalamus_latiflorus:56.521740,Dendrocalamus_asper:56.521740,Dendrocalamus_barbatus_var.internodiiradicatus:56.521740,Dendrocalamus_calostachyus:56.521740,Dendrocalamus_birmanicus:56.521740,Dendrocalamus_sinicus:56.521740,Dendrocalamus_tibeticus:56.521740,Dendrocalamus_sikkimensis:56.521740,Dendrocalamus_barbatus:56.521740,Dendrocalamus_semiscandens:56.521740,Dendrocalamus_bambusoides:56.521740,Dendrocalamus_parishii:56.521740,Dendrocalamus_hamiltonii:56.521740,Dendrocalamus_brandisii:56.521740,Dendrocalamus_pachystachys:56.521740,Dendrocalamus_farinosus:56.521740,Dendrocalamus_minor:56.521740,Dendrocalamus_pulverulentus:56.521740,Dendrocalamus_fugongensis:56.521740,Dendrocalamus_minor_var.amoenus:56.521740,Dendrocalamus_membranaceus:56.521740,Dendrocalamus_jianshuiensis:56.521740,Dendrocalamus_peculiaris:56.521740,Dendrocalamus_giganteus:56.521740)Dendrocalamus:56.521740,(Cephalostachyum_fuchsianum:56.521740,Cephalostachyum_pergracile:56.521740,Cephalostachyum_pallidum:56.521740)Cephalostachyum:56.521740,(Yushania_glauca:56.521740,Yushania_baishanzuensis:56.521740,Yushania_maculata:56.521740,Yushania_punctulata:56.52174,Yushania_crispata:56.52174,Yushania_andropogonoides:56.52174,Yushania_crassicollis:56.52174,Yushania_pachyclada:56.52174,Yushania_uniramosa:56.52174,Yushania_collina:56.52174,Yushania_farcticaulis:56.52174,Yushania_brevipaniculata:56.52174,Yushania_complanata:56.52174,Yushania_falcatiaurita:56.52174,Yushania_menghaiensis:56.52174,Yushania_levigata:56.52174,Yushania_qiaojiaensis:56.52174,Yushania_farinosa:56.52174,Yushania_polytricha:56.52174,Yushania_canoviridis:56.52174,Yushania_bojieiana:56.52174,Yushania_cava:56.52174,Yushania_grammata:56.52174,Yushania_laetevirens:56.52174,Yushania_varians:56.52174,Yushania_qiaojiaensis_var.nuda:56.52174,Yushania_brevis:56.52174,Yushania_mabianensis:56.52174,Yushania_oblonga:56.52174,Yushania_hirticaulis:56.52174,Yushania_basihirsuta:56.52174,Yushania_longiuscula:56.52174,Yushania_exilis:56.52174,Yushania_mitis:56.52174,Yushania_chingii:56.52174,Yushania_lineolata:56.52174,Yushania_lacera:56.52174,Yushania_elevata:56.52174,Yushania_flexa:56.52174,Yushania_wuyishanensis:56.52174,Yushania_xizangensis:56.52174,Yushania_auctiaurita:56.52174,Yushania_yadongensis:56.52174,Yushania_glandulosa:56.52174,Yushania_cartilaginea:56.52174,Yushania_niitakayamensis:56.52174,Yushania_longiaurita:56.52174,Yushania_rugosa:56.52174,Yushania_weixiensis:56.52174)Yushania:56.521740,(Leptaspis_formosana:56.521740)Leptaspis:56.521740,(Arundinaria_fargesii:56.521740,Arundinaria_qingchengshanensis:56.521740,Arundinaria_faberi:56.521740)Arundinaria:56.521740,(Pleioblastus_maculatus:56.521740,Pleioblastus_amarus_var.pendulifolius:56.521740,Pleioblastus_altiligulatus:56.521740,Pleioblastus_hsienchuensis_var.subglabratus:56.521740,Pleioblastus_amarus_var.hangzhouensis:56.521740,Pleioblastus_intermedius:56.521740,Pleioblastus_truncatus:56.521740,Pleioblastus_amarus:56.521740,Pleioblastus_maculosoides:56.521740,Pleioblastus_incarnatus:56.521740,Pleioblastus_amarus_var.tubatus:56.521740,Pleioblastus_juxianensis:56.521740,Pleioblastus_sanmingensis:56.521740,Pleioblastus_solidus:56.521740,Pleioblastus_wuyishanensis:56.521740,Pleioblastus_hsienchuensis:56.521740,Pleioblastus_yixingensis:56.521740,Pleioblastus_oleosus:56.521740,Pleioblastus_rugatus:56.521740)Pleioblastus:56.521740,(Schizostachyum_chinense:56.521740,Schizostachyum_dumetorum_var.xinwuense:56.521740,Schizostachyum_jaculans:56.521740,Schizostachyum_dumetorum:56.521740,Schizostachyum_funghomii:56.521740,Schizostachyum_diffusum:56.521740,Schizostachyum_hainanense:56.521740,Schizostachyum_pseudolima:56.521740)Schizostachyum:56.521740,(Semiarundinaria_fastuosa:56.521740)Semiarundinaria:56.521740,(Oligostachyum_scabriflorum:56.521740,Oligostachyum_spongiosum:56.521740,Oligostachyum_scabriflorum_var.breviligulatum:56.521740,Oligostachyum_puberulum:56.521740,Oligostachyum_hupehense:56.521740,Oligostachyum_nuspiculum:56.521740,Oligostachyum_scopulum:56.521740,Oligostachyum_glabrescens:56.521740,Oligostachyum_sulcatum:56.521740,Oligostachyum_lubricum:56.521740,Oligostachyum_gracilipes:56.521740,Oligostachyum_shiuyingianum:56.521740,Oligostachyum_paniculatum:56.521740,Oligostachyum_lanceolatum:56.521740,Oligostachyum_oedogonatum:56.521740)Oligostachyum:56.521740,(Shibataea_strigosa:56.521740,Shibataea_chinensis:56.521740,Shibataea_nanpingensis_var.fujianica:56.521740,Shibataea_chiangshanensis:56.521740,Shibataea_hispida:56.521740,Shibataea_nanpingensis:56.521740,Shibataea_kumasasa:56.521740,Shibataea_lanceifolia:56.521740)Shibataea:56.521740,(Gelidocalamus_tessellatus:56.521740,Gelidocalamus_multifolius:56.521740,Gelidocalamus_rutilans:56.521740,Gelidocalamus_longiinternodus:56.521740,Gelidocalamus_stellatus:56.521740,Gelidocalamus_annulatus:56.521740,Gelidocalamus_solidus:56.521740,Gelidocalamus_kunishii:56.521740,Gelidocalamus_latifolius:56.521740)Gelidocalamus:56.521740)poaceae:12.560387):12.560379):12.560394):12.560379):12.560394):12.560379)poales:12.560394,(((Areca_catechu:50.241547,Areca_triandra:50.241547)Areca:50.241547,(Chuniophoenix_nana:50.241547,Chuniophoenix_hainanensis:50.241547)Chuniophoenix:50.241547,(Phoenix_hanceana:50.241547,Phoenix_roebelenii:50.241547)Phoenix:50.241547,(Salacca_secunda:50.241547)Salacca:50.241547,(Livistona_saribus:50.241547,Livistona_chinensis:50.241547)Livistona:50.241547,(Nypa_fruticans:50.241547)Nypa:50.241547,(Trachycarpus_princeps:50.241547,Trachycarpus_nana:50.241547,Trachycarpus_fortunei:50.241547)Trachycarpus:50.241547,(Caryota_monostachya:50.241547,Caryota_urens:50.241547,Caryota_mitis:50.241547,Caryota_ochlandra:50.241547)Caryota:50.241547,(Wallichia_caryotoides:50.241547,Wallichia_chinensis:50.241547)Wallichia:50.241547,(Guihaia_grossefibrosa:50.241547,Guihaia_argyrata:50.241547)Guihaia:50.241547,(Rhapis_humilis:50.241547,Rhapis_robusta:50.241547,Rhapis_multifida:50.241547,Rhapis_gracilis:50.241547)Rhapis:50.241547,(Arenga_westerhoutii:50.241547,Arenga_pinnata:50.241547,Arenga_engleri:50.241547,Arenga_caudata:50.241547,Arenga_micrantha:50.241547,Arenga_longicarpa:50.241547)Arenga:50.241547,(Licuala_spinosa:50.241547,Licuala_dasyantha:50.241547,Licuala_fordiana:50.241547)Licuala:50.241547,(Cocos_nucifera:50.241547)Cocos:50.241547,(Pinanga_discolor:50.241547,Pinanga_chinensis:50.241547,Pinanga_tashiroi:50.241547,Pinanga_hexasticha:50.241547,Pinanga_viridis:50.241547,Pinanga_gracilis:50.241547,Pinanga_sinii:50.241547,Pinanga_macroclada:50.241547)Pinanga:50.241547,(Plectocomia_assamica:50.241547,Plectocomia_himalayana:50.241547,Plectocomia_kerrana:50.241547,Plectocomia_microstachys:50.241547)Plectocomia:50.241547,(Daemonorops_margaritae:50.241547)Daemonorops:50.241547,(Calamus_tetradactylus:50.241547,Calamus_giganteus_var.robustus:50.241547,Calamus_faberii:50.241547BerberisBerberis,Calamus_macrorrhynchus:50.241547,Calamus_wailong:50.241547,Calamus_simplicifolius:50.241547,Calamus_obovoideus:50.241547,Calamus_henryanus:50.241547,Calamus_dianbaiensis:50.241547,Calamus_egregius:50.241547,Calamus_compsostachys:50.241547,Calamus_tetradactyloides:50.241547,Calamus_walkerii:50.241547,Calamus_bonianus:50.241547,Calamus_hoplites:50.241547,Calamus_guangxiensis:50.241547,Calamus_austro-guangxiensis:50.241547,Calamus_balansaeanus_var.castaneolepis:50.241547,Calamus_rhabdocladus:50.241547,Calamus_oxycarpus:50.241547,Calamus_platyacanthus:50.241547,Calamus_pulchellus:50.241547,Calamus_siphonospathus_var.sublaevis:50.241547,Calamus_multispicatus:50.241547,Calamus_thysanolepis:50.241547,Calamus_karinensis:50.241547,Calamus_viminalis_var.fasciculatus:50.241547,Calamus_feanus_var.medogensis:50.241547,Calamus_nambariensis:50.241547,Calamus_yunnanensis_var.intermedius:50.241547,Calamus_distichus_var.shangsiensis:50.241547,Calamus_formosanus:50.241547,Calamus_quiquesetinervius:50.241547,Calamus_balansaeanus:50.241547,Calamus_gracilis:50.241547,Calamus_yangchunensis:50.241547,Calamus_melanochrous:50.241547,Calamus_yunnanensis:50.241547,Calamus_palustris:50.241547,Calamus_flagellum:50.241547,Calamus_erectus:50.241547)Calamus:50.241547)arecaceae:50.241547)arecales:50.241547)commelinids:12.560379,((((((((((Dracaena_terniflora:19.411507,Dracaena_hokouensis:19.411507,Dracaena_cambodiana:19.411507,Dracaena_cochinchinensis:19.411507,Dracaena_gracilis:19.411507,Dracaena_angustifolia:19.411507)Dracaena:19.411507)asparagaceae:19.411503):19.411510):19.411507):19.411499):19.411507):19.411514):19.411499):19.411514)asparagales:19.411499):12.560394,((((((Heterosmilax_septemnervia:32.298138,Heterosmilax_polyandra:32.298138,Heterosmilax_chinensis:32.298138,Heterosmilax_seisuiensis:32.298138,Heterosmilax_japonica:32.298138)Heterosmilax:32.298138,(Smilax_lanceifolia_var.opaca:32.298138,Smilax_lanceifolia_var.impressinervia:32.298138,Smilax_bapouensis:32.298138,Smilax_china:32.298138,Smilax_retroflexa:32.298138,Smilax_pachysandroides:32.298138,Smilax_perfoliata:32.298138,Smilax_horridiramula:32.298138,Smilax_lebrunii:32.298138,Smilax_megacarpa:32.298138,Smilax_megalantha:32.298138,Smilax_scobinicaulis:32.298138,Smilax_emeiensis:32.298138,Smilax_vanchingshanensis:32.298138,Smilax_quadrata:32.298138,Smilax_hypoglauca:32.298138,Smilax_fooningensis:32.298138,Smilax_corbularia_var.woodii:32.298138,Smilax_cyclophylla:32.298138,Smilax_glaucochina:32.298138,Smilax_nigrescens:32.298138,Smilax_guiyangensis:32.298138,Smilax_sieboldii:32.298138,Smilax_astrosperma:32.298138,Smilax_arisanensis:32.298138,Smilax_jiankunii:32.298138,Smilax_munita:32.298138,Smilax_corbularia:32.298138,Smilax_hayatae:32.298138,Smilax_lushuiensis:32.298138,Smilax_ovalifolia:32.298138,Smilax_luei:32.298138,Smilax_malipoensis:32.298138,Smilax_lanceifolia:32.298138,Smilax_lunglingensis:32.298138,Smilax_densibarbata:32.298138,Smilax_setiramula:32.298138,Smilax_chapaensis:32.298138,Smilax_griffithii:32.298138,Smilax_nantoensis:32.298138,Smilax_pinfaensis:32.298138,Smilax_darrisii:32.298138,Smilax_tsinchengshanensis:32.298138,Smilax_chingii:32.298138,Smilax_basilata:32.298138,Smilax_hemsleyana:32.298138,Smilax_gagnepainii:32.298138,Smilax_elegantissima:32.298138,Smilax_aspera:32.298138,Smilax_elongatoumbellata:32.298138,Smilax_synandra:32.298138,Smilax_glabra:32.298138,Smilax_discotis:32.298138,Smilax_aberrans:32.298138,Smilax_myrtillus:32.298138,Smilax_mairei:32.298138,Smilax_nervomarginata_var.liukiuensis:32.298138,Smilax_outanscianensis:32.298138,Smilax_elegans:32.298138,Smilax_bockii:32.298138,Smilax_kwangsiensis_var.setulosa:32.298138,Smilax_davidiana:32.298138,Smilax_microphylla:32.298138,Smilax_cocculoides:32.298138,Smilax_aspericaulis:32.298138,Smilax_bauhinioides:32.298138,Smilax_bracteata:32.298138,Smilax_nervomarginata:32.298138,Smilax_kwangsiensis:32.298138,Smilax_longebracteolata:32.298138,Smilax_lanceifolia_var.lanceolata:32.298138,Smilax_lanceifolia_var.elongata:32.298138,Smilax_austrozhejiangensis:32.298138)Smilax:32.298138)smilacaceae:32.298134):32.298141):32.298141):32.298126)liliales:32.298141):12.560379,((((((Freycinetia_formosana:34.092476)Freycinetia:34.092476,(Pandanus_urophyllus:34.092476,Pandanus_forceps:34.092476,Pandanus_tectorius:34.092476)Pandanus:34.092476)pandanaceae:34.092476):34.092476):34.092484)pandanales:34.092468):34.092484):12.560394):12.560394,(((Pothoidium_lobbianum:65.942032)Pothoidium:65.942032)araceae:65.942032)alismatales:65.942032):12.560364)monocots:12.560394,((((((((((((((((((Gironniera_subaequalis:51.003551)Gironniera:51.003551,(Aphananthe_cuspidata:51.003551)Aphananthe:51.003551,(Trema_cannabina:51.003551,Trema_tomentosa:51.003551,Trema_cannabina_var.dielsiana:51.003551,Trema_angustifolia:51.003551,Trema_orientalis:51.003551,Trema_nitida:51.003551,Trema_levigata:51.003551)Trema:51.003551,(Celtis_philippensis:51.003551,Celtis_timorensis:51.003551,Celtis_philippensis_var.consimilis:51.003551)Celtis:51.003551,(Ulmus_lanceaefolia:51.003551)Ulmus:51.003551)ulmaceae:51.003548,((((Streblus_ilicifolius:57.378994,Streblus_indicus:57.378994,Streblus_tonkinensis:57.378994,Streblus_asper:57.378994,Streblus_zeylanicus:57.378994,Streblus_taxoides:57.378994)Streblus:57.378994,((((Ficus_pumila_var.awkeotsang:38.252663,Ficus_sarmentosa_var.nipponica:38.252663,Ficus_vasculosa:38.252663,Ficus_subincisa:38.252663,Ficus_beipeiensis:38.252663,Ficus_pumila:38.252663,Ficus_variolosa:38.252663,Ficus_tannoensis:38.252663,Ficus_undulata:38.252663,Ficus_pyriformis:38.252663,Ficus_cumingii:38.252663,Ficus_irisana:38.252663,Ficus_aurantiaca:38.252663,Ficus_benjamina:38.252663,Ficus_benjamina_var.nuda:38.252663,Ficus_hirta:38.252663,Ficus_pubigera_var.maliformis:38.252663,Ficus_auriculata:38.252663,Ficus_sarmentosa_var.duclouxii:38.252663,Ficus_daimingshanensis:38.252663,Ficus_hookeriana:38.252663,Ficus_glaberrima:38.252663,Ficus_virgata:38.252663,Ficus_tikoua:38.252663,Ficus_dinganensis:38.252663,Ficus_pisocarpa:38.252663,Ficus_hispida:38.252663,Ficus_trichocarpa_var.obtusa:38.252663,Ficus_curtipes:38.252663,Ficus_polynervis:38.252663,Ficus_ampelas:38.252663,Ficus_fusuiensis:38.252663,Ficus_altissima:38.252663,Ficus_praetermissa:38.252663,Ficus_gasparriniana:38.252663,Ficus_laevis:38.252663,Ficus_trivia_var.laevigata:38.252663,Ficus_guangxiensis:38.252663,Ficus_guizhouensis:38.252663,Ficus_pubigera:38.252663,Ficus_ischnopoda:38.252663,Ficus_annulata:38.252663,Ficus_benguetensis:38.252663,Ficus_esquiroliana:38.252663,Ficus_semicordata:38.252663,Ficus_simplicissima:38.252663,Ficus_subulata:38.252663,Ficus_henryi:38.252663,Ficus_fulva:38.252663,Ficus_stricta:38.252663,Ficus_nervosa:38.252663,Ficus_racemosa:38.252663,Ficus_septica:38.252663,Ficus_tinctoria:38.252663,Ficus_pubigera_var.anserina:38.252663,Ficus_gasparriniana_var.laceratifolia:38.252663,Ficus_maclellandi_var.maclellandi:38.252663,Ficus_cardiophylla:38.252663,Ficus_ovatifolia:38.252663,Ficus_pubinervis:38.252663,Ficus_pedunculosa:38.252663,Ficus_drupacea_var.pubescens:38.252663,Ficus_napoensis:38.252663,Ficus_sarmentosa_var.impressa:38.252663,Ficus_tuphapensis:38.252663,Ficus_prostrata:38.252663,Ficus_oligodon:38.252663,Ficus_tinctoria_subsp.swinhoei:38.252663,Ficus_sarmentosa:38.252663,Ficus_religiosa:38.252663,Ficus_pandurata:38.252663,Ficus_langkokensis:38.252663,Ficus_pubilimba:38.252663,Ficus_geniculata:38.252663,Ficus_microcarpa:38.252663,Ficus_racemosa_var.miquelli:38.252663,Ficus_squamosa:38.252663,Ficus_ruyuanensis:38.252663,Ficus_neriifolia:38.252663,Ficus_chapaensis:38.252663,Ficus_heterophylla:38.252663,Ficus_abelii:38.252663,Ficus_fistulosa:38.252663,Ficus_formosana:38.252663,Ficus_hederacea:38.252663,Ficus_cyrtophylla:38.252663,Ficus_pubigera_var.reticulata:38.252663,Ficus_sarmentosa_var.lacrymans:38.252663,Ficus_heteropleura:38.252663,Ficus_chartacea_var.torulosa:38.252663,Ficus_filicauda:38.252663,Ficus_trivia:38.252663,Ficus_tinctoria_subsp.gibbosa:38.252663,Ficus_rumphii:38.252663,Ficus_concinna:38.252663,Ficus_tsiangii:38.252663,Ficus_sagittata:38.252663,Ficus_elastica:38.252663,Ficus_callosa:38.252663,Ficus_sinociliata:38.252663,Ficus_vaccinioides:38.252663,Ficus_yunnanensis:38.252663,Ficus_variegata:38.252663,Ficus_sarmentosa_var.luducca:38.252663,Ficus_filicauda_var.longipes:38.252663,Ficus_gasparriniana_var.esquirolii:38.252663,Ficus_sarmentosa_var.henryi:38.252663,Ficus_drupacea:38.252663,Ficus_orthoneura:38.252663,Ficus_chartacea:38.252663,Ficus_stenophylla:38.252663)ficus:38.252663,(((((antiaris_toxicaria:12.750887)antiaris:12.750887):12.750889):12.750885):12.750889):12.750889):12.750885):12.750885):12.750893,(Cudrania_cochinchinensis:57.378994,Cudrania_amboinensis:57.378994,Cudrania_pubescens:57.378994)Cudrania:57.378994,((((Artocarpus_hypargyreus:39.345596,Artocarpus_petelotii:39.345596,Artocarpus_styracifolius:39.345596,Artocarpus_gongshanensis:39.345596,Artocarpus_nitidus_subsp.lingnanensis:39.345596,Artocarpus_pithecogallus:39.345596,Artocarpus_xanthocarpus:39.345596,Artocarpus_nanchuanensis:39.345596,Artocarpus_nitidus_subsp.griffithii:39.345596,Artocarpus_tonkinensis:39.345596,Artocarpus_lacucha:39.345596,(artocarpus_heterophyllus:19.672798):19.672798)artocarpus:19.672798):19.672798):19.672798,(((((Morus_cathayana_var.gongshanensis:16.393999)morus:16.393999):16.393997):16.394001):16.393997):16.393997):16.393997,(Malaisia_scandens:57.378994)Malaisia:57.378994)moraceae:12.750885,((Sarcochlamys_pulcherrima:42.502956)Sarcochlamys:42.502956,(Maoutia_puya:42.502956)Maoutia:42.502956,(Dendrocnide_stimulans:42.502956,Dendrocnide_sinuata:42.502956,Dendrocnide_meyeniana_f.meyeniana:42.502956)Dendrocnide:42.502956,(Leucosyke_quadrinervia:42.502956)Leucosyke:42.502956,(Pipturus_arborescens:42.502956)Pipturus:42.502956,(Poikilospermum_lanceolatum:42.502956)Poikilospermum:42.502956,(Oreocnide_obovata_var.obovata:42.502956,Oreocnide_frutescens_subsp.occidentalis:42.502956,Oreocnide_rubescens:42.502956,Oreocnide_tonkinensis_var.tonkinensis:42.502956,Oreocnide_integrifolia_subsp.integrifolia:42.502956,Oreocnide_serrulata:42.502956,Oreocnide_pedunculata:42.502956,Oreocnide_frutescens:42.502956)Oreocnide:42.502956)urticaceae:42.502960):12.750893):12.750885):12.750885,(((((Ventilago_inaequilateralis:46.044872,Ventilago_oblongifolia:46.044872,Ventilago_calyculata:46.044872,Ventilago_leiocarpa_var.pubescens:46.044872,Ventilago_calyculata_var.trichoclada:46.044872,Ventilago_elegans:46.044872,Ventilago_leiocarpa:46.044872)ventilago:46.044872,((((Rhamnella_rubrinervis:18.417948)rhamnella:18.417948)rhamneae:18.417950,(Sageretia_lucida:27.626923,Sageretia_randaiensis:27.626923,Sageretia_thea_var.tomentosa:27.626923,Sageretia_pedicellata:27.626923,Sageretia_thea:27.626923,Sageretia_paucicostata:27.626923,Sageretia_laxiflora:27.626923,Sageretia_yilinii:27.626923,Sageretia_subcaudata:27.626923,Sageretia_gracilis:27.626923,Sageretia_thea_var.cordiformis:27.626923,Sageretia_rugosa:27.626923,Sageretia_horrida:27.626923,Sageretia_melliana:27.626923,Sageretia_omeiensis:27.626923,Sageretia_hamosa:27.626923,Sageretia_lijiangensis:27.626923)sageretia:27.626923,((Rhamnus_hemsleyana_var.yunnanensis:18.417948,Rhamnus_hemsleyana:18.417948,Rhamnus_xizangensis:18.417948,Rhamnus_bodinieri:18.417948)rhamnus:18.417948):18.417950,(Scutia_eberhardtii:27.626923)scutia:27.626923):18.417946):18.417953)rhamnoids:18.417946,(((((Ziziphus_mauritiana:18.417948,Ziziphus_rugosa:18.417948)ziziphus:18.417948,(Paliurus_orientalis:18.417948,Paliurus_hemsleyanus:18.417948,Paliurus_hirsutus:18.417948)paliurus:18.417948):18.417950)paliureae:18.417946,(Alphitonia_philippinensis:36.835896)alphitonia:36.835896,(Colubrina_pubescens:36.835896,Colubrina_asiatica:36.835896)colubrina:36.835896)ziziphoids:18.417953):18.417946,(Gouania_leptostachya_var.macrocarpa:55.253845,Gouania_leptostachya_var.leptostachya:55.253845,Gouania_javanica:55.253845,Gouania_leptostachya_var.tonkinensis:55.253845)Gouana:55.253845)rhamnaceae:18.417953,((Elaeagnus_thunbergii:42.975216,Elaeagnus_difficilis:42.975216,Elaeagnus_pallidiflora:42.975216,Elaeagnus_macrantha:42.975216,Elaeagnus_taliensis:42.975216,Elaeagnus_difficilis_var.brevistyla:42.975216,Elaeagnus_oldhami:42.975216,Elaeagnus_tubiflora:42.975216,Elaeagnus_pungens:42.975216,Elaeagnus_loureirii:42.975216,Elaeagnus_gonyanthes:42.975216,Elaeagnus_retrostyla:42.975216,Elaeagnus_lanpingensis:42.975216,Elaeagnus_liuzhouensis:42.975216,Elaeagnus_luxiensis:42.975216,Elaeagnus_luoxiangensis:42.975216,Elaeagnus_viridis:42.975216,Elaeagnus_glabra:42.975216,Elaeagnus_pilostyla:42.975216,Elaeagnus_conferta_var.conferta:42.975216,Elaeagnus_bockii_var.muliensis:42.975216,Elaeagnus_obovatifolia:42.975216,Elaeagnus_sarmentosa:42.975216,Elaeagnus_lanceolata:42.975216,Elaeagnus_pingnanensis:42.975216,Elaeagnus_calcarea:42.975216,Elaeagnus_formosana:42.975216,Elaeagnus_wenshanensis:42.975216,Elaeagnus_xizangensis:42.975216,Elaeagnus_xichouensis:42.975216,Elaeagnus_geniculata:42.975216,Elaeagnus_tutcheri:42.975216,Elaeagnus_schlechtendalii:42.975216,Elaeagnus_xingwenensi:42.975216,Elaeagnus_henryi:42.975216,Elaeagnus_heterophylla:42.975216,Elaeagnus_tonkinensis:42.975216,Elaeagnus_yunnanensis:42.975216,Elaeagnus_cinnamomifolia:42.975216,Elaeagnus_delavayi:42.975216,Elaeagnus_longiloba:42.975216,Elaeagnus_bockii_var.bockii:42.975216,Elaeagnus_bambusetorum:42.975216)Elaeagnus:42.975216)elaeagnaceae:42.975212):18.417938):18.417953):12.750885,(((((((Pygeum_macrocarpum:34.127377,Pygeum_laxiflorum:34.127377,Pygeum_topengii:34.127377,Pygeum_henryi:34.127377,Pygeum_oblongum:34.127377)pygeum:34.127377)amygdaleae:34.127373):34.127380,((((((((Pyracantha_fortuneana:31.502192,Pyracantha_inermis:31.502192,Pyracantha_densiflora:31.502192,Pyracantha_atalantioides:31.502192,Pyracantha_koidzumii:31.502192,Pyracantha_crenulata_var.kansuensis:31.502192,Pyracantha_crenulata:31.502192,Pyracantha_angustifolia:31.502192)pyracantha:31.502192,((Stranvaesia_davidiana_var.undulata:26.251827,Stranvaesia_oblanceolata:26.251827,Stranvaesia_davidiana:26.251827,Stranvaesia_amphidoxa_var.amphileia:26.251827,Stranvaesia_amphidoxa_var.amphidoxa:26.251827,Stranvaesia_tomentosa:26.251827,Stranvaesia_nussia:26.251827)stranvaesia:26.251827,((((Rhaphiolepis_ferruginea_var.serrata:10.500731,Rhaphiolepis_major:10.500731,Rhaphiolepis_umbellata:10.500731,Rhaphiolepis_salicifolia:10.500731,Rhaphiolepis_integerrima:10.500731,Rhaphiolepis_indica:10.500731,Rhaphiolepis_lanceolata:10.500731,Rhaphiolepis_ferruginea:10.500731)rhaphiolepis:10.500731,(Eriobotrya_serrata:10.500731,Eriobotrya_cavaleriei:10.500731,Eriobotrya_obovata:10.500731,Eriobotrya_prinoides:10.500731,Eriobotrya_malipoensis:10.500731,Eriobotrya_salwinensis:10.500731,Eriobotrya_japonica:10.500731,Eriobotrya_deflexa:10.500731,Eriobotrya_tengyuehensis:10.500731,Eriobotrya_elliptica:10.500731,Eriobotrya_fragrans:10.500731,Eriobotrya_seguinii:10.500731,Eriobotrya_bengalensis_f.angustifolia:10.500731,Eriobotrya_henryi:10.500731)eriobotrya:10.500731):10.500731):10.500731):10.500732):10.500729,(((Photinia_prionophylla:15.751096,Photinia_megaphylla:15.751096,Photinia_loriformis:15.751096,Photinia_benthamiana_var.obovata:15.751096,Photinia_lasiogyna:15.751096,Photinia_tushanensis:15.751096,Photinia_tsaii:15.751096,Photinia_glabra:15.751096,Photinia_kwangsiensis:15.751096,Photinia_bodinieri:15.751096,Photinia_callosa:15.751096,Photinia_crassifolia:15.751096,Photinia_bergerae:15.751096,Photinia_chihsiniana:15.751096,Photinia_benthamiana_var.salicifolia:15.751096,Photinia_lochengensis:15.751096,Photinia_podocarpifolia:15.751096,Photinia_arguta_var.hookeri:15.751096,Photinia_lanuginosa:15.751096,Photinia_benthamiana:15.751096,Photinia_glomerata:15.751096,Photinia_integrifolia:15.751096,Photinia_raupingensis:15.751096,Photinia_serrulata:15.751096,Photinia_prunifolia:15.751096,Photinia_beckii:15.751096,Photinia_impressivena:15.751096,Photinia_berberidifolia:15.751096,Photinia_chingiana:15.751096,Photinia_stenophylla:15.751096,Photinia_zhejiangensis:15.751096,Photinia_prunifolia_var.denticulata:15.751096)photinia:15.751096):15.751097):15.751095,((Cotoneaster_buxifolius:21.001461,Cotoneaster_sherriffii:21.001461,Cotoneaster_salicifolius:21.001461,Cotoneaster_rhytidophyllus:21.001461,Cotoneaster_harrovianus:21.001461,Cotoneaster_morrisonensis:21.001461,Cotoneaster_turbinatus:21.001461,Cotoneaster_microphyllus_var.glacialis:21.001461,Cotoneaster_franchetii:21.001461,Cotoneaster_microphyllus_var.thymifolius:21.001461,Cotoneaster_glaucophyllus_var.meiophyllus:21.001461,Cotoneaster_rubens_var.minianus:21.001461,Cotoneaster_dielsianus_var.elegans:21.001461,Cotoneaster_microphyllus:21.001461,Cotoneaster_rotundifolius:21.001461,Cotoneaster_salicifolius_var.angustus:21.001461,Cotoneaster_pannosus:21.001461,Cotoneaster_dammerii_var.radicans:21.001461,Cotoneaster_salicifolius_var.rugosus:21.001461,Cotoneaster_dammerii:21.001461,Cotoneaster_microphyllus_var.cochleatus:21.001461,Cotoneaster_wardii:21.001461,Cotoneaster_conspicuus:21.001461,Cotoneaster_salicifolius_var.henryanus:21.001461,Cotoneaster_buxifolius_var.marginatus:21.001461,Cotoneaster_glaucophyllus:21.001461,Cotoneaster_glabratus:21.001461,Cotoneaster_coriaceus:21.001461)cotoneaster:21.001461):21.001461,(((Crataegus_chungtienensis:15.751096)crataegus:15.751096):15.751097):15.751095,((Osteomeles_anthyllidifolia:21.001461,Osteomeles_subrotunda:21.001461)osteomeles:21.001461):21.001461)pyrinae:10.500736):10.500725):10.500732)pyreae:10.500732)pyrodae:10.500732):10.500725):10.500740):10.500732)spiraeoideae:10.500717,((Dryas_octopetala_var.asiatica:52.503651)dryas:52.503651)dryadoideae:52.503654):10.500732,(Laurocerasus_spinulosa:84.005844,Laurocerasus_zippeliana:84.005844,Laurocerasus_aquifolioides:84.005844,Laurocerasus_fordiana:84.005844,Laurocerasus_undulata:84.005844,Laurocerasus_jenkinsii:84.005844,Laurocerasus_hypotricha:84.005844,Laurocerasus_menghaiensis:84.005844,Laurocerasus_australis:84.005844,Laurocerasus_marginata:84.005844,Laurocerasus_phaeosticta:84.005844,Laurocerasus_andersonii:84.005844,Laurocerasus_dolichophylla:84.005844)Laurocerasus:84.005844,(Docynia_delavayi:84.005844)Docynia:84.005844,(Dichotomanthus_tristaniaecarpa:84.005844)Dichotomanthus:84.005844,(Rosa_baiyushanensis:84.005844,Rosa_acicularis:84.005844,Rosa_odorata_var.gigantea:84.005844,Rosa_banksiae_var.normalis:84.005844,Rosa_odorata_var.erubescens:84.005844,Rosa_transmorrisonensis:84.005844,Rosa_kweichowensis:84.005844,Rosa_laevigata:84.005844,Rosa_odorata_var.pseudindica:84.005844,Rosa_lucidissima:84.005844,Rosa_banksiae:84.005844,Rosa_bracteata:84.005844,Rosa_murielae:84.005844,Rosa_albertii:84.005844,Rosa_odorata:84.005844,Rosa_anemoniflora:84.005844,Rosa_longicuspis:84.005844)Rosa:84.005844,(Rubus_jianensis:84.005844,Rubus_henryi_var.sozostylus:84.005844,Rubus_irenaeus:84.005844,Rubus_henryi:84.005844,Rubus_chroosepalus:84.005844,Rubus_pacificus:84.005844,Rubus_bambusarum:84.005844)Rubus:84.005844,(Connarus_yunnanensis:84.005844)Connarus:84.005844)rosaceae:10.500732)rosales:10.500732,(((((Quercus_monimotricha:39.377743,Quercus_engleriana:39.377743,Quercus_bawanglingensis:39.377743,Quercus_aquifolioides:39.377743,Quercus_spinosa:39.377743,Quercus_setulosa:39.377743,Quercus_semecarpifolia:39.377743,Quercus_senescens:39.377743,Quercus_oxyphylla:39.377743,Quercus_baronii_var.baronii:39.377743,Quercus_kingiana:39.377743,Quercus_marlipoensis:39.377743,Quercus_rehderiana:39.377743,Quercus_guyavaefolia:39.377743,Quercus_dolicholepis:39.377743,Quercus_tarokoensis:39.377743,Quercus_utilis:39.377743,Quercus_cocciferoides:39.377743,Quercus_lanata:39.377743,Quercus_phillyraeoides:39.377743,Quercus_lodicosa:39.377743,Quercus_acrodonta:39.377743,Quercus_franchetii:39.377743)Quercus:39.377743,(Lithocarpus_dealbatus:39.377743,Lithocarpus_craibianus:39.377743,Lithocarpus_leucodermis:39.377743,Lithocarpus_cleistocarpus:39.377743,Lithocarpus_tenuilimbus:39.377743,Lithocarpus_cryptocarpus:39.377743,Lithocarpus_areca:39.377743,Lithocarpus_longipedicellatus:39.377743,Lithocarpus_trachycarpus:39.377743,Lithocarpus_kawakamii:39.377743,Lithocarpus_fangii:39.377743,Lithocarpus_echinotholus:39.377743,Lithocarpus_damiaoshanicus:39.377743,Lithocarpus_megalophyllus:39.377743,Lithocarpus_paihengii:39.377743,Lithocarpus_pseudoreinwardtii:39.377743,Lithocarpus_pakhaensis:39.377743,Lithocarpus_brachystachyus:39.377743,Lithocarpus_cleistocarpus_var.omeiensis:39.377743,Lithocarpus_oblanceolatus:39.377743,Lithocarpus_grandifolius:39.377743,Lithocarpus_macilentus:39.377743,Lithocarpus_cucullatus:39.377743,Lithocarpus_harlandii:39.377743,Lithocarpus_gaoligongensis:39.377743,Lithocarpus_collettii:39.377743,Lithocarpus_nitidinux:39.377743,Lithocarpus_mairei:39.377743,Lithocarpus_irwinii:39.377743,Lithocarpus_lepidocarpus:39.377743,Lithocarpus_phansipanensis:39.377743,Lithocarpus_magneinii:39.377743,Lithocarpus_melanochromus:39.377743,Lithocarpus_fenzelianus:39.377743,Lithocarpus_carolinae:39.377743,Lithocarpus_balansae:39.377743,Lithocarpus_elizabethiae:39.377743,Lithocarpus_pachylepis:39.377743,Lithocarpus_pachyphyllus:39.377743,Lithocarpus_elaeagnifolius:39.377743,Lithocarpus_echinophorus:39.377743,Lithocarpus_tubulosus:39.377743,Lithocarpus_levis:39.377743,Lithocarpus_skanianus:39.377743,Lithocarpus_corneus_var.zonatus:39.377743,Lithocarpus_hypoglaucus:39.377743,Lithocarpus_henryi:39.377743,Lithocarpus_tephrocarpus:39.377743,Lithocarpus_pseudoxizangensis:39.377743,Lithocarpus_gymnocarpus:39.377743,Lithocarpus_attenuatus:39.377743,Lithocarpus_truncatus:39.377743,Lithocarpus_chrysocomus:39.377743,Lithocarpus_echinophorus_var.bidoupensis:39.377743,Lithocarpus_shinsuiensis:39.377743,Lithocarpus_floccosus:39.377743,Lithocarpus_glaber:39.377743,Lithocarpus_mekongensis:39.377743,Lithocarpus_oleaefolius:39.377743,Lithocarpus_laoticus:39.377743,Lithocarpus_triqueter:39.377743,Lithocarpus_howii:39.377743,Lithocarpus_silvicolarum:39.377743,Lithocarpus_quercifolius:39.377743,Lithocarpus_taitoensis:39.377743,Lithocarpus_brevicaudatus:39.377743,Lithocarpus_handelianus:39.377743,Lithocarpus_dodonaeifolius:39.377743,Lithocarpus_longanoides:39.377743,Lithocarpus_cinereus:39.377743,Lithocarpus_thomsonii:39.377743,Lithocarpus_obovatilimbus:39.377743,Lithocarpus_uvariifolius_var.ellipticus:39.377743,Lithocarpus_haipinii:39.377743,Lithocarpus_variolosus:39.377743,Lithocarpus_pseudovestitus:39.377743,Lithocarpus_rhabdostachyus_subsp.dakhaesis:39.377743,Lithocarpus_litseifolius_var.pubescens:39.377743,Lithocarpus_bonnetii:39.377743,Lithocarpus_calolepis:39.377743,Lithocarpus_calophyllus:39.377743,Lithocarpus_fohaiensis:39.377743,Lithocarpus_fordianus:39.377743,Lithocarpus_mianningensis:39.377743,Lithocarpus_obscurus:39.377743,Lithocarpus_xylocarpus:39.377743,Lithocarpus_litseifolius:39.377743,Lithocarpus_rosthornii:39.377743,Lithocarpus_nantoensis:39.377743,Lithocarpus_fenestratus:39.377743,Lithocarpus_eriobotryoides:39.377743,Lithocarpus_tabularis:39.377743,Lithocarpus_laetus:39.377743,Lithocarpus_qinzhouicus:39.377743,Lithocarpus_chiungchungensis:39.377743,Lithocarpus_sphaerocarpus:39.377743,Lithocarpus_bacgiangensis:39.377743,Lithocarpus_propinquus:39.377743,Lithocarpus_echinophorus_var.chapensis:39.377743,Lithocarpus_pasania:39.377743,Lithocarpus_talangensis:39.377743,Lithocarpus_iteaphyllus:39.377743,Lithocarpus_naiadarum:39.377743,Lithocarpus_pachyphyllus_var.fruticosus:39.377743,Lithocarpus_formosanus:39.377743,Lithocarpus_ithyphyllus:39.377743,Lithocarpus_elmerrillii:39.377743,Lithocarpus_garrettianus:39.377743,Lithocarpus_caudatilimbus:39.377743,Lithocarpus_xizangensis:39.377743,Lithocarpus_lycoperdon:39.377743,Lithocarpus_apricus:39.377743,Lithocarpus_microspermus:39.377743,Lithocarpus_arcuala:39.377743,Lithocarpus_petelotii:39.377743,Lithocarpus_amygdalifolius:39.377743,Lithocarpus_corneus:39.377743,Lithocarpus_farinulentus:39.377743,Lithocarpus_listeri:39.377743,Lithocarpus_jenkinsii:39.377743,Lithocarpus_hancei:39.377743,Lithocarpus_crassifolius:39.377743,Lithocarpus_yongfuensis:39.377743,Lithocarpus_konishii:39.377743,Lithocarpus_cyrtocarpus:39.377743,Lithocarpus_amoenus:39.377743,Lithocarpus_paniculatus:39.377743,Lithocarpus_chifui:39.377743,Lithocarpus_confinis:39.377743,Lithocarpus_corneus_var.angustifolius:39.377743,Lithocarpus_uvariifolius:39.377743)Lithocarpus:39.377743,(Cyclobalanopsis_albicaulis:39.377743,Cyclobalanopsis_tenuicupula:39.377743,Cyclobalanopsis_lamellosa:39.377743,Cyclobalanopsis_saravanensis:39.377743,Cyclobalanopsis_chapensis:39.377743,Cyclobalanopsis_bella:39.377743,Cyclobalanopsis_gilva:39.377743,Cyclobalanopsis_rex:39.377743,Cyclobalanopsis_daimingshanensis:39.377743,Cyclobalanopsis_jenseniana:39.377743,Cyclobalanopsis_obovatifolia:39.377743,Cyclobalanopsis_austroglauca:39.377743,Cyclobalanopsis_glaucoides:39.377743,Cyclobalanopsis_lobbii:39.377743,Cyclobalanopsis_tiaoloshanica:39.377743,Cyclobalanopsis_disciformis:39.377743,Cyclobalanopsis_dinghuensis:39.377743,Cyclobalanopsis_multiervis:39.377743,Cyclobalanopsis_camusae:39.377743,Cyclobalanopsis_fleuryi:39.377743,Cyclobalanopsis_chungii:39.377743,Cyclobalanopsis_kouangsiensis:39.377743,Cyclobalanopsis_argyrotricha:39.377743,Cyclobalanopsis_stewardiana_var.stewardiana:39.377743,Cyclobalanopsis_chevalieri:39.377743,Cyclobalanopsis_thorelii:39.377743,Cyclobalanopsis_edithiae:39.377743,Cyclobalanopsis_annulata:39.377743,Cyclobalanopsis_poilanei:39.377743,Cyclobalanopsis_delavayi:39.377743,Cyclobalanopsis_litoralis:39.377743,Cyclobalanopsis_jinpinensis:39.377743,Cyclobalanopsis_chingsiensis:39.377743,Cyclobalanopsis_hui:39.377743,Cyclobalanopsis_blakei:39.377743,Cyclobalanopsis_phanera:39.377743,Cyclobalanopsis_championii:39.377743,Cyclobalanopsis_lungmaiensis:39.377743,Cyclobalanopsis_subhinoides:39.377743,Cyclobalanopsis_oxyodon:39.377743,Cyclobalanopsis_pachyloma:39.377743,Cyclobalanopsis_tomentosinervis:39.377743,Cyclobalanopsis_gambleana:39.377743,Cyclobalanopsis_kerrii:39.377743,Cyclobalanopsis_helferiana:39.377743,Cyclobalanopsis_motuoensis:39.377743,Cyclobalanopsis_litseoides:39.377743,Cyclobalanopsis_ningangensis:39.377743,Cyclobalanopsis_glauca:39.377743,Cyclobalanopsis_kiukiangensis:39.377743,Cyclobalanopsis_hypophaea:39.377743,Cyclobalanopsis_delicatula:39.377743,Cyclobalanopsis_xanthotricha:39.377743,Cyclobalanopsis_morii:39.377743,Cyclobalanopsis_stenophylloides:39.377743,Cyclobalanopsis_elevaticostata:39.377743,Cyclobalanopsis_patelliformis:39.377743,Cyclobalanopsis_semiserratoides:39.377743,Cyclobalanopsis_pentacycla:39.377743,Cyclobalanopsis_sichourensis:39.377743,Cyclobalanopsis_gracilis:39.377743,Cyclobalanopsis_myrsinifolia:39.377743,Cyclobalanopsis_yingjiangensis:39.377743,Cyclobalanopsis_yonganensis:39.377743,Cyclobalanopsis_austro-cochinchinensis:39.377743,Cyclobalanopsis_sessilifolia:39.377743,Cyclobalanopsis_augustinii:39.377743,Cyclobalanopsis_longinux:39.377743,Cyclobalanopsis_bambusaefolia:39.377743)Cyclobalanopsis:39.377743,(Trigonobalanus_doichangensis:39.377743)Trigonobalanus:39.377743,(Castanopsis_tcheponensis:39.377743,Castanopsis_platyacantha:39.377743,Castanopsis_rufescens:39.377743,Castanopsis_undulatifolia:39.377743,Castanopsis_damingshanensis:39.377743,Castanopsis_megaphylla:39.377743,Castanopsis_kawakamii:39.377743,Castanopsis_carlesii_var.spinulosa:39.377743,Castanopsis_echinocarpa:39.377743,Castanopsis_calathiformis:39.377743,Castanopsis_delavayi:39.377743,Castanopsis_tonkinensis:39.377743,Castanopsis_tibetana:39.377743,Castanopsis_kweichowensis:39.377743,Castanopsis_hainanensis:39.377743,Castanopsis_nigrescens:39.377743,Castanopsis_rufotomentosa:39.377743,Castanopsis_hystrix:39.377743,Castanopsis_chunii:39.377743,Castanopsis_crassifolia:39.377743,Castanopsis_hupehensis:39.377743,Castanopsis_concinna:39.377743,Castanopsis_tribuloides:39.377743,Castanopsis_jianfenglingensis:39.377743,Castanopsis_oblonga:39.377743,Castanopsis_fargesii:39.377743,Castanopsis_sclerophylla:39.377743,Castanopsis_boisii:39.377743,Castanopsis_ledongensis:39.377743,Castanopsis_clarkei:39.377743,Castanopsis_fissa:39.377743,Castanopsis_uraiana:39.377743,Castanopsis_rockii:39.377743,Castanopsis_longzhouica:39.377743,Castanopsis_lamontii:39.377743,Castanopsis_fabri:39.377743,Castanopsis_cerebrina:39.377743,Castanopsis_fordii:39.377743,Castanopsis_mekongensis:39.377743,Castanopsis_carlesii:39.377743,Castanopsis_densispinosa:39.377743,Castanopsis_amabilis:39.377743,Castanopsis_ouonbiensis:39.377743,Castanopsis_remotidenticulata:39.377743,Castanopsis_ferox:39.377743,Castanopsis_eyrei:39.377743,Castanopsis_ceratacantha:39.377743,Castanopsis_wenchangensis:39.377743,Castanopsis_xichouensis:39.377743,Castanopsis_fleuryi:39.377743,Castanopsis_jucunda:39.377743,Castanopsis_argyrophylla:39.377743,Castanopsis_indica:39.377743,Castanopsis_orthacantha:39.377743,Castanopsis_globigemmata:39.377743,Castanopsis_choboensis:39.377743,Castanopsis_longispina:39.377743,Castanopsis_chinensis:39.377743,Castanopsis_tessellata:39.377743,Castanopsis_subuliformis:39.377743)Castanopsis:39.377743)fagaceae:39.377739,((((Myrica_esculenta:23.626644,Myrica_adenophora_var.adenophora:23.626644,Myrica_rubra:23.626644,Myrica_nana:23.626644)Myrica:23.626644)myricaceae:23.626640,((Engelhardtia_hainanensis:23.626642,Engelhardtia_roxburghiana:23.626642)Engelhardtia:23.626642)juglandaceae:23.626644):23.626648):23.626648):23.626633)fagales:23.626648,(((((Hodgsonia_heteroclita:27.564417)Hodgsonia:27.564417,(Zanonia_indica_var.pubescens:27.564417)Zanonia:27.564417)cucurbitaceae:27.564419):27.564415):27.564415)cucurbitales:27.564423):23.626648):10.500732,(((((((((((((((((((((Acacia_megaladena:20.193714,Acacia_pruinescens:20.193714,Acacia_caesia:20.193714,Acacia_confusa:20.193714,Acacia_teniana:20.193714,Acacia_yunnanensis:20.193714)acacia:20.193714,((Albizia_odoratissima:16.154972)albizia:16.154972,(((Archidendron_utile:8.077486,Archidendron_clypearia:8.077486,Archidendron_lucidum:8.077486,Pithecellobium_dulce:8.077486,Archidendron_ellipticum:8.077486,Archidendron_cordifolium:8.077486)pithecellobium:8.077486)pithecellobium_alliance:8.077486):8.077486)ingeae:8.077484):8.077488):8.077484,((Leucaena_leucocephala:18.847467)leucaena:18.847467)leucaenae_group:18.847466):8.077488):8.077484,(Entada_phaseoloides:36.348686,Entada_rheedii:36.348686)entada:36.348686):8.077484):8.077484):8.077492):8.077484,((Erythrophleum_fordii:35.002438)erythrophleum:35.002438)dimorphandra_group:35.002441)mimosoids:8.077484):8.077484):8.077492,(((((Caesalpinia_magnifoliolata:21.539963,Caesalpinia_mimosoides:21.539963,Caesalpinia_minax:21.539963,Caesalpinia_sinensis:21.539963,Caesalpinia_cucullata:21.539963,Caesalpinia_hymenocarpa:21.539963,Caesalpinia_digyna:21.539963)caesalpinia:21.539963):21.539963)caesalpinia_group:21.539963)pterogyne_group:21.539963):21.539963):8.077484):8.077484):8.077484,(((((((((((((((((((Uraria_sinensis:7.673611)uraria:7.673611):7.673612,((Phyllodium_elegans:7.673611,Phyllodium_pulchellum:7.673611,Phyllodium_longipes:7.673611,Phyllodium_kurzianum:7.673611)phyllodium:7.673611,(Dendrolobium_triangulare:7.673611,Dendrolobium_dispermum:7.673611,Dendrolobium_umbellatum:7.673611)dendrolobium:7.673611,(Tadehagi_triquetrum:7.673611,Tadehagi_pseudotriquetrum:7.673611)tadehagi:7.673611):7.673612):7.673611)desmodieae:7.673613,(Mucuna_birdwoodiana:19.184029,Mucuna_sempervirens:19.184029,Mucuna_macrocarpa:19.184029,Mucuna_macrobotrys:19.184029,Mucuna_championii:19.184029,Mucuna_hainanensis:19.184029,Mucuna_bracteata:19.184029,Mucuna_interrupta:19.184029,Mucuna_gigantea:19.184029,Mucuna_calophylla:19.184029,Mucuna_cyclocarpa:19.184029,Mucuna_lamellata:19.184029)mucuna:19.184029):7.673611):7.673611):7.673611,((((Dunbaria_circinalis:12.277778)dunbaria:12.277778,(Flemingia_procumbens:12.277778,Flemingia_macrophylla:12.277778,Flemingia_kweichowensis:12.277778,Flemingia_fluminalis:12.277778,Flemingia_latifolia:12.277778,Flemingia_mengpengensis:12.277778,Flemingia_chappar:12.277778,Flemingia_philippinensis:12.277778,Flemingia_strobilifera:12.277778,Flemingia_grahamiana:12.277778,Flemingia_lineata:12.277778,Flemingia_glutinosa:12.277778,Flemingia_wallichii:12.277778,Flemingia_stricta:12.277778,Flemingia_paniculata:12.277778,Flemingia_involucrata:12.277778)flemingia:12.277778,(Cajanus_crassus:12.277778,Cajanus_grandiflorus:12.277778,Cajanus_cajan:12.277778)cajanus:12.277778)cajaninae:12.277777):12.277779,((Spatholobus_discolor:16.370371,Spatholobus_uniauritus:16.370371,Spatholobus_gengmaensis:16.370371,Spatholobus_pulcher:16.370371,Spatholobus_suberectus:16.370371,Spatholobus_biauritus:16.370371,Spatholobus_varians:16.370371)spatholobus:16.370371):16.370369):12.277779):7.673611)phaseoloids:7.673615,(((((((Derris_alborubra:9.592014,Derris_marginata:9.592014,Derris_scabricaulis:9.592014,Derris_harrowiana:9.592014,Derris_tonkinensis_var.compacta:9.592014,Aganope_latifolia:9.592014,Aganope_dinghuensis:9.592014,Derris_tonkinensis:9.592014,Derris_breviramosa:9.592014,Paraderris_glauca:9.592014,Paraderris_hainanensis:9.592014,Paraderris_canarensis:9.592014,Derris_fordii_var.lucida:9.592014,Derris_eriocarpa:9.592014,Paraderris_elliptica:9.592014,Aganope_thyrsiflora:9.592014,Derris_cavaleriei:9.592014,Derris_laxiflora:9.592014,Derris_caudatilimba:9.592014,Derris_ferruginea:9.592014,Paraderris_malaccensis:9.592014,Derris_trifoliata:9.592014,Paraderris_hancei:9.592014,Derris_palmifolia:9.592014,Derris_fordii:9.592014)derris:9.592014):9.592014):9.592014):9.592014)millettioids:9.592014):9.592018):9.592010):7.673607):7.673615,((((((Antheroporum_glaucum:13.154762,Antheroporum_harmandii:13.154762)antheroporum:13.154762):13.154762):13.154762):13.154762)irlc:13.154762,(((Sesbania_grandiflora:19.732143)sesbania:19.732143):19.732143):19.732143):13.154762):7.673607):7.673615):7.673607,(((Ormosia_ferruginea:33.572048,Ormosia_saxatilis:33.572048,Ormosia_howii:33.572048,Ormosia_merrilliana:33.572048,Ormosia_yunnanensis:33.572048,Ormosia_balansae:33.572048,Ormosia_inflata:33.572048,Ormosia_purpureiflora:33.572048,Ormosia_emarginata:33.572048,Ormosia_pachycarpa_var.tenuis:33.572048,Ormosia_semicastrata_f.pallida:33.572048,Ormosia_striata:33.572048,Ormosia_simplicifolia:33.572048,Ormosia_fordiana:33.572048,Ormosia_glaberrima:33.572048,Ormosia_pinnata:33.572048,Ormosia_hekouensis:33.572048,Ormosia_hosiei:33.572048,Ormosia_elliptica:33.572048,Ormosia_henryi:33.572048,Ormosia_apiculata:33.572048,Ormosia_olivacea:33.572048,Ormosia_semicastrata_f.litchifolia:33.572048,Ormosia_sericeolucida:33.572048,Ormosia_pachyptera:33.572048,Ormosia_xylocarpa:33.572048,Ormosia_napoensis:33.572048,Ormosia_nanningensis:33.572048,Ormosia_pingbianensis:33.572048,Ormosia_eugeniifolia:33.572048,Ormosia_indurata:33.572048,Ormosia_pachycarpa:33.572048,Ormosia_microphylla_var.tomentosa:33.572048,Ormosia_pubescens:33.572048,Ormosia_semicastrata:33.572048,Ormosia_formosana:33.572048,Ormosia_nuda:33.572048,Ormosia_longipes:33.572048,Ormosia_microphylla:33.572048)ormosia:33.572048)ormosieae:33.572044,((((((Ammopiptanthus_mongolicus:14.388021)ammopiptanthus:14.388021):14.388021):14.388020,(((Salweenia_wardii:14.388021)salweenia:14.388021):14.388021):14.388020,(Euchresta_tubulosa_var.brevituba:28.776041,Euchresta_horsfieldii:28.776041,Euchresta_tubulosa:28.776041,Euchresta_japonica:28.776041)euchresta:28.776041):14.388020):14.388023):14.388016)genistoids:14.388023,((((((((Pterocarpus_indicus:12.789351)pterocarpus:12.789351):12.789351):12.789352)pterocarpus_group:12.789352,(((Dalbergia_cultrata:15.986690,Dalbergia_jingxiensis:15.986690,Dalbergia_mengsuoensis:15.986690)dalbergia:15.986690):15.986689,((Ormocarpum_cochinchinense:15.986690)ormocarpum:15.986690):15.986689)dalbergia_group:15.986691):12.789349)dalbergieae:12.789352):12.789352):12.789352):7.673615):7.673607):7.673615):7.673615)papilionoideae:7.673615):7.673599):7.673615):7.673615,((Bauhinia_brachycarpa:58.831020,Bauhinia_delavayi:58.831020,Bauhinia_hirsuta:58.831020,Bauhinia_hypoglauca:58.831020,Bauhinia_chalcophylla:58.831020,Bauhinia_coccinea_subsp.tonkinensis:58.831020,Bauhinia_glauca:58.831020,Bauhinia_blakeana:58.831020,Bauhinia_aurea:58.831020,Bauhinia_bohniana:58.831020,Bauhinia_viridescens:58.831020,Bauhinia_khasiana_var.tomentella:58.831020,Bauhinia_touranensis:58.831020,Bauhinia_comosa:58.831020,Bauhinia_erythropoda:58.831020,Bauhinia_purpurea:58.831020,Bauhinia_esquirolii:58.831020,Bauhinia_longistipes:58.831020)bauhinia:58.831020)cercideae:58.831017,(Saraca_griffithiana:88.246529,Saraca_dives:88.246529)Saraca:88.246529,(Fordia_microphylla:88.246529)Fordia:88.246529,(Sindora_glabra:88.246529)Sindora:88.246529,(Pterolobium_macropterum:88.246529,Pterolobium_punctatum:88.246529)Pterolobium:88.246529,(Sophora_prazeri:88.246529,Sophora_tonkinensis_var.purpurescens:88.246529)Sophora:88.246529,(Millettia_macrostachya:88.246529,Callerya_bonatiana:88.246529,Callerya_dormardi:88.246529,Callerya_nitida_var.minor:88.246529,Callerya_nitida_var.hirsutissima:88.246529,Callerya_fordii:88.246529,Millettia_pachyloba:88.246529,Millettia_erythrocalyx:88.246529,Millettia_cubitti:88.246529,Millettia_pachycarpa:88.246529,Millettia_pulchra_var.chinensis:88.246529,Callerya_cinerea:88.246529,Callerya_tsui:88.246529,Callerya_kiangsiensis:88.246529,Callerya_eurybotrya:88.246529,Millettia_lantsangensis:88.246529,Callerya_nitida:88.246529,Callerya_championi:88.246529,Callerya_speciosa:88.246529,Millettia_griffithi:88.246529,Callerya_congestiflora:88.246529,Millettia_ichthyochtona:88.246529,Callerya_sphaerosperma:88.246529,Millettia_velutina:88.246529,Millettia_pulchra_var.tomentosa:88.246529,Millettia_pulchra_var.laxior:88.246529,Millettia_leptobotrya:88.246529,Millettia_tetraptera:88.246529,Callerya_reticulata:88.246529,Millettia_sapindiifolia:88.246529,Callerya_reticulata_var.stenophylla:88.246529,Millettia_oraria:88.246529,Callerya_dielsiana:88.246529,Callerya_sericosema:88.246529,Callerya_dielsiana_var.solida:88.246529,Callerya_dielsiana_var.heterocarpa:88.246529,Millettia_pulchra:88.246529,Callerya_longipedunculata:88.246529,Callerya_oosperma:88.246529)Millettia:88.246529)fabaceae:7.673615,(((Polygala_latouchei:46.041668,Polygala_arillata:46.041668,Polygala_tricholopha:46.041668,Polygala_karensium:46.041668,Polygala_koi:46.041668,Polygala_oligosperma:46.041668,Polygala_didyma:46.041668,Polygala_caudata:46.041668,Polygala_karensium_var.obcordata:46.041668,Polygala_wattersii:46.041668,Polygala_globulifera_var.longiracemosa:46.041668,Polygala_resinosa:46.041668)Polygala:46.041668,(Xanthophyllum_oliganthum:46.041668,Xanthophyllum_yunnanense:46.041668)Xanthophyllum:46.041668,(Securidaca_inappendiculata:46.041668,Securidaca_yaoshanensis:46.041668)Securidaca:46.041668)polygalaceae:46.041664):46.041672):7.673615)fabales:7.673599):7.673615,((((((Glochidion_lanceolarium:34.531250,Glochidion_wrightii:34.531250,Glochidion_arborescens:34.531250,Glochidion_daltonii:34.531250,Glochidion_coccineum:34.531250,Glochidion_hirsutum:34.531250,Glochidion_oblatum:34.531250,Glochidion_eriocarpum:34.531250,Glochidion_lanceolatum:34.531250,Glochidion_lutescens:34.531250,Glochidion_suishaense:34.531250,Glochidion_ellipticum:34.531250,Glochidion_puberum:34.531250,Glochidion_kusukusense:34.531250,Glochidion_philippicum:34.531250,Glochidion_chademenosocarpum:34.531250,Glochidion_zeylanicum:34.531250,Glochidion_sphaerogynum:34.531250,Glochidion_khasicum:34.531250)Glochidion:34.531250,(Acalypha_hispida:34.531250)Acalypha:34.531250,(Actephila_excelsa:34.531250)Actephila:34.531250,(Putranjiva_formosana:34.531250)Putranjiva:34.531250,(Koilodepas_hainanense:34.531250)Koilodepas:34.531250,(Chaetocarpus_castanocarpus:34.531250)Chaetocarpus:34.531250,(Antidesma_nienkui:34.531250,Antidesma_maclurei:34.531250,Antidesma_ghaesembilla:34.531250,Antidesma_hainanense:34.531250,Antidesma_hontaushanense:34.531250,Antidesma_fordii:34.531250,Antidesma_montanum:34.531250,Antidesma_japonicum:34.531250,Antidesma_sootepense:34.531250,Antidesma_bunius:34.531250,Antidesma_acidum:34.531250,Antidesma_montanum_var.microphyllum:34.531250)Antidesma:34.531250,(Aporusa_villosa:34.531250,Aporusa_planchoniana:34.531250,Aporusa_dioica:34.531250,Aporusa_yunnanensis:34.531250)Aporusa:34.531250,(Lasiococca_comberi_var.pseudoverticillata:34.531250)Lasiococca:34.531250,(Suregada_glomerulata:34.531250,Suregada_aequorea:34.531250)Suregada:34.531250,(Microdesmis_caseariifolia:34.531250)Microdesmis:34.531250,(Drypetes_littoralis:34.531250,Drypetes_obtusa:34.531250,Drypetes_arcuatinervia:34.531250,Drypetes_hainanensis:34.531250,Drypetes_indica:34.531250,Drypetes_hoaensis:34.531250,Drypetes_congestiflora:34.531250,Drypetes_cumingii:34.531250,Drypetes_integrifolia:34.531250,Drypetes_perreticulata:34.531250,Drypetes_longistipitata:34.531250)Drypetes:34.531250,(Bischofia_javanica:34.531250)Bischofia:34.531250,(Blachia_andamanica:34.531250,Blachia_chunii:34.531250)Blachia:34.531250,(Sauropus_delavayi:34.531250,Sauropus_reticulatus:34.531250,Sauropus_macranthus:34.531250)Sauropus:34.531250,(Croton_tiglium:34.531250,Croton_damayeshu:34.531250,Croton_purpurascens:34.531250,Croton_chunianus:34.531250,Croton_laevigatus:34.531250,Croton_merrillianus:34.531250,Croton_howii:34.531250,Croton_lauioides:34.531250,Croton_caudatus:34.531250,Croton_mangelong:34.531250,Croton_lachnocarpus:34.531250,Croton_euryphyllus:34.531250,Croton_laniflorus:34.531250,Croton_hancei:34.531250,Croton_cnidophyllus:34.531250,Croton_yanhui:34.531250,Croton_cascarilloides:34.531250,Croton_kongensis:34.531250,Croton_yunnanensis:34.531250,Croton_joufra:34.531250)Croton:34.531250,(Excoecaria_agallocha:34.531250,Excoecaria_cochinchinensis:34.531250,Excoecaria_venenata:34.531250,Excoecaria_kawakamii:34.531250,Excoecaria_formosana:34.531250,Excoecaria_acerifolia:34.531250)Excoecaria:34.531250,(Macaranga_henryi:34.531250,Macaranga_lowii:34.531250,Macaranga_sampsonii:34.531250,Macaranga_andamanica:34.531250,Macaranga_pustulata:34.531250,Macaranga_sinensis:34.531250,Macaranga_kurzii:34.531250,Macaranga_indica:34.531250,Macaranga_denticulata:34.531250)Macaranga:34.531250,(Flueggea_leucopyra:34.531250,Flueggea_acicularis:34.531250)Flueggea:34.531250,(Trewia_nudiflora:34.531250)Trewia:34.531250,(Trigonostemon_fragilis:34.531250,Trigonostemon_xyphophylloides:34.531250,Trigonostemon_bonianus:34.531250,Trigonostemon_chinensis:34.531250,Trigonostemon_flavidus:34.531250,Trigonostemon_thyrsoideus:34.531250,Trigonostemon_howii:34.531250)Trigonostemon:34.531250,(Aleurites_moluccana:34.531250)Aleurites:34.531250,(Megistostigma_yunnanense:34.531250)Megistostigma:34.531250,(Ostodes_paniculata:34.531250,Ostodes_katharinae:34.531250)Ostodes:34.531250,(Melanolepis_multiglandulosa:34.531250)Melanolepis:34.531250,(Cephalomappa_sinensis:34.531250)Cephalomappa:34.531250,(Dimorphocalyx_poilanei:34.531250)Dimorphocalyx:34.531250,(Leptopus_fangdingianus:34.531250)Leptopus:34.531250,(Richeriella_gracilis:34.531250)Richeriella:34.531250,(Cladogynos_orientalis:34.531250)Cladogynos:34.531250,(Claoxylon_indicum:34.531250,Claoxylon_brachyandrum:34.531250)Claoxylon:34.531250,(Cleidiocarpon_cavaleriei:34.531250)Cleidiocarpon:34.531250,(Cleidion_brevipetiolatum:34.531250,Cleidion_bracteosum:34.531250,Cleidion_javanicum:34.531250)Cleidion:34.531250,(Cleistanthus_sumatranus:34.531250,Cleistanthus_macrophyllus:34.531250,Cleistanthus_concinnus:34.531250,Cleistanthus_petelotii:34.531250,Cleistanthus_tonkinensis:34.531250,Cleistanthus_pedicellatus:34.531250,Cleistanthus_tomentosus:34.531250)Cleistanthus:34.531250,(Baccaurea_ramiflora:34.531250)Baccaurea:34.531250,(Baliospermum_bilobatum:34.531250)Baliospermum:34.531250,(Cnesmone_mairei:34.531250,Cnesmone_hainanensis:34.531250)Cnesmone:34.531250,(Endospermum_chinense:34.531250)Endospermum:34.531250,(Epiprinus_siletianus:34.531250)Epiprinus:34.531250,(Erismanthus_sinensis:34.531250)Erismanthus:34.531250,(Euphorbia_royleana:34.531250,Euphorbia_hainanensis:34.531250)Euphorbia:34.531250,(Breynia_fruticosa:34.531250,Breynia_rostrata:34.531250)Breynia:34.531250,(Bridelia_insulana:34.531250,Bridelia_tomentosa:34.531250,Bridelia_stipularis:34.531250,Bridelia_affinis:34.531250,Bridelia_parvifolia:34.531250)Bridelia:34.531250,(Mallotus_philippensis:34.531250,Mallotus_peltatus:34.531250,Mallotus_barbatus_var.croizatianus:34.531250,Mallotus_repandus:34.531250,Mallotus_tetracoccus:34.531250,Mallotus_PHIlippensis_var.reticulatus:34.531250,Mallotus_microcarpus:34.531250,Mallotus_anomalus:34.531250,Mallotus_japonicus:34.531250,Mallotus_yunnanensis:34.531250,Mallotus_barbatus_var.pedicellaris:34.531250,Mallotus_esquirolii:34.531250)Mallotus:34.531250)euphorbiaceae:34.531250):34.531250,(((((Adenia_penangiana:23.020834,Adenia_cardiophylla:23.020834)Adenia:23.020834,(Passiflora_jugorum:23.020834,Passiflora_eberhardtii:23.020834,Passiflora_siamica:23.020834)Passiflora:23.020834)passifloraceae:23.020832):23.020836,(((Homalium_breviracemosum:23.020834,Homalium_paniculiflorum:23.020834,Homalium_kwangsiense:23.020834,Homalium_stenophyllum:23.020834,Homalium_kainantense:23.020834,Homalium_mollissimum:23.020834,Homalium_ceylanicum:23.020834,Homalium_cochinchinense:23.020834,Homalium_phanerophlebium:23.020834,Homalium_sabiifolium:23.020834)Homalium:23.020834,(Bennettiodendron_leprosipes:23.020834)Bennettiodendron:23.020834,(Itoa_orientalis_var.glabrescens:23.020834,Itoa_orientalis:23.020834)Itoa:23.020834,(Casearia_velutina:23.020834,Casearia_membranacea_f.membranacea:23.020834,Casearia_glomerata:23.020834,Casearia_tardieuae:23.020834)Casearia:23.020834,(Gynocardia_odorata:23.020834)Gynocardia:23.020834,(Xylosma_controversum_var.controversum:23.020834,Xylosma_congesta:23.020834,Xylosma_longifolium:23.020834)Xylosma:23.020834,(Hydnocarpus_annamensis:23.020834,Hydnocarpus_hainanensis:23.020834,Hydnocarpus_anthelminthica:23.020834)Hydnocarpus:23.020834,(Scolopia_chinensis:23.020834,Scolopia_saeva:23.020834,Scolopia_buxifolia:23.020834,Scolopia_oldhamii:23.020834)Scolopia:23.020834)salicaceae:23.020832):23.020836):23.020828):23.020836,((((Kandelia_candel:27.625000)Kandelia:27.625000,(Carallia_garciniaefolia:27.625000,Carallia_diplopetala:27.625000,Carallia_longipes:27.625000,Carallia_brachiata:27.625000)Carallia:27.625000,(Ceriops_tagal:27.625000)Ceriops:27.625000,(Pellacalyx_yunnanensis:27.625000)Pellacalyx:27.625000,(Rhizophora_stylosa:27.625000,Rhizophora_mucronata:27.625000,Rhizophora_apiculata:27.625000)Rhizophora:27.625000,(Bruguiera_sexangula:27.625000,Bruguiera_gymnorrhiza:27.625000,Bruguiera_cylindrica:27.625000)Bruguiera:27.625000)rhizophoraceae:27.625000,((Ixonanthes_chinensis:27.625000)Ixonanthes:27.625000,(Erythroxylum_sinensis:27.625000)Erythroxylum:27.625000)erythroxylaceae:27.625000):27.625000):27.625000,((Reinwardtia_indica:46.041668)Reinwardtia:46.041668,(Tirpitzia_ovoidea:46.041668,Tirpitzia_sinensis:46.041668)Tirpitzia:46.041668)linaceae:46.041664,((((Mesua_ferrea:27.625000)Mesua:27.625000,(Ochrocarpus_yunnanensis:27.625000)Ochrocarpus:27.625000,(Garcinia_xishuanbannaensis:27.625000,Garcinia_bracteata:27.625000,Garcinia_pedunculata:27.625000,Garcinia_xanthochymus:27.625000,Garcinia_oligantha:27.625000,Garcinia_subelliptica:27.625000,Garcinia_kwangsiensis:27.625000,Garcinia_erythrosepala:27.625000,Garcinia_subfalcata:27.625000,Garcinia_paucinervis:27.625000,Garcinia_linii:27.625000,Garcinia_oblongifolia:27.625000,Garcinia_mangostana:27.625000,Garcinia_multiflora:27.625000,Garcinia_nujiangensis:27.625000,Garcinia_esculenta:27.625000,Garcinia_tetralata:27.625000,Garcinia_yunnanensis:27.625000,Garcinia_cowa:27.625000,Garcinia_lancilimba:27.625000)Garcinia:27.625000,(Hypericum_pseudohenryi:27.625000,Hypericum_forrestii:27.625000,Hypericum_addingtonii:27.625000,Hypericum_hookerianum:27.625000,Hypericum_choisianum:27.625000,Hypericum_subalatum:27.625000,Hypericum_acmosepalum:27.625000,Hypericum_patulum:27.625000,Hypericum_monogynum:27.625000,Hypericum_subsessile:27.625000,Hypericum_cohaerens:27.625000,Hypericum_bellum:27.625000,Hypericum_reptans:27.625000,Hypericum_nakamurai:27.625000,Hypericum_uralum:27.625000,Hypericum_geminiflorum_subsp.geminiflorum:27.625000,Hypericum_formosanum:27.625000,Hypericum_curvisepalum:27.625000,Hypericum_augustinii:27.625000,Hypericum_henryi:27.625000,Hypericum_lagarocladum:27.625000,Hypericum_beanii:27.625000,Hypericum_lancasteri:27.625000)Hypericum:27.625000,(Calophyllum_membranaceum:27.625000,Calophyllum_polyanthum:27.625000,Calophyllum_inophyllum:27.625000,Calophyllum_blancoi:27.625000)Calophyllum:27.625000)clusiaceae:27.625000):27.625000):27.625000,(((Hiptage_candicans:34.531250,Hiptage_fraxinifolia:34.531250,Hiptage_multiflora:34.531250,Hiptage_benghalensis:34.531250,Hiptage_acuminata:34.531250,Hiptage_tianyangensis:34.531250,Hiptage_minor:34.531250,Hiptage_candicans_var.harmandiana:34.531250,Hiptage_benghalensis_var.tonkinensis:34.531250)Hiptage:34.531250,(Tristellateia_australasiae:34.531250)Tristellateia:34.531250,(Rhyssopterys_timoriensis:34.531250)Rhyssopterys:34.531250,(Aspidopterys_obcordata:34.531250,Aspidopterys_glabriuscula:34.531250,Aspidopterys_floribunda:34.531250,Aspidopterys_obcordata_var.hainanensis:34.531250,Aspidopterys_esquirolii:34.531250,Aspidopterys_nutans:34.531250,Aspidopterys_henryi:34.531250)Aspidopterys:34.531250)malpighiaceae:34.531250):34.531250,((Sinia_rhodoleuca:46.041668)Sinia:46.041668,(Gomphia_serrata:46.041668,Gomphia_striata:46.041668)Gomphia:46.041668)ochnaceae:46.041664,(((((Dichapetalum_gelonioides:23.020834,Dichapetalum_longipetalum:23.020834)Dichapetalum:23.020834)dichapetalaceae:23.020832):23.020836):23.020828):23.020836)malpighiales:23.020828,((((((Sloanea_chingiana:23.020832,Sloanea_leptocarpa:23.020832,Sloanea_mollis:23.020832,Sloanea_hemsleyana:23.020832,Sloanea_sterculiacea:23.020832,Sloanea_hainanensis:23.020832,Sloanea_sinensis:23.020832,Sloanea_dasycarpa:23.020832,Sloanea_integrifolia:23.020832,Sloanea_tomentosa:23.020832,Sloanea_xichouensis:23.020832,Sloanea_cordifolia:23.020832,Sloanea_changii:23.020832,Sloanea_sterculiacea_var.assamica:23.020832)Sloanea:23.020832,(Elaeocarpus_sikkimensis:23.020832,Elaeocarpus_balansae:23.020832,Elaeocarpus_braceanus:23.020832,Elaeocarpus_austro-yunnanensis:23.020832,Elaeocarpus_varunua:23.020832,Elaeocarpus_poilanei:23.020832,Elaeocarpus_duclouxii:23.020832,Elaeocarpus_decipiens:23.020832,Elaeocarpus_brachystachyus:23.020832,Elaeocarpus_hainanensis_var.brachyphyllus:23.020832,Elaeocarpus_lacunosus:23.020832,Elaeocarpus_multiflorus:23.020832,Elaeocarpus_duclouxii_var.funingensis:23.020832,Elaeocarpus_brachystachyus_var.fengii:23.020832,Elaeocarpus_atro-punctatus:23.020832,Elaeocarpus_chinensis:23.020832,Elaeocarpus_limitaneus:23.020832,Elaeocarpus_prunifolioides:23.020832,Elaeocarpus_auricomus:23.020832,Elaeocarpus_nitentifolius:23.020832,Elaeocarpus_sphaerocarpus:23.020832,Elaeocarpus_japonicus_var.lantsangensis:23.020832,Elaeocarpus_laoticus:23.020832,Elaeocarpus_glabripetalus_var.alatus:23.020832,Elaeocarpus_longlingensis:23.020832,Elaeocarpus_rugosus:23.020832,Elaeocarpus_lanceaefolius:23.020832,Elaeocarpus_subpetiolatus:23.020832,Elaeocarpus_sylvestris:23.020832,Elaeocarpus_bachmaensis:23.020832,Elaeocarpus_japonicus:23.020832,Elaeocarpus_hainanensis:23.020832,Elaeocarpus_glabripetalus:23.020832,Elaeocarpus_gymnogynus:23.020832,Elaeocarpus_dubius:23.020832,Elaeocarpus_argenteus:23.020832,Elaeocarpus_howii:23.020832,Elaeocarpus_angustifolius:23.020832,Elaeocarpus_decandrus:23.020832,Elaeocarpus_petiolatus:23.020832,Elaeocarpus_oblongilimbus:23.020832,Elaeocarpus_harmandii:23.020832)Elaeocarpus:23.020832)elaeocarpaceae:23.020836):23.020828):23.020836,(((Rourea_microphylla:28.776041,Rourea_caudata:28.776041)Rourea:28.776041,(Roureopsis_emarginata:28.776041)Roureopsis:28.776041)connaraceae:28.776043):28.776039):23.020836)oxalidales:23.020828):23.020844,(((Glyptopetalum_geloniifolium:46.041668,Glyptopetalum_ilicifolium:46.041668,Glyptopetalum_aquifolium:46.041668,Glyptopetalum_fengii:46.041668,Glyptopetalum_feddei:46.041668,Glyptopetalum_longepedunculatum:46.041668,Glyptopetalum_sclerocarpum:46.041668,Glyptopetalum_longipedicellatum:46.041668,Glyptopetalum_rhytidophyllum:46.041668)Glyptopetalum:46.041668,(Microtropis_thyrsiflora:46.041668,Microtropis_macrophyllus:46.041668,Microtropis_henryi:46.041668,Microtropis_tetragona:46.041668,Microtropis_oligantha:46.041668,Microtropis_fokienensis:46.041668,Microtropis_semipaniculata:46.041668,Microtropis_petelotii:46.041668,Microtropis_submembranacea:46.041668,Microtropis_hexandra:46.041668,Microtropis_osmanthoides:46.041668,Microtropis_japonica:46.041668,Microtropis_triflora:46.041668,Microtropis_paucinervia:46.041668,Microtropis_biflora:46.041668,Microtropis_pyramidalis:46.041668,Microtropis_reticulata:46.041668,Microtropis_obliquinervia:46.041668,Microtropis_discolor:46.041668,Microtropis_obscurinervia:46.041668,Microtropis_yunnanensis:46.041668)Microtropis:46.041668,(Salacia_aurantiaca:46.041668,Salacia_polysperma:46.041668,Salacia_glaucifolia:46.041668,Salacia_obovatilimba:46.041668,Salacia_cochinchinensis:46.041668,Salacia_sessiliflora:46.041668)Salacia:46.041668,(Loeseneriella_lenticellata:46.041668,Loeseneriella_yunnanensis:46.041668)Loeseneriella:46.041668,(Bhesa_robusta:46.041668)Bhesa:46.041668,(Pristimera_arborea:46.041668,Pristimera_cambodiana:46.041668,Pristimera_setulosa:46.041668)Pristimera:46.041668,(Maytenus_austroyunnanensis:46.041668,Maytenus_guangxiensis:46.041668,Maytenus_hainanensis:46.041668,Maytenus_hookeri:46.041668,Maytenus_confertiflorus:46.041668)Maytenus:46.041668,(Celastrus_paniculatus:46.041668,Celastrus_monospermus:46.041668,Celastrus_virens:46.041668,Celastrus_homaliifolius:46.041668,Celastrus_tonkinensis:46.041668)Celastrus:46.041668,(Dipentodon_sinicus:46.041668)Dipentodon:46.041668,(Gymnosporia_royleana:46.041668,Gymnosporia_diversifolia:46.041668,Gymnosporia_variabilis:46.041668,Gymnosporia_rufa:46.041668,Gymnosporia_tiaoloshanensis:46.041668,Gymnosporia_esquirolii:46.041668,Gymnosporia_jinyangensis:46.041668,Gymnosporia_emarginata:46.041668,Gymnosporia_graciliramula:46.041668,Gymnosporia_berberoides:46.041668,Gymnosporia_orbiculata:46.041668)Gymnosporia:46.041668,(Euonymus_potingensis:46.041668,Euonymus_tonkinensis:46.041668,Euonymus_parasimilis:46.041668,Euonymus_theacolus:46.041668,Euonymus_theifolius:46.041668,Euonymus_lucidus:46.041668,Euonymus_balansae:46.041668,Euonymus_myrianthus:46.041668,Euonymus_pallidifolius:46.041668,Euonymus_fortunei:46.041668,Euonymus_kengmaensis:46.041668,Euonymus_hainanensis:46.041668,Euonymus_hupehensis:46.041668,Euonymus_hukuangensis:46.041668,Euonymus_echinatus:46.041668,Euonymus_pseudovagans:46.041668,Euonymus_subcordatus:46.041668,Euonymus_chloranthoides:46.041668,Euonymus_chengii:46.041668,Euonymus_lichiangensis:46.041668,Euonymus_dielsianus:46.041668,Euonymus_tashiroi:46.041668,Euonymus_gibber:46.041668,Euonymus_salicifolius:46.041668,Euonymus_glaber:46.041668,Euonymus_bockii:46.041668,Euonymus_vaganoides:46.041668,Euonymus_venosus:46.041668,Euonymus_tingens:46.041668,Euonymus_ficoides:46.041668,Euonymus_carnosus:46.041668,Euonymus_acanthoxanthus:46.041668,Euonymus_bullatus:46.041668,Euonymus_aculeolus:46.041668,Euonymus_percoriaceus:46.041668,Euonymus_laxicymosus:46.041668,Euonymus_tsoi:46.041668,Euonymus_prismatomerioides:46.041668,Euonymus_aculeatus:46.041668,Euonymus_actinocarpus:46.041668,Euonymus_serratifolius:46.041668,Euonymus_vagans:46.041668,Euonymus_yunnanensis:46.041668,Euonymus_wilsonii:46.041668,Euonymus_dolichopus:46.041668,Euonymus_nitidus:46.041668)Euonymus:46.041668,(Pleurostylia_opposita:46.041668)Pleurostylia:46.041668)celastraceae:46.041664)celastrales:46.041672)celastrales_to_malpighiales:23.020828):7.673615)fabids:7.673615,((((((((Osbeckia_nepalensis_var.albiflora:31.232943,Osbeckia_chinensis_var.angustifolia:31.232943,Osbeckia_nepalensis:31.232943)Osbeckia:31.232943,(Memecylon_ligustrifolium_var.monocarpum:31.232943,Memecylon_polyanthum:31.232943,Memecylon_ligustrifolium:31.232943,Memecylon_hainanense:31.232943,Memecylon_nigrescens:31.232943,Memecylon_octocostatum:31.232943,Memecylon_luchuenense:31.232943,Memecylon_pauciflorum:31.232943,Memecylon_caeruleum:31.232943,Memecylon_scutellatum:31.232943,Memecylon_lanceolatum:31.232943)Memecylon:31.232943,(Styrophyton_caudatum:31.232943)Styrophyton:31.232943,(Barthea_barthei:31.232943)Barthea:31.232943,(Blastus_cochinchinensis:31.232943,Blastus_auriculatus:31.232943,Blastus_tsaii:31.232943)Blastus:31.232943,(Cyphotheca_montana:31.232943)Cyphotheca:31.232943,(Allomorphia_setosa:31.232943,Allomorphia_urophylla:31.232943)Allomorphia:31.232943,(Sporoxeia_sciadophila:31.232943,Sporoxeia_clavicalcarata:31.232943)Sporoxeia:31.232943,(Medinilla_nana:31.232943,Medinilla_septentrionalis:31.232943,Medinilla_assamica:31.232943,Medinilla_rubicunda:31.232943,Medinilla_hayataiana:31.232943,Medinilla_petelotii:31.232943,Medinilla_lanceata:31.232943,Medinilla_formosana:31.232943,Medinilla_fengii:31.232943,Medinilla_himalayana:31.232943)Medinilla:31.232943,(Melastoma_imbricatum:31.232943,Melastoma_dodecandrum:31.232943,Melastoma_sanguineum:31.232943,Melastoma_intermedium:31.232943,Melastoma_malabathricum:31.232943)Melastoma:31.232943,(Astronia_ferruginea:31.232943)Astronia:31.232943,(Bredia_oldhamii:31.232943,Bredia_hirsuta_var.scandens:31.232943)Bredia:31.232943)melastomataceae:31.232944,(((Crypteronia_paniculata:23.424707)Crypteronia:23.424707)crypteroniaceae:23.424709):23.424706):23.424713,(((Rhodomyrtus_tomentosa:29.280886)Rhodomyrtus:29.280886,(Syzygium_album:29.280886,Syzygium_claviflorum:29.280886,Syzygium_buxifolium:29.280886,Syzygium_lasianthifolium:29.280886,Syzygium_fruticosum:29.280886,Syzygium_jambos_var.tripinnatum:29.280886,Syzygium_myrsinifolium_var.grandiflorum:29.280886,Syzygium_densinervium_var.insulare:29.280886,Syzygium_oblancilimbum:29.280886,Syzygium_forrestii:29.280886,Syzygium_austro-yunnanense:29.280886,Syzygium_rockii:29.280886,Syzygium_baviense:29.280886,Syzygium_brachythyrsum:29.280886,Syzygium_globiflorum:29.280886,Syzygium_cinereum:29.280886,Syzygium_tephrodes:29.280886,Syzygium_oblatum:29.280886,Syzygium_gongshanense:29.280886,Syzygium_kwangtungense:29.280886,Syzygium_guangxiense:29.280886,Syzygium_handelii:29.280886,Syzygium_imitans:29.280886,Syzygium_hainanense:29.280886,Syzygium_infra-rubiginosum:29.280886,Syzygium_thumra:29.280886,Syzygium_melanophyllum:29.280886,Syzygium_bullockii:29.280886,Syzygium_kusukusense:29.280886,Syzygium_hancei:29.280886,Syzygium_rehderianum:29.280886,Syzygium_austrosinense:29.280886,Syzygium_cathayense:29.280886,Syzygium_buxifolioideum:29.280886,Syzygium_polypetaloideum:29.280886,Syzygium_toddalioides:29.280886,Syzygium_jienfunicum:29.280886,Syzygium_megacarpum:29.280886,Syzygium_simile:29.280886,Syzygium_grijsii:29.280886,Syzygium_malaccense:29.280886,Syzygium_vestitum:29.280886,Syzygium_chunianum:29.280886,Syzygium_salwinense:29.280886,Syzygium_jambos:29.280886,Syzygium_conspersipunctatum:29.280886,Syzygium_levinei:29.280886,Syzygium_laosense_var.quocense:29.280886,Syzygium_saxatile:29.280886,Syzygium_nervosum:29.280886,Syzygium_fluviatile:29.280886,Syzygium_szemaoense:29.280886,Syzygium_sichuanense:29.280886,Syzygium_tetragonum:29.280886,Syzygium_taiwanicum:29.280886,Syzygium_formosanum:29.280886,Syzygium_congestiflorum:29.280886,Syzygium_howii:29.280886,Syzygium_euonymifolium:29.280886,Syzygium_wenshanense:29.280886,Syzygium_cumini:29.280886,Syzygium_boisianum:29.280886,Syzygium_xizangense:29.280886,Syzygium_zeylanicum:29.280886,Syzygium_euphlebium:29.280886,Syzygium_tenuirhachis:29.280886,Syzygium_tsoongii:29.280886,Syzygium_stenocladum:29.280886,Syzygium_jambos_var.linearilimbum:29.280886,Syzygium_araiocladum:29.280886,Syzygium_balsameum:29.280886,Syzygium_odoratum:29.280886,Syzygium_acuminatissimum:29.280886,Syzygium_samarangense:29.280886,Syzygium_sterrophyllum:29.280886,Syzygium_paucivenium:29.280886,Syzygium_yunnanense:29.280886,Syzygium_cumini_var.tsoi:29.280886,Syzygium_lineatum:29.280886,Syzygium_rysopodum:29.280886,Syzygium_myrsinifolium:29.280886,Syzygium_championii:29.280886)Syzygium:29.280886,(Baeckea_frutescens:29.280886)Baeckea:29.280886,(Decaspermum_albociliatum:29.280886,Decaspermum_hainanensis:29.280886,Decaspermum_montanum:29.280886,Decaspermum_austro-hainanicum:29.280886,Decaspermum_glabrum:29.280886,Decaspermum_parviflorum:29.280886,Decaspermum_teretis:29.280886,Decaspermum_gracilentum:29.280886)Decaspermum:29.280886)myrtaceae:29.280888):29.280884):23.424706,(((Duabanga_grandiflora:35.137062)Duabanga:35.137062,(Sonneratia_alba:35.137062,Sonneratia_×_hainanensis:35.137062,Sonneratia_caseolaris:35.137062,Sonneratia_×_gulngai:35.137062,Sonneratia_ovata:35.137062,Sonneratia_apetala:35.137062)Sonneratia:35.137062)lythraceae:35.137062):35.137062):23.424713,((Anogeissus_acuminata_var.lanceolata:54.657654)Anogeissus:54.657654,(Lumnitzera_littorea:54.657654,Lumnitzera_racemosa:54.657654)Lumnitzera:54.657654,(Terminalia_nigrovenulosa:54.657654,Terminalia_myriocarpa:54.657654,Terminalia_myriocarpa_var.hirsuta:54.657654)Terminalia:54.657654,(Combretum_punctatum:54.657654,Combretum_alfredii:54.657654,Combretum_latifolium:54.657654,Combretum_roxburghii:54.657654,Combretum_wallichii:54.657654,Combretum_punctatum_subsp.squamosum:54.657654,Combretum_griffithii:54.657654,Combretum_pilosum:54.657654)Combretum:54.657654,(Calycopteris_floribunda:54.657654)Calycopteris:54.657654)combretaceae:54.657654)myrtales:23.424698):23.424713,((((((((((Glycosmis_craibii_var.glabra:39.041180,Glycosmis_montana:39.041180,Glycosmis_pseudoracemosa:39.041180,Glycosmis_lucida:39.041180,Glycosmis_craibii:39.041180,Glycosmis_cochinchinensis:39.041180,Glycosmis_pentaphylla:39.041180,Glycosmis_oligantha:39.041180,Glycosmis_xizangensis:39.041180,Glycosmis_parviflora:39.041180,Glycosmis_esquirolii:39.041180,Glycosmis_longifolia:39.041180)Glycosmis:39.041180,(Acronychia_pedunculata:39.041180)Acronychia:39.041180,(Citrus_×_polytrifolia:39.041180,Citrus_reticulata:39.041180,Citrus_hystrix:39.041180,Citrus_japonica:39.041180,Citrus_limon:39.041180,Citrus_aurantium:39.041180,Citrus_junos:39.041180,Citrus_medica:39.041180,Citrus_cavaleriei:39.041180,Citrus_maxima:39.041180)Citrus:39.041180,(Micromelum_falcatum:39.041180,Micromelum_integerrimum:39.041180)Micromelum:39.041180,(Zanthoxylum_simulans:39.041180,Zanthoxylum_pteracanthum:39.041180,Zanthoxylum_yuanjiangense:39.041180,Zanthoxylum_khasianum:39.041180,Zanthoxylum_tomentellum:39.041180,Zanthoxylum_dissitum_var.lanciforme:39.041180,Zanthoxylum_dissitum_var.acutiserratum:39.041180,Zanthoxylum_collinsae:39.041180,Zanthoxylum_piasezkii:39.041180,Zanthoxylum_acanthopodium:39.041180,Zanthoxylum_echinocarpum:39.041180,Zanthoxylum_dissitum_var.hispidum:39.041180,Zanthoxylum_dimorphophyllum_var.spinifolium:39.041180,Zanthoxylum_macranthum:39.041180,Zanthoxylum_multijugum:39.041180,Zanthoxylum_stipitatum:39.041180,Zanthoxylum_kwangsiense:39.041180,Zanthoxylum_esquirolii:39.041180,Zanthoxylum_scandens:39.041180,Zanthoxylum_oxyphylum:39.041180,Zanthoxylum_integrifolium:39.041180,Zanthoxylum_undulatifolium:39.041180,Zanthoxylum_leiboicum:39.041180,Zanthoxylum_liboense:39.041180,Zanthoxylum_nitidum:39.041180,Zanthoxylum_rhombifoliolatum:39.041180,Zanthoxylum_austrosinense:39.041180,Zanthoxylum_echinocarpum_var.tomentosum:39.041180,Zanthoxylum_myriacanthum_var.pubescens:39.041180,Zanthoxylum_austrosinense_var.pubescens:39.041180,Zanthoxylum_armatum_var.ferrugineum:39.041180,Zanthoxylum_glomeratum:39.041180,Zanthoxylum_laetum:39.041180,Zanthoxylum_wutaiense:39.041180,Zanthoxylum_schinifolium:39.041180,Zanthoxylum_calcicola:39.041180,Zanthoxylum_xichouense:39.041180,Zanthoxylum_stenophyllum:39.041180,Zanthoxylum_dissitum:39.041180)Zanthoxylum:39.041180,(Murraya_microphylla:39.041180,Murraya_alata:39.041180,Murraya_euchrestifolia:39.041180,Murraya_kwangsiensis:39.041180,Murraya_exotica:39.041180,Murraya_crenulata:39.041180,Murraya_tetramera:39.041180,Murraya_koenigii:39.041180)Murraya:39.041180,(Luvunga_scandens:39.041180)Luvunga:39.041180,(Toddalia_asiatica:39.041180)Toddalia:39.041180,(Skimmia_melanocarpa:39.041180,Skimmia_arborescens:39.041180,Skimmia_reevesiana:39.041180,Skimmia_laureola:39.041180)Skimmia:39.041180,(Melicope_viticina:39.041180,Melicope_chunii:39.041180,Melicope_glomerata:39.041180,Melicope_patulinervia:39.041180,Melicope_pteleifolia:39.041180,Melicope_triphylla:39.041180,Melicope_lunu-ankenda:39.041180,Melicope_semecarpifolia:39.041180)Melicope:39.041180,(Paramignya_confertifolia:39.041180)Paramignya:39.041180,(Clausena_lenis:39.041180,Clausena_hainanensis:39.041180,Clausena_lansium:39.041180,Clausena_excavata:39.041180,Clausena_vestita:39.041180,Clausena_yunnanensis_var.longgangensis:39.041180,Clausena_anisum-olens:39.041180,Clausena_odorata:39.041180,Clausena_emarginata:39.041180,Clausena_yunnanensis:39.041180)Clausena:39.041180,(Atalantia_henryi:39.041180,Atalantia_guillauminii:39.041180,Atalantia_fongkaica:39.041180,Atalantia_kwangtungensis:39.041180,Atalantia_dasycarpa:39.041180,Atalantia_acuminata:39.041180,Atalantia_buxifolia:39.041180)Atalantia:39.041180,(Maclurodendron_oligophlebia:39.041180)Maclurodendron:39.041180)rutaceae:39.041183,((Reinwardtiodendron_humile:52.705593)Reinwardtiodendron:52.705593,(Chisocheton_paniculatus:52.705593)Chisocheton:52.705593,((((((Dysoxylum_parasiticum:19.520590,Dysoxylum_grande:19.520590,Dysoxylum_mollissimum:19.520590,Dysoxylum_gotadhora:19.520590,Dysoxylum_excelsum:19.520590,Dysoxylum_cumingianum:19.520590,Dysoxylum_arborescens:19.520590,Dysoxylum_densiflorum:19.520590,Dysoxylum_hongkongense:19.520590,Dysoxylum_laxiracemosum:19.520590)dysoxylum:19.520590,(Aglaia_perviridis:19.520590,Aglaia_edulis:19.520590,Aglaia_odorata:19.520590,Aglaia_spectabilis:19.520590,Aglaia_elaeagnoidea:19.520590,Aglaia_rimosa:19.520590,Aglaia_lawii:19.520590,Aglaia_teysmanniana:19.520590)aglaia:19.520590):19.520592,((((Turraea_pubescens:11.712354)turraea:11.712354):11.712355):11.712353):11.712357):11.712353,(Walsura_robusta:35.137062,Walsura_pinnata:35.137062)walsura:35.137062):11.712357):11.712349)melioideae:11.712357,(Heynea_velutina:52.705593,Heynea_trijuga:52.705593)Heynea:52.705593,(((((Toona_fargesii:17.568531)toona:17.568531):17.568531):17.568531):17.568527)swietenioideae:17.568535,(Xylocarpus_granatum:52.705593)Xylocarpus:52.705593)meliaceae:11.712357,((Suriana_maritima:39.041180)Suriana:39.041180,(Ailanthus_fordii:39.041180,Ailanthus_triphysa:39.041180)Ailanthus:39.041180,(Harrisonia_perforata:39.041180)Harrisonia:39.041180,(Brucea_mollis:39.041180,Brucea_javanica:39.041180)Brucea:39.041180)simaroubaceae:39.041183):11.712349,((Acer_sino-oblongum:42.945297,Acer_yinkunii:42.945297,Acer_oblongum_var.omeiense:42.945297,Acer_oblongum:42.945297,Acer_laevigatum:42.945297,Acer_kwangnanense:42.945297,Acer_hilaense:42.945297,Acer_crassum:42.945297,Acer_poliophyllum:42.945297,Acer_pictum_subsp.pubigerum:42.945297,Acer_sycopseoides:42.945297,Acer_paxii:42.945297,Acer_lucidum:42.945297,Acer_fabri:42.945297,Acer_pinnatinervium:42.945297,Acer_laevigatum_var.salweenense:42.945297,Acer_shihweii:42.945297,Acer_laurinum:42.945297,Acer_wangchii:42.945297,Acer_coriaceifolium:42.945297,Acer_gracilifolium:42.945297,Acer_albo-purpurascens:42.945297)Acer:42.945297,(Amesiodendron_chinense:42.945297)Amesiodendron:42.945297,(Dodonaea_viscosa:42.945297)Dodonaea:42.945297,(Sapindus_tomentosus:42.945297)Sapindus:42.945297,(Boniodendron_minus:42.945297)Boniodendron:42.945297,(Nephelium_topengii:42.945297,Nephelium_chryseum:42.945297)Nephelium:42.945297,(Pometia_pinnata:42.945297)Pometia:42.945297,(Allophylus_chartaceus:42.945297,Allophylus_cobbe_var.velutinus:42.945297,Allophylus_timorensis:42.945297,Allophylus_dimorphus:42.945297,Allophylus_viridis:42.945297,Allophylus_longipes:42.945297)Allophylus:42.945297,(Paranephelium_hainanensis:42.945297)Paranephelium:42.945297,(Dimocarpus_yunnanensis:42.945297,Dimocarpus_fumatus_subsp.calcicola:42.945297,Dimocarpus_confinis:42.945297,Dimocarpus_longan:42.945297)Dimocarpus:42.945297,(Pavieasia_kwangsiensis:42.945297,Pavieasia_yunnanensis:42.945297)Pavieasia:42.945297,(Harpullia_cupanoides:42.945297)Harpullia:42.945297,(Xerospermum_bonii:42.945297)Xerospermum:42.945297,(Lepisanthes_rubiginosa:42.945297,Lepisanthes_senegalensis:42.945297,Lepisanthes_hainanensis:42.945297,Lepisanthes_unilocularis:42.945297)Lepisanthes:42.945297,(Arytera_littoralis:42.945297)Arytera:42.945297,(Mischocarpus_sundaicus:42.945297,Mischocarpus_hainanensis:42.945297,Mischocarpus_pentapetalus:42.945297)Mischocarpus:42.945297,(Delavaya_toxocarpa:42.945297)Delavaya:42.945297)sapindaceae:42.945297):11.712357,((((Dracontomelon_macrocarpum:28.109650,Dracontomelon_duperreanum:28.109650)Dracontomelon:28.109650,(Drimycarpus_anacardiifolius:28.109650)Drimycarpus:28.109650,(Mangifera_laurina:28.109650,Mangifera_persiciforma:28.109650,Mangifera_sylvatica:28.109650)Mangifera:28.109650,(Pegia_sarmentosa:28.109650)Pegia:28.109650,(Pistacia_weinmannifolia:28.109650)Pistacia:28.109650,(Buchanania_arborescens:28.109650)Buchanania:28.109650,(Semecarpus_longifolius:28.109650,Semecarpus_cuneiformis:28.109650,Semecarpus_reticulata:28.109650)Semecarpus:28.109650)anacardiaceae:28.109650,((Canarium_strictum:28.109650,Canarium_bengalense:28.109650,Canarium_subulatum:28.109650,Canarium_pimela:28.109650,Canarium_parvum:28.109650,Canarium_tonkinense:28.109650)Canarium:28.109650)burseraceae:28.109650):28.109650):28.109650):11.712357):11.712357)sapindales:11.712357,(((((((((Vatica_guangxiensis:18.582102,Vatica_mangachapoi:18.582102,Vatica_lanceifolia:18.582102)Vatica:18.582102,(Dipterocarpus_retusus:18.582102,Dipterocarpus_turbinatus:18.582102)Dipterocarpus:18.582102,(Parashorea_chinensis:18.582102)Parashorea:18.582102,(Hopea_hainanensis:18.582102,Hopea_reticulata:18.582102,Hopea_shingkeng:18.582102,Hopea_chinensis:18.582102)Hopea:18.582102,(Shorea_robusta:18.582102,Shorea_assamica:18.582102)Shorea:18.582102)dipterocarpaceae:18.582104):18.582100,((Helianthemum_songaricum:24.776136)Helianthemum:24.776136)cistaceae:24.776134):18.582100,((Pterospermum_yunnanense:30.970169,Pterospermum_lanceaefolium:30.970169,Pterospermum_proteus:30.970169,Pterospermum_heterophyllum:30.970169,Pterospermum_truncatolobatum:30.970169,Pterospermum_kingtungense:30.970169,Pterospermum_menglunense:30.970169,Pterospermum_niveum:30.970169)Pterospermum:30.970169,(Pterygota_alata:30.970169)Pterygota:30.970169,(Ambroma_augusta:30.970169)Ambroma:30.970169,(Kleinhovia_hospita:30.970169)Kleinhovia:30.970169,(Sterculia_impressinervis:30.970169,Sterculia_tonkinensis:30.970169,Sterculia_henryi_var.cuneata:30.970169,Sterculia_kingtungensis:30.970169,Sterculia_brevissima:30.970169,Sterculia_euosma:30.970169,Sterculia_hainanensis:30.970169,Sterculia_scandens:30.970169,Sterculia_principis:30.970169,Sterculia_pexa:30.970169,Sterculia_lanceolata:30.970169,Sterculia_subnobilis:30.970169,Sterculia_gengmaensis:30.970169,Sterculia_henryi:30.970169,Sterculia_hymenocalyx:30.970169,Sterculia_nobilis:30.970169,Sterculia_pinbienensis:30.970169,Sterculia_villosa:30.970169,Sterculia_ceramica:30.970169,Sterculia_lanceaefolia:30.970169,Sterculia_micrantha:30.970169,Sterculia_subracemosa:30.970169,Sterculia_cinnamomifolia:30.970169)Sterculia:30.970169,(Kydia_zizyphifolium:30.970169)Kydia:30.970169,(Reevesia_botingensis:30.970169,Reevesia_rotundifolia:30.970169,Reevesia_rubronervia:30.970169,Reevesia_lancifolia:30.970169,Reevesia_lofouensis:30.970169,Reevesia_pycnantha:30.970169,Reevesia_tomentosa:30.970169,Reevesia_shangszeensis:30.970169,Reevesia_pubescens:30.970169,Reevesia_pubescens_var.siamensis:30.970169,Reevesia_pubescens_var.xuefengensis:30.970169,Reevesia_glaucophylla:30.970169,Reevesia_orbicularifolia:30.970169,Reevesia_longipetiolata:30.970169)Reevesia:30.970169,(Diplodiscus_trichospermus:30.970169)Diplodiscus:30.970169,(Helicteres_obtusa:30.970169,Helicteres_isora:30.970169,Helicteres_lanceolata:30.970169,Helicteres_viscida:30.970169,Helicteres_angustifolia:30.970169,Helicteres_glabriuscula:30.970169,Helicteres_hirsuta:30.970169,Helicteres_elongata:30.970169)Helicteres:30.970169,(Microcos_chungii:30.970169,Microcos_paniculata:30.970169)Microcos:30.970169,(Heritiera_parvifolia:30.970169,Heritiera_littoralis:30.970169,Heritiera_angustata:30.970169)Heritiera:30.970169,(Hibiscus_tiliaceus:30.970169,Hibiscus_grewiifolius:30.970169,Hibiscus_rosa-sinensis:30.970169)Hibiscus:30.970169,(Berrya_cordifolia:30.970169)Berrya:30.970169,(Thespesia_lampas:30.970169,Thespesia_populnea:30.970169)Thespesia:30.970169,(Melhania_hamiltaniana:30.970169)Melhania:30.970169,(Grewia_biloba:30.970169,Grewia_permagna:30.970169,Grewia_retusifolia:30.970169,Grewia_henryi:30.970169,Grewia_falcata:30.970169,Grewia_rhombifolia:30.970169,Grewia_acuminata:30.970169,Grewia_celtidifolia:30.970169,Grewia_abutilifolia:30.970169,Grewia_concolor:30.970169,Grewia_angustisepala:30.970169,Grewia_biloba_var.parviflora:30.970169,Grewia_biloba_var.microphylla:30.970169,Grewia_chuniana:30.970169,Grewia_macropetala:30.970169)Grewia:30.970169,(Waltheria_indica:30.970169)Waltheria:30.970169,(Byttneria_aspera:30.970169,Byttneria_pilosa:30.970169,Byttneria_integrifolia:30.970169)Byttneria:30.970169)malvaceae:30.970169):18.582108,((Aquilaria_yunnanensis:37.164204)Aquilaria:37.164204,(Wikstroemia_trichotoma:37.164204,Wikstroemia_monnula:37.164204,Wikstroemia_fargesii:37.164204,Wikstroemia_pachyrachis:37.164204,Wikstroemia_mononectaria:37.164204,Wikstroemia_hainanensis:37.164204,Wikstroemia_huidongensis:37.164204,Wikstroemia_indica:37.164204,Wikstroemia_stenophylla:37.164204,Wikstroemia_micrantha:37.164204,Wikstroemia_angustifolia:37.164204,Wikstroemia_chuii:37.164204,Wikstroemia_longipaniculata:37.164204)Wikstroemia:37.164204,(Rhamnoneuron_balansae:37.164204)Rhamnoneuron:37.164204,(Eriosolena_composita:37.164204)Eriosolena:37.164204,(Daphne_retusa:37.164204,Daphne_papyracea:37.164204,Daphne_bholua:37.164204,Daphne_aurantiaca:37.164204,Daphne_macrantha:37.164204,Daphne_feddei_var.feddei:37.164204,Daphne_brevituba:37.164204,Daphne_emeiensis:37.164204,Daphne_rosmarinifolia:37.164204,Daphne_rhynchocarpa:37.164204,Daphne_acutiloba:37.164204,Daphne_jinyunensis_var.jinyunensis:37.164204,Daphne_tripartita:37.164204,Daphne_bholua_var.glacialis:37.164204,Daphne_kiusiana_var.atrocaulis:37.164204,Daphne_jinyunensis_var.ptilostyla:37.164204,Daphne_odora:37.164204,Daphne_papyracea_var.crassiuscula:37.164204,Daphne_depauperata:37.164204,Daphne_wangiana:37.164204,Daphne_holosericea:37.164204,Daphne_tangutica:37.164204,Daphne_holosericea_var.thibetensis:37.164204,Daphne_xichouensis:37.164204,Daphne_tenuiflora:37.164204,Daphne_gracilis:37.164204,Daphne_tangutica_var.wilsonii:37.164204,Daphne_axillaris:37.164204,Daphne_yunnanensis:37.164204,Daphne_longilobata:37.164204,Daphne_pedunculata:37.164204,Daphne_longituba:37.164204,Daphne_championii:37.164204,Daphne_purpurascens:37.164204)Daphne:37.164204)thymelaeaceae:37.164207):18.582092)malvales:18.582108,((((((((((Stixis_suaveolens:13.514256,Stixis_scandens:13.514256)Stixis:13.514256,(Borthwickia_trifoliata:13.514256)Borthwickia:13.514256,(Capparis_acutifolia:13.514256,Capparis_multiflora:13.514256,Capparis_dasyphylla:13.514256,Capparis_cantoniensis:13.514256,Capparis_hainanensis:13.514256,Capparis_sabiaefolia:13.514256,Capparis_viburnifolia:13.514256,Capparis_lanceolaris:13.514256,Capparis_membranifolia:13.514256,Capparis_masaikai:13.514256,Capparis_trichocarpa:13.514256,Capparis_pubiflora:13.514256,Capparis_pubifolia:13.514256,Capparis_fohaiensis:13.514256,Capparis_zeylanica:13.514256,Capparis_khuamak:13.514256,Capparis_sepiaria:13.514256,Capparis_versicolor:13.514256,Capparis_spinosa:13.514256,Capparis_floribunda:13.514256,Capparis_formosana:13.514256,Capparis_fengii:13.514256,Capparis_subsessilis:13.514256,Capparis_micracantha:13.514256,Capparis_urophylla:13.514256,Capparis_chingiana:13.514256,Capparis_bodinieri:13.514256,Capparis_wui:13.514256,Capparis_henryi:13.514256,Capparis_himalayensis:13.514256,Capparis_assamica:13.514256)Capparis:13.514256)capparaceae:13.514256):13.514256):13.514252):13.514259):13.514252):13.514259):13.514252):13.514252)brassicales:13.514267)malvales_to_brassicales:13.514252)huerteales_to_brassicales:13.514252):11.712341):11.712357,((((Turpinia_robusta:47.407146,Turpinia_pomifera:47.407146,Turpinia_macrosperma:47.407146,Turpinia_simplicifolia:47.407146,Turpinia_ovalifolia:47.407146,Turpinia_pomifera_var.minor:47.407146,Turpinia_montana:47.407146,Turpinia_indochinensis:47.407146,Turpinia_subsessilifolia:47.407146,Turpinia_affinis:47.407146)Turpinia:47.407146)staphyleaceae:47.407143,((((Stachyurus_retusus:28.444288,Stachyurus_obovatus:28.444288,Stachyurus_salicifolius:28.444288,Stachyurus_yunnanensis:28.444288)Stachyurus:28.444288)stachyuraceae:28.444286):28.444290):28.444283):28.444290)crossosomatales:28.444290):11.712357)malvids:11.712357):7.673599,(((Ampelocissus_hoabinhensis:57.552082,Ampelocissus_artemisiaefolia:57.552082,Ampelocissus_sikkimensis:57.552082)Ampelocissus:57.552082,(Leea_macrophylla:57.552082,Leea_philippinensis:57.552082,Leea_glabra:57.552082,Leea_indica:57.552082,Leea_compactiflora:57.552082,Leea_guineensis:57.552082,Leea_aequata:57.552082)Leea:57.552082,(Cissus_repanda_var.repanda:57.552082,Cissus_austro-yunnanensis:57.552082,Cissus_aristata:57.552082,Cissus_subtetragona:57.552082,Cissus_adnata:57.552082,Cissus_elongata:57.552082)Cissus:57.552082,(Tetrastigma_erubescens_var.monophyllum:57.552082,Tetrastigma_campylocarpum:57.552082,Tetrastigma_funingense:57.552082,Tetrastigma_subtetragonum:57.552082,Tetrastigma_erubescens_var.erubescens:57.552082,Tetrastigma_ceratopetalum:57.552082,Tetrastigma_cauliflorum:57.552082,Tetrastigma_jinghongense:57.552082,Tetrastigma_lanyuense:57.552082,Tetrastigma_lincangense:57.552082,Tetrastigma_venulosum:57.552082,Tetrastigma_serrulatum_var.pubinervium:57.552082,Tetrastigma_henryi:57.552082,Tetrastigma_delavayi:57.552082,Tetrastigma_retinervium_var.pubescens:57.552082,Tetrastigma_cruciatum:57.552082,Tetrastigma_formosanum:57.552082,Tetrastigma_obtectum_var.glabrum:57.552082,Tetrastigma_sichouense:57.552082,Tetrastigma_xishuangbannaense:57.552082,Tetrastigma_rumicispermum:57.552082,Tetrastigma_lenticellatum:57.552082,Tetrastigma_rumicispermum_var.lasiogynum:57.552082,Tetrastigma_yiwuense:57.552082,Tetrastigma_tsaianum:57.552082,Tetrastigma_apiculatum:57.552082,Tetrastigma_hypoglaucum:57.552082,Tetrastigma_sichouense_var.megalocarpum:57.552082)Tetrastigma:57.552082,(Yua_thomsonii_var.glaucescens:57.552082)Yua:57.552082)vitaceae:57.552086)vitales:57.552078)rosids:7.673615,(((((((Altingia_tenuifolia:35.241772,Altingia_multinervis:35.241772,Altingia_obovata:35.241772,Altingia_siamensis:35.241772,Altingia_gracilipes:35.241772,Altingia_excelsa:35.241772,Altingia_chinensis:35.241772,Altingia_yunnanensis:35.241772)Altingia:35.241772,(Distyliopsis_yunnanensis:35.241772,Distyliopsis_tutcheri:35.241772,Distyliopsis_dunnii:35.241772,Distyliopsis_salicifolia:35.241772,Distyliopsis_laurifolia:35.241772)Distyliopsis:35.241772,(Distylium_macrophyllum:35.241772,Distylium_cuspidatum:35.241772,Distylium_elaeagnoides:35.241772,Distylium_chungii:35.241772,Distylium_pingpienense:35.241772,Distylium_gracile:35.241772,Distylium_racemosum:35.241772,Distylium_buxifolium:35.241772,Distylium_myricoides:35.241772,Distylium_dunnianum:35.241772,Distylium_chinense:35.241772)Distylium:35.241772,(Chunia_bucklandioides:35.241772)Chunia:35.241772,(Loropetalum_lanceum:35.241772,Loropetalum_chinense_var.rubrum:35.241772,Loropetalum_chinense:35.241772,Loropetalum_subcordatum:35.241772)Loropetalum:35.241772,(Eustigma_balansae:35.241772,Eustigma_oblongifolium:35.241772,Eustigma_lenticellatum:35.241772)Eustigma:35.241772,(Exbucklandia_tonkinensis:35.241772,Exbucklandia_populnea:35.241772,Exbucklandia_longipetala:35.241772)Exbucklandia:35.241772,(Mytilaria_laosensis:35.241772)Mytilaria:35.241772,(Rhodoleia_macrocarpa:35.241772,Rhodoleia_championii:35.241772,Rhodoleia_forrestii:35.241772,Rhodoleia_parvipetala:35.241772,Rhodoleia_henryi:35.241772,Rhodoleia_stenopetala:35.241772)Rhodoleia:35.241772,(Sycopsis_triplinervia:35.241772,Sycopsis_sinensis:35.241772)Sycopsis:35.241772,(Corylopsis_alnifolia:35.241772)Corylopsis:35.241772,(Semiliquidambar_cathayensis:35.241772,Semiliquidambar_chingii:35.241772,Semiliquidambar_caudata:35.241772)Semiliquidambar:35.241772)hamamelidaceae:35.241768,(((Daphniphyllum_majus:26.431328,Daphniphyllum_oldhami:26.431328,Daphniphyllum_subverticillatum:26.431328,Daphniphyllum_macropodum:26.431328,Daphniphyllum_calycinum:26.431328,Daphniphyllum_himalense:26.431328,Daphniphyllum_angustifolium:26.431328,Daphniphyllum_paxianum:26.431328,Daphniphyllum_longeracemosum:26.431328,Daphniphyllum_chartaceum:26.431328)Daphniphyllum:26.431328)daphniphyllaceae:26.431328):26.431328):26.431320):26.431335):26.431320,(((((Pileostegia_viburnoides:30.836548,Pileostegia_tomentella:30.836548)Pileostegia:30.836548,(Itea_macrophylla:30.836548,Itea_yunnanensis:30.836548,Itea_ilicifolia:30.836548,Itea_riparia:30.836548,Itea_coriacea:30.836548,Itea_indochinensis_var.pubinervia:30.836548,Itea_indochinensis:30.836548,Itea_kiukiangensis:30.836548,Itea_chinensis:30.836548,Itea_oldhamii:30.836548,Itea_glutinosa:30.836548,Itea_parviflora:30.836548,Itea_amoena:30.836548,Itea_yangchunensis:30.836548,Itea_kwangsiensis:30.836548)Itea:30.836548,(Polyosma_cambodiana:30.836548)Polyosma:30.836548,(Deutzia_crassifolia:30.836548)Deutzia:30.836548,(Ribes_davidii:30.836548,Ribes_laurifolium:30.836548,Ribes_hunanense:30.836548,Ribes_henryi:30.836548,Ribes_davidii_var.ciliatum:30.836548,Ribes_davidii_var.lobatum:30.836548,Ribes_tianquanense:30.836548)Ribes:30.836548,(Decumaria_sinensis:30.836548)Decumaria:30.836548)saxifragaceae:30.836548):30.836548):30.836548):30.836548):26.431335)saxifragales:26.431320):7.673615,(((((((((((((((Uncaria_scandens:31.836597,Uncaria_laevigata:31.836597,Uncaria_yunnanensis:31.836597,Uncaria_sessilifructus:31.836597,Uncaria_homomalla:31.836597,Uncaria_macrophylla:31.836597,Uncaria_lancifolia:31.836597,Uncaria_rhynchophylla:31.836597,Uncaria_sinensis:31.836597)uncaria:31.836597):31.836601):31.836594,((((((Timonius_arboreus:18.192341)timonius:18.192341,guettarda_speciosa:36.384682):18.192341):18.192341):18.192337):18.192345):18.192345)cinchonoideae:18.192337,((((Mussaenda_simpliciloba:38.810326,Mussaenda_breviloba:38.810326,Mussaenda_mollissima:38.810326,Mussaenda_treutleri:38.810326,Mussaenda_hossei:38.810326,Mussaenda_decipiens:38.810326,Mussaenda_erosa:38.810326,Mussaenda_pingbianensis:38.810326,Mussaenda_divaricata_var.mollis:38.810326,Mussaenda_laxiflora:38.810326,Mussaenda_elliptica:38.810326,Mussaenda_sessilifolia:38.810326,Mussaenda_parviflora:38.810326)mussaenda:38.810326):38.810326,(((((((Gardenia_hainanensis:14.553872,Gardenia_angkorensis:14.553872,Gardenia_stenophylla:14.553872,Gardenia_augusta:14.553872)gardenia:14.553872):14.553873):14.553871,(((Tarenna_mollissima:14.553872,Tarenna_depauperata:14.553872,Tarenna_gracilipes:14.553872,Tarenna_polysperma:14.553872,Tarenna_lanceolata:14.553872,Tarenna_tsangii:14.553872,Tarenna_attenuata:14.553872,Tarenna_acutisepala:14.553872,Tarenna_lancilimba:14.553872,Tarenna_tsangii_f.elliptica:14.553872,Tarenna_zeylanica:14.553872,Tarenna_laui:14.553872,Tarenna_yunnanensis:14.553872,Tarenna_sinica:14.553872)tarenna:14.553872):14.553873):14.553871):14.553875,((Wendlandia_bouvardioides:24.256454,Wendlandia_brevipaniculata:24.256454,Wendlandia_tinctoria_subsp.barbata:24.256454,Wendlandia_scabra:24.256454,Wendlandia_pubigera:24.256454,Wendlandia_tinctoria_subsp.orientalis:24.256454,Wendlandia_formosana_subsp.breviflora:24.256454,Wendlandia_brevituba:24.256454,Wendlandia_tinctoria_subsp.floribunda:24.256454,Wendlandia_subalpina:24.256454,Wendlandia_guangdongensis:24.256454,Wendlandia_aberrans:24.256454,Wendlandia_cavaleriei:24.256454,Wendlandia_merrilliana:24.256454,Wendlandia_erythroxylon:24.256454,Wendlandia_tinctoria_subsp.intermedia:24.256454,Wendlandia_tinctoria_subsp.callitricha:24.256454,Wendlandia_jingdongensis:24.256454,Wendlandia_salicifolia:24.256454,Wendlandia_oligantha:24.256454,Wendlandia_luzoniensis:24.256454,Wendlandia_tinctoria_subsp.handelii:24.256454,Wendlandia_scabra_var.pilifera:24.256454,Wendlandia_tinctoria_subsp.affinis:24.256454,Wendlandia_villosa:24.256454,Wendlandia_speciosa:24.256454,Wendlandia_myriantha:24.256454,Wendlandia_litseifolia:24.256454,Wendlandia_pingpienensis:24.256454,Wendlandia_laxa:24.256454,Wendlandia_uvariifolia_subsp.pilosa:24.256454,Wendlandia_formosana:24.256454,Wendlandia_uvariifolia:24.256454,Wendlandia_longidens:24.256454,Wendlandia_augustinii:24.256454,Wendlandia_grandis:24.256454,Wendlandia_merrilliana_var.parvifolia:24.256454,Wendlandia_parviflora:24.256454,Wendlandia_ligustrina:24.256454,Wendlandia_longipedicellata:24.256454)wendlandia:24.256454):24.256454):14.553871,((((Ixora_henryi:17.464647,Ixora_paraopaca:17.464647,Ixora_finlaysoniana:17.464647,Ixora_amplexicaulis:17.464647,Ixora_auricularis:17.464647,Ixora_hainanensis:17.464647,Ixora_foonchowii:17.464647,Ixora_fulgens:17.464647,Ixora_chinensis:17.464647,Ixora_subsessilis:17.464647,Ixora_nienkui:17.464647,Ixora_effusa:17.464647,Ixora_tsangii:17.464647,Ixora_cephalophora:17.464647,Ixora_gracilis:17.464647,Ixora_philippinensis:17.464647,Ixora_yunnanensis:17.464647,Ixora_insignis:17.464647)ixora:17.464647):17.464645):17.464649):17.464645):14.553871):14.553871):14.553871)ixoroideae:14.553879):14.553864,(Urophyllum_tsaianum:80.046295,Urophyllum_chinense:80.046295,Urophyllum_parviflorum:80.046295)Urophyllum:80.046295,(Adina_pilulifera:80.046295)Adina:80.046295,(Lasianthus_rhinocerotis_subsp.xishuangbannaensis:80.046295,Lasianthus_biermanni_subsp.crassipedunculatus:80.046295,Lasianthus_chinensis:80.046295,Lasianthus_rigidus:80.046295,Lasianthus_austroyunnanensis:80.046295,Lasianthus_wardii:80.046295,Lasianthus_inodorus:80.046295,Lasianthus_biermanni:80.046295,Lasianthus_curtisii:80.046295,Lasianthus_hookeri:80.046295,Lasianthus_austrosinensis:80.046295,Lasianthus_chunii:80.046295,Lasianthus_calycinus:80.046295,Lasianthus_rhinocerotis_subsp.pedunculatus:80.046295,Lasianthus_hirsutus:80.046295,Lasianthus_hookeri_var.dunniana:80.046295,Lasianthus_chrysoneurus:80.046295,Lasianthus_japonicus_var.latifolius:80.046295,Lasianthus_obscurus:80.046295,Lasianthus_fordii:80.046295,Lasianthus_lancifolius:80.046295,Lasianthus_trichophlebus_var.latifolius:80.046295,Lasianthus_shimizui:80.046295,Lasianthus_japonicus:80.046295,Lasianthus_biflorus:80.046295,Lasianthus_formosensis:80.046295,Lasianthus_schmidtii:80.046295,Lasianthus_hispidulus:80.046295,Lasianthus_lucidus:80.046295,Lasianthus_henryi:80.046295,Lasianthus_sikkimensis:80.046295,Lasianthus_linearisepalus:80.046295,Lasianthus_micranthus:80.046295,Lasianthus_attenuatus:80.046295,Lasianthus_verticillatus:80.046295,Lasianthus_japonicus_subsp.longicaudus:80.046295,Lasianthus_chevalieri:80.046295,Lasianthus_filipes:80.046295,Lasianthus_trichophlebus:80.046295)Lasianthus:80.046295,(Metadina_trichotoma:80.046295)Metadina:80.046295,(Diplospora_dubia:80.046295,Diplospora_fruticosa:80.046295,Diplospora_mollissima:80.046295)Diplospora:80.046295,(Dunnia_sinensis:80.046295)Dunnia:80.046295,(Duperrea_pavettaefolia:80.046295)Duperrea:80.046295,(Pertusadina_hainanensis:80.046295)Pertusadina:80.046295,(Himalrandia_lichiangensis:80.046295)Himalrandia:80.046295,(Saprosma_hainanense:80.046295,Saprosma_crassipes:80.046295,Saprosma_merrillii:80.046295,Saprosma_ternatum:80.046295,Saprosma_henryi:80.046295)Saprosma:80.046295,(((((Mycetia_nepalensis:33.352623,Mycetia_macrocarpa:33.352623,Mycetia_brevisepala:33.352623,Mycetia_coriacea:33.352623,Mycetia_hainanensis:33.352623,Mycetia_hirta:33.352623,Mycetia_gracilis:33.352623,Mycetia_glandulosa:33.352623,Mycetia_yunnanica:33.352623,Mycetia_bracteata:33.352623,Mycetia_longiflora:33.352623,Mycetia_longifolia:33.352623)mycetia:33.352623,(Hedyotis_dianxiensis:33.352623,Hedyotis_scandens:33.352623)hedyotis:33.352623):33.352623,((((Psychotria_prainii:20.011574,Psychotria_tutcheri:20.011574,Psychotria_manillensis:20.011574,Psychotria_serpens:20.011574,Psychotria_calocarpa:20.011574,Psychotria_symplocifolia:20.011574)psychotria:20.011574):20.011574,(Morinda_undulata:30.017361,Morinda_leiantha:30.017361,Morinda_brevipes:30.017361,Morinda_citrifolia:30.017361,Morinda_hainanensis:30.017361,Morinda_angustifolia:30.017361,Morinda_officinalis_var.hirsuta:30.017361,Morinda_nanlingensis:30.017361,Morinda_nanlingensis_var.pauciflora:30.017361,Morinda_brevipes_var.stenophylla:30.017361,Morinda_villosa:30.017361,Morinda_cinnamomifoliata:30.017361,Morinda_callicarpaefolia:30.017361)morinda:30.017361):20.011574):20.011574):20.011574,(Ophiorrhiza_lignosa:60.034721)ophiorrhiza:60.034721):20.011581)rubioideae:20.011566,(Brachytome_hainanensis:80.046295,Brachytome_hirtellata_var.glabrescens:80.046295)Brachytome:80.046295,(Myrioneuron_nutans:80.046295)Myrioneuron:80.046295,(Fagerlindia_scandens:80.046295)Fagerlindia:80.046295,(Nauclea_officinalis:80.046295)Nauclea:80.046295,(Neonauclea_tsaiana:80.046295,Neonauclea_truncata:80.046295,Neonauclea_sessilifolia:80.046295,Neonauclea_griffithii:80.046295)Neonauclea:80.046295,(Trailliaedoxa_gracilis:80.046295)Trailliaedoxa:80.046295,(Porterandia_sericantha:80.046295)Porterandia:80.046295,(Sinoadina_racemosa:80.046295)Sinoadina:80.046295,(Prismatomeris_tetrantra:80.046295)Prismatomeris:80.046295,(Aidia_yunnanensis:80.046295,Aidia_pycnantha:80.046295,Aidia_cochinchinensis:80.046295,Aidia_shweliensis:80.046295,Aidia_canthioides:80.046295)Aidia:80.046295,(Canthium_dicoccum:80.046295,Canthium_simile:80.046295,Canthium_dicoccum_var.obovatifolium:80.046295)Canthium:80.046295,(Alleizettella_leucocarpa:80.046295)Alleizettella:80.046295,(Oxyceros_sinensis:80.046295,Oxyceros_griffithii:80.046295,Oxyceros_evenosa:80.046295)Oxyceros:80.046295,(Guihaiothamnus_acaulis:80.046295)Guihaiothamnus:80.046295,(Xanthophytum_kwangtungense:80.046295)Xanthophytum:80.046295,(Tarennoidea_wallichii:80.046295)Tarennoidea:80.046295,(Coelospermum_kanehirae:80.046295)Coelospermum:80.046295,(Coptosapelta_diffusa:80.046295)Coptosapelta:80.046295,(Hyptianthera_stricta:80.046295)Hyptianthera:80.046295,(Schizomussaenda_dehiscens:80.046295)Schizomussaenda:80.046295,(Damnacanthus_major:80.046295,Damnacanthus_giganteus:80.046295,Damnacanthus_hainanensis:80.046295,Damnacanthus_indicus:80.046295,Damnacanthus_labordei:80.046295,Damnacanthus_officinarum:80.046295,Damnacanthus_tsaii:80.046295,Damnacanthus_henryi:80.046295,Damnacanthus_macrophyllus:80.046295)Damnacanthus:80.046295,(Silvianthus_tonkinensis:80.046295)Silvianthus:80.046295)rubiaceae:14.553879,(((((Vallaris_indecora:68.939400)Vallaris:68.939400,(Alstonia_rupestris:68.939400,Alstonia_mairei:68.939400,Alstonia_neriifolia:68.939400,Alstonia_macrophylla:68.939400,Alstonia_henryi:68.939400,Alstonia_yunnanensis:68.939400,Alstonia_rostrata:68.939400)Alstonia:68.939400,(Alyxia_odorata:68.939400,Alyxia_levinei:68.939400,Alyxia_insularis:68.939400,Alyxia_sinensis:68.939400,Alyxia_villilimba:68.939400,Alyxia_menglungensis:68.939400,Alyxia_fascicularis:68.939400,Alyxia_schlechteri:68.939400,Alyxia_marginata:68.939400,Alyxia_reinwardii:68.939400,Alyxia_siamensis:68.939400)Alyxia:68.939400,(Amalocalyx_yunnanensis:68.939400)Amalocalyx:68.939400,(Stelmatocrypton_khasianum:68.939400)Stelmatocrypton:68.939400,(Stephanotis_pilosa:68.939400,Stephanotis_mucronata:68.939400,Stephanotis_saxatilis:68.939400)Stephanotis:68.939400,(Kopsia_lancibracteolata:68.939400)Kopsia:68.939400,(Anodendron_affine:68.939400,Anodendron_benthamianum:68.939400)Anodendron:68.939400,(Rauvolfia_serpentina:68.939400)Rauvolfia:68.939400,(Melodinus_khasianus:68.939400,Melodinus_yunnanensis:68.939400,Melodinus_angustifolius:68.939400,Melodinus_axillaris:68.939400)Melodinus:68.939400,(Streptocaulon_griffithii:68.939400)Streptocaulon:68.939400,(Strophanthus_caudatus:68.939400)Strophanthus:68.939400,(Chonemorpha_splendens:68.939400,Chonemorpha_eriostylis:68.939400,Chonemorpha_griffithii:68.939400,Chonemorpha_megacalyx:68.939400)Chonemorpha:68.939400,(Dregea_sinensis_var.corrugata:68.939400,Dregea_yunnanensis:68.939400,Dregea_volubilis:68.939400)Dregea:68.939400,(Heterostemma_oblongifolium:68.939400,Heterostemma_grandiflorum:68.939400,Heterostemma_brownii:68.939400,Heterostemma_siamicum:68.939400,Heterostemma_wallichii:68.939400)Heterostemma:68.939400,(Periploca_floribunda:68.939400,Periploca_forrestii:68.939400,Periploca_calophylla:68.939400)Periploca:68.939400,(Holarrhena_pubescens:68.939400)Holarrhena:68.939400,(Beaumontia_brevituba:68.939400,Beaumontia_pitardii:68.939400,Beaumontia_murtonii:68.939400,Beaumontia_khasiana:68.939400)Beaumontia:68.939400,(Bousigonia_angustifolia:68.939400)Bousigonia:68.939400,(Cryptolepis_sinensis:68.939400,Cryptolepis_buchananii:68.939400)Cryptolepis:68.939400,(Toxocarpus_wightianus:68.939400,Toxocarpus_wangianus:68.939400,Toxocarpus_villosus:68.939400,Toxocarpus_himalensis:68.939400,Toxocarpus_villosus_var.thorelii:68.939400,Toxocarpus_fuscus:68.939400,Toxocarpus_aurantiacus:68.939400)Toxocarpus:68.939400,(Trachelospermum_brevistylum:68.939400,Trachelospermum_bodinieri:68.939400,Trachelospermum_jasminoides:68.939400,Trachelospermum_dunnii:68.939400,Trachelospermum_asiaticum:68.939400,Trachelospermum_axillare:68.939400)Trachelospermum:68.939400,(Pottsia_grandiflora:68.939400,Pottsia_laxiflora:68.939400)Pottsia:68.939400,(Aganosma_schlechteriana:68.939400)Aganosma:68.939400,(Carissa_carandas:68.939400,Carissa_macrocarpa:68.939400,Carissa_spinarum:68.939400,Carissa_edulis:68.939400)Carissa:68.939400,(Marsdenia_koi:68.939400,Marsdenia_officinalis:68.939400,Marsdenia_medogensis:68.939400,Marsdenia_tenacissima:68.939400)Marsdenia:68.939400,(Gongronema_nepalense:68.939400)Gongronema:68.939400,(Goniostemma_punctatum:68.939400)Goniostemma:68.939400,(Cerbera_manghas:68.939400)Cerbera:68.939400,(Parepigynum_funingense:68.939400)Parepigynum:68.939400,(Gymnema_foetidum:68.939400,Gymnema_longiretinaculatum:68.939400,Gymnema_latifolium:68.939400,Gymnema_sylvestre:68.939400,Gymnema_yunnanense:68.939400)Gymnema:68.939400,(Wrightia_pubescens:68.939400)Wrightia:68.939400,(Tabernaemontana_bufalina:68.939400,Tabernaemontana_corymbosa:68.939400,Tabernaemontana_bovina:68.939400)Tabernaemontana:68.939400,(Epigynum_auritum:68.939400)Epigynum:68.939400,(Hunteria_zeylanica:68.939400)Hunteria:68.939400,(Ichnocarpus_frutescens:68.939400)Ichnocarpus:68.939400,(((((((((((((Secamone_sinica:12.255893,Secamone_likiangensis:12.255893)secamone:12.255893)secamonoideae:12.255892,(((dregea_sinensis:9.191919):9.191919):9.191919)asclepiadoideae:9.191919):9.191921):9.191917):9.191921):9.191917,(beaumontia_grandiflora:36.767677):36.767677):9.191917,strophanthus_divaricatus:82.727272):9.191925):9.191917):9.191917,(thevetia_peruviana:55.151516):55.151516):9.191925):9.191917,((tabernaemontana_divaricata:42.895626)tabernaemontaneae:42.895626):42.895622):9.191925,(Sindechites_henryi:68.939400)Sindechites:68.939400,(Calotropis_gigantea:68.939400)Calotropis:68.939400,(Tylophora_hui:68.939400,Tylophora_insulana:68.939400,Tylophora_koi:68.939400,Tylophora_ovata:68.939400)Tylophora:68.939400,(Genianthus_laurifolius:68.939400)Genianthus:68.939400,(alstonia_scholaris:68.939400)alstonieae:68.939400)apocynaceae:9.191910):9.191925,((Strychnos_cathayensis:52.087543,Strychnos_ignatii:52.087543,Strychnos_nitida:52.087543,Strychnos_angustiflora:52.087543,Strychnos_axillaris:52.087543)Strychnos:52.087543,(Fagraea_ceilanica:52.087543)Fagraea:52.087543,(Gelsemium_elegans:52.087543)Gelsemium:52.087543)loganiaceae:52.087547):9.191910):9.191925)gentianales:9.191925,((((((Osmanthus_heterophyllus:36.111115,Osmanthus_serrulatus:36.111115,Osmanthus_armatus:36.111115,Osmanthus_marginatus:36.111115,Osmanthus_pubipedicellatus:36.111115,Osmanthus_venosus:36.111115,Osmanthus_henryi:36.111115,Osmanthus_fragrans:36.111115,Osmanthus_cooperi:36.111115,Osmanthus_matsumuranus:36.111115,Osmanthus_lanceolatus:36.111115,Osmanthus_delavayi:36.111115,Osmanthus_fordii:36.111115,Osmanthus_didymopetalus:36.111115,Osmanthus_urceolatus:36.111115,Osmanthus_reticulatus:36.111115,Osmanthus_enervius:36.111115,Osmanthus_gracilinervis:36.111115,Osmanthus_attenuatus:36.111115,Osmanthus_hainanensis:36.111115,Osmanthus_suavis:36.111115,Osmanthus_minor:36.111115,Osmanthus_yunnanensis:36.111115,Osmanthus_marginatus_var.longissimus:36.111115)Osmanthus:36.111115,(Jasminum_humile_var.microphyllum:36.111115,Jasminum_subglandulosum:36.111115,Jasminum_microcalyx:36.111115,Jasminum_mesnyi:36.111115,Jasminum_nintooides:36.111115,Jasminum_yuanjiangense:36.111115,Jasminum_rufohirtum:36.111115,Jasminum_cinnamomifolium:36.111115,Jasminum_lang:36.111115,Jasminum_urophyllum:36.111115,Jasminum_duclouxii:36.111115,Jasminum_attenuatum:36.111115,Jasminum_×_stephanense:36.111115,Jasminum_subhumile:36.111115,Jasminum_polyanthum:36.111115,Jasminum_laurifolium:36.111115,Jasminum_beesianum:36.111115,Jasminum_sinense:36.111115,Jasminum_seguinii:36.111115,Jasminum_elongatum:36.111115,Jasminum_nervosum:36.111115,Jasminum_lanceolarium:36.111115,Jasminum_hongshuihoense:36.111115,Jasminum_dispermum:36.111115,Jasminum_officinale:36.111115,Jasminum_grandiflorum:36.111115,Jasminum_floridum:36.111115)Jasminum:36.111115,(Chionanthus_ramiflorus_var.grandiflorus:36.111115)Chionanthus:36.111115,(Myxopyrum_pierrei:36.111115,Myxopyrum_smilacifolium:36.111115)Myxopyrum:36.111115,(Ligustrum_pricei:36.111115,Ligustrum_robustum:36.111115,Ligustrum_obovatilimbum:36.111115,Ligustrum_obtusifolium_subsp.mirophyllu:36.111115,Ligustrum_lianum:36.111115,Ligustrum_expansum:36.111115,Ligustrum_henryi:36.111115,Ligustrum_sempervirens:36.111115,Ligustrum_compactum_var.velutinum:36.111115,Ligustrum_lucidum:36.111115,Ligustrum_confusum:36.111115,Ligustrum_obtusifolium:36.111115,Ligustrum_amamianum:36.111115,Ligustrum_tenuipes:36.111115,Ligustrum_gracile:36.111115,Ligustrum_xingrenense:36.111115,Ligustrum_strongylophyllum:36.111115,Ligustrum_morrisonense:36.111115,Ligustrum_yunguiense:36.111115,Ligustrum_longitubum:36.111115,Ligustrum_compactum:36.111115,Ligustrum_delavayanum:36.111115,Ligustrum_pedunculare:36.111115)Ligustrum:36.111115,(Syringa_meyeri_var.spontanea:36.111115,Syringa_wardii:36.111115)Syringa:36.111115,(Olea_brachiata:36.111115,Olea_tetragonoclada:36.111115,Olea_guangxiensis:36.111115,Olea_hainanensis:36.111115,Olea_rosea:36.111115,Olea_europaea:36.111115,Olea_laxiflora:36.111115,Olea_caudatilimba:36.111115,Olea_salicifolia:36.111115,Olea_neriifolia:36.111115,Olea_paniculata:36.111115,Olea_parvilimba:36.111115,Olea_ferruginea:36.111115,Olea_yuennanensis:36.111115)Olea:36.111115)oleaceae:36.111115):36.111115,((((((((((Adhatoda_vasica:13.131314)Adhatoda:13.131314,(Chroesthes_lanceolata:13.131314)Chroesthes:13.131314,(Phlogacanthus_curviflorus:13.131314,Phlogacanthus_pubinervius:13.131314)Phlogacanthus:13.131314,(Barleria_cristata:13.131314,Barleria_integrisepala:13.131314,Barleria_purpureosepala:13.131314)Barleria:13.131314,(Cystacanthus_paniculatus:13.131314,Cystacanthus_yunnanensis:13.131314,Cystacanthus_yangzekiangensis:13.131314,Cystacanthus_affinis:13.131314)Cystacanthus:13.131314,(Thunbergia_coccinea:13.131314)Thunbergia:13.131314,(Semnostachya_longispicata:13.131314)Semnostachya:13.131314,(Calophanoides_yunnanensis:13.131314,Calophanoides_wardii:13.131314,Calophanoides_xantholeuca:13.131314)Calophanoides:13.131314,(Mananthes_panduriformis:13.131314,Mananthes_microdonta:13.131314)Mananthes:13.131314)acanthaceae:13.131311,((Radermachera_sinica:13.131313,Radermachera_yunnanensis:13.131313,Radermachera_glandulosa:13.131313,Radermachera_hainanensis:13.131313,Radermachera_pentandra:13.131313,Radermachera_frondosa:13.131313,Radermachera_microcalyx:13.131313)Radermachera:13.131313,(Dolichandrone_spathacea:13.131313)Dolichandrone:13.131313,(Nyctocalos_pinnata:13.131313,Nyctocalos_brunfelsiiflorum:13.131313)Nyctocalos:13.131313,(Markhamia_stipulata:13.131313)Markhamia:13.131313,(Mayodendron_igneum:13.131313)Mayodendron:13.131313,(Pauldopia_ghorta:13.131313)Pauldopia:13.131313,(Millingtonia_hortensis:13.131313)Millingtonia:13.131313)bignoniaceae:13.131313):13.131317,(((Tsoongia_axillariflora:13.131314)Tsoongia:13.131314,(Premna_bracteata:13.131314,Premna_latifolia:13.131314,Premna_flavescens:13.131314,Premna_confinis:13.131314,Premna_crassa_var.yui:13.131314,Premna_rubroglandulosa:13.131314,Premna_fulva:13.131314,Premna_velutina:13.131314,Premna_acutata:13.131314,Premna_chevalieri:13.131314,Premna_interrupta:13.131314,Premna_subcapitata:13.131314,Premna_mekongensis:13.131314,Premna_urticifolia:13.131314,Premna_puberula_var.bodinieri:13.131314,Premna_punicea:13.131314,Premna_henryana:13.131314,Premna_laevigata:13.131314,Premna_oligantha:13.131314,Premna_crassa:13.131314,Premna_scoriarum:13.131314,Premna_scandens:13.131314,Premna_latifolia_var.cuneata:13.131314,Premna_yunnanensis:13.131314,Premna_racemosa:13.131314)Premna:13.131314,(Sphenodesme_mollis:13.131314,Sphenodesme_involucrata:13.131314)Sphenodesme:13.131314,(Vitex_yunnanensis:13.131314,Vitex_vestita:13.131314,Vitex_quinata:13.131314,Vitex_quinata_var.puberula:13.131314)Vitex:13.131314,(Symphorema_involucratum:13.131314)Symphorema:13.131314,(Avicennia_marina:13.131314)Avicennia:13.131314,(Congea_chinensis:13.131314,Congea_tomentosa:13.131314)Congea:13.131314)verbenaceae:13.131315):13.131313):13.131313,(((Chelonopsis_odontochila:16.414143,Chelonopsis_odontochila_var.smithii:16.414143,Chelonopsis_mollissima:16.414143,Chelonopsis_pseudobracteata:16.414143,Chelonopsis_bracteata:16.414143,Chelonopsis_lichiangensis:16.414143,Chelonopsis_rosea:16.414143,Chelonopsis_abbreviata:16.414143)Chelonopsis:16.414143,(Ocimum_sanctum:16.414143)Ocimum:16.414143,(Leucosceptrum_canum:16.414143)Leucosceptrum:16.414143,(Rostrinucula_dependens:16.414143)Rostrinucula:16.414143,(Elsholtzia_penduliflora:16.414143)Elsholtzia:16.414143,(Colebrookea_oppositifolia:16.414143)Colebrookea:16.414143,(Coleus_xanthanthus:16.414143)Coleus:16.414143,(Colquhounia_compta_var.mekongensis:16.414143,Colquhounia_coccinea_var.mollis:16.414143,Colquhounia_compta:16.414143,Colquhounia_sequinii:16.414143,Colquhounia_elegans:16.414143)Colquhounia:16.414143,(Eurysolen_gracilis:16.414143)Eurysolen:16.414143,(Geniosporum_coloratum:16.414143)Geniosporum:16.414143)lamiaceae:16.414143):16.414143):13.131310):13.131317,((Myoporum_bontioides:30.639732)Myoporum:30.639732,(Brandisia_kwangsiensis:30.639732,Brandisia_rosea:30.639732,Brandisia_hancei:30.639732,Brandisia_glabrescens:30.639732,Brandisia_glabrescens_var.hypochrysa:30.639732,Brandisia_discolor:30.639732,Brandisia_racemosa:30.639732)Brandisia:30.639732)scrophulariaceae:30.639732):13.131317):13.131310,(((Anna_submontana:29.545456,Anna_mollifolia:29.545456)Anna:29.545456,(Rhabdothamnopsis_sinensis:29.545456)Rhabdothamnopsis:29.545456,(Hemiboeopsis_longisepala:29.545456)Hemiboeopsis:29.545456,(Beccarinda_erythrotricha:29.545456)Beccarinda:29.545456,(Loxostigma_brevipetiolatum:29.545456,Loxostigma_mekongense:29.545456)Loxostigma:29.545456,(Boeica_porosa:29.545456,Boeica_yunnanensis:29.545456)Boeica:29.545456,(Lysionotus_serratus:29.545456,Lysionotus_serratus_var.pterocaulis:29.545456,Lysionotus_forrestii:29.545456,Lysionotus_pauciflorus:29.545456,Lysionotus_sessilifolius:29.545456,Lysionotus_denticulosus:29.545456,Lysionotus_kwangsiensis:29.545456,Lysionotus_aeschynanthoides:29.545456,Lysionotus_gamosepalus:29.545456,Lysionotus_sulphureus:29.545456,Lysionotus_pauciflorus_var.indutus:29.545456,Lysionotus_heterophyllus_var.lasianthus:29.545456,Lysionotus_heterophyllus_var.mollis:29.545456,Lysionotus_pubescens:29.545456,Lysionotus_metuoensis:29.545456,Lysionotus_chingii:29.545456,Lysionotus_sangzhiensis:29.545456,Lysionotus_atropurpureus:29.545456,Lysionotus_petelotii:29.545456,Lysionotus_levipes:29.545456,Lysionotus_gracilis:29.545456,Lysionotus_microphyllus:29.545456,Lysionotus_heterophyllus_var.heterophyllus:29.545456,Lysionotus_involucratus:29.545456,Lysionotus_longipedunculatus:29.545456,Lysionotus_oblongifolius:29.545456)Lysionotus:29.545456,(Cyrtandra_umbellifera:29.545456)Cyrtandra:29.545456,(Aeschynanthus_humilis:29.545456,Aeschynanthus_planipetiolatus:29.545456,Aeschynanthus_pachytrichus:29.545456,Aeschynanthus_mimetes:29.545456,Aeschynanthus_austroyunnanensis:29.545456,Aeschynanthus_superbus:29.545456,Aeschynanthus_buxifolius:29.545456,Aeschynanthus_andersonii:29.545456,Aeschynanthus_acuminatus:29.545456,Aeschynanthus_lasianthus:29.545456,Aeschynanthus_mengxingensis:29.545456,Aeschynanthus_lancilimbus:29.545456,Aeschynanthus_macranthus:29.545456,Aeschynanthus_hookeri:29.545456,Aeschynanthus_tengchungensis:29.545456,Aeschynanthus_linearifolius:29.545456,Aeschynanthus_tubulosus:29.545456,Aeschynanthus_stenosepalus:29.545456,Aeschynanthus_wardii:29.545456,Aeschynanthus_angustioblongus:29.545456,Aeschynanthus_bracteatus:29.545456,Aeschynanthus_lineatus:29.545456,Aeschynanthus_denticuliger:29.545456,Aeschynanthus_poilanei:29.545456,Aeschynanthus_sinolongicalyx:29.545456,Aeschynanthus_acuminatissimus:29.545456,Aeschynanthus_longicaulis:29.545456)Aeschynanthus:29.545456,(Paraboea_swinhoii:29.545456)Paraboea:29.545456,(Rhynchotechum_formosanum:29.545456,Rhynchotechum_vestitum:29.545456,Rhynchotechum_discolor:29.545456)Rhynchotechum:29.545456,(Leptoboea_multiflora:29.545456)Leptoboea:29.545456)gesneriaceae:29.545456):29.545456):13.131317):13.131317):13.131302)lamiales:13.131317,((((Blinkworthia_convolvuloides:34.141415)Blinkworthia:34.141415,(Neuropeltis_racemosa:34.141415)Neuropeltis:34.141415,(Porana_discifera:34.141415,Porana_sinensis:34.141415,Porana_sinensis_var.delavayi:34.141415,Porana_paniculata:34.141415)Porana:34.141415,(Argyreia_acuta:34.141415,Argyreia_baoshanensis:34.141415,Argyreia_cheliensis:34.141415,Argyreia_wallichii:34.141415,Argyreia_monosperma:34.141415,Argyreia_pierreana:34.141415,Argyreia_velutina:34.141415,Argyreia_fulvo-cymosa_var.fulvo-cymosa:34.141415,Argyreia_osyrensis_var.cinerea:34.141415,Argyreia_henryi_var.hypochrysa:34.141415,Argyreia_splendens:34.141415,Argyreia_marlipoensis:34.141415,Argyreia_eriocephala:34.141415,Argyreia_maymyo:34.141415,Argyreia_formosana:34.141415,Argyreia_capitata:34.141415,Argyreia_strigillosa:34.141415,Argyreia_lineariloba:34.141415,Argyreia_mastersii:34.141415,Argyreia_mollis:34.141415,Argyreia_henryi:34.141415)Argyreia:34.141415,(Erycibe_schmidtii:34.141415,Erycibe_henryi:34.141415,Erycibe_ferruginea:34.141415,Erycibe_subspicata:34.141415)Erycibe:34.141415)convolvulaceae:34.141418):34.141411)solanales:34.141418):13.131317,((Ehretia_changjiangensis:61.279465,Ehretia_laevis:61.279465,Ehretia_pingbianensis:61.279465,Ehretia_tsangii:61.279465,Ehretia_resinosa:61.279465,Ehretia_corylifolia:61.279465,Ehretia_dunniana:61.279465,Ehretia_confinis:61.279465,Ehretia_longiflora:61.279465)Ehretia:61.279465,(Tournefortia_sarmentosa:61.279465)Tournefortia:61.279465,(Carmona_microphylla:61.279465)Carmona:61.279465)boraginaceae:61.279465):9.191910,((Apodytes_dimidiata:64.343437)Apodytes:64.343437,(Natsiatopsis_thunbergiaefolia:64.343437)Natsiatopsis:64.343437,(Nothapodytes_foetida:64.343437,Nothapodytes_obtusifolia:64.343437,Nothapodytes_pittosporoides:64.343437)Nothapodytes:64.343437,(Iodes_balansae:64.343437,Iodes_seguini:64.343437,Iodes_cirrhosa:64.343437,Iodes_vitiginea:64.343437)Iodes:64.343437,(Mappianthus_iodoides:64.343437)Mappianthus:64.343437,(Gomphandra_tetrandra:64.343437)Gomphandra:64.343437,(Gonocaryum_lobbianum:64.343437,Gonocaryum_calleryanum:64.343437)Gonocaryum:64.343437,(Pittosporopsis_kerrii:64.343437)Pittosporopsis:64.343437,(Platea_latifolia:64.343437)Platea:64.343437)icacinaceae:64.343430)lamiids:9.191925,(((((((((((Acanthopanax_evodiaefolius_var.evodiaefolius:18.777777)Acanthopanax:18.777777,(Diplopanax_stachyanthus:18.777777)Diplopanax:18.777777,(Merrilliopanax_chinensis:18.777777,Merrilliopanax_listeri:18.777777)Merrilliopanax:18.777777,(Heteropanax_brevipedicellatus:18.777777,Heteropanax_chinensis:18.777777,Heteropanax_fragrans:18.777777,Heteropanax_nitentifolius:18.777777,Heteropanax_yunnanensis:18.777777)Heteropanax:18.777777,(Schefflera_rhododendrifolia:18.777777,Schefflera_hypoleuca:18.777777,Schefflera_leucantha:18.777777,Schefflera_khasiana:18.777777,Schefflera_macrophylla:18.777777,Schefflera_bodinieri:18.777777,Schefflera_brevipedicellata:18.777777,Schefflera_multinervia:18.777777,Schefflera_metcalfiana:18.777777,Schefflera_heptaphylla:18.777777,Schefflera_arboricola:18.777777,Schefflera_insignis:18.777777,Schefflera_elata:18.777777,Schefflera_hainanensis:18.777777,Schefflera_hoi:18.777777,Schefflera_petelotii:18.777777,Schefflera_hypoleucoides:18.777777,Schefflera_lociana:18.777777,Schefflera_marlipoensis:18.777777,Schefflera_elliptica:18.777777,Schefflera_napuoensis:18.777777,Schefflera_pauciflora:18.777777,Schefflera_shweliensis:18.777777,Schefflera_delavayi:18.777777,Schefflera_taiwaniana:18.777777,Schefflera_fengii:18.777777,Schefflera_wardii:18.777777,Schefflera_parvifoliolata:18.777777,Schefflera_minutistellata:18.777777,Schefflera_chapana:18.777777,Schefflera_pes-avis:18.777777,Schefflera_chinensis:18.777777)Schefflera:18.777777,(Brassaiopsis_hispida:18.777777,Brassaiopsis_simplicifolia:18.777777,Brassaiopsis_producta:18.777777,Brassaiopsis_quercifolia:18.777777,Brassaiopsis_glomerulata:18.777777,Brassaiopsis_moumingensis:18.777777,Brassaiopsis_ficifolia:18.777777,Brassaiopsis_shweliensis:18.777777,Brassaiopsis_triloba:18.777777,Brassaiopsis_tibetana:18.777777,Brassaiopsis_angustifolia:18.777777,Brassaiopsis_ciliata:18.777777,Brassaiopsis_tripteris:18.777777,Brassaiopsis_stellata:18.777777,Brassaiopsis_ferruginea:18.777777,Brassaiopsis_chengkangensis:18.777777)Brassaiopsis:18.777777,(Fatsia_japonica:18.777777,Fatsia_polycarpa:18.777777)Fatsia:18.777777,(Nothopanax_delavayi:18.777777,Nothopanax_davidii:18.777777)Nothopanax:18.777777,(Gamblea_ciliata:18.777777)Gamblea:18.777777,(Trevesia_palmata:18.777777)Trevesia:18.777777,(Tupidanthus_calyptratus:18.777777)Tupidanthus:18.777777,(Sinopanax_formosanus:18.777777)Sinopanax:18.777777,(Dendropanax_oligodontus:18.777777,Dendropanax_proteus:18.777777,Dendropanax_chevalieri:18.777777,Dendropanax_kwangsiensis:18.777777,Dendropanax_hainanensis:18.777777,Dendropanax_confertus:18.777777,Dendropanax_burmanicus:18.777777,Dendropanax_caloneurus:18.777777,Dendropanax_dentiger:18.777777,Dendropanax_bilocularis:18.777777,Dendropanax_productus:18.777777)Dendropanax:18.777777,(Pentapanax_longipes:18.777777,Pentapanax_hypoglaucus:18.777777,Pentapanax_parasiticus:18.777777,Pentapanax_tomentellus_var.distinctus:18.777777,Pentapanax_verticillatus:18.777777,Pentapanax_tomentellus:18.777777,Pentapanax_parasiticus_var.khasianus:18.777777,Pentapanax_castanopsisicola:18.777777,Pentapanax_wilsonii:18.777777,Pentapanax_subcordatus:18.777777,Pentapanax_henryi:18.777777,Pentapanax_fragrans:18.777777,Pentapanax_yunnanensis:18.777777,Pentapanax_racemosus:18.777777)Pentapanax:18.777777,(Hedera_nepalensis_var.sinensis:18.777777)Hedera:18.777777,(Tetrapanax_papyrifer:18.777777)Tetrapanax:18.777777,(Macropanax_undulatus:18.777777,Macropanax_oreophilus:18.777777,Macropanax_rosthornii:18.777777,Macropanax_decandrus:18.777777,Macropanax_paucinervis:18.777777,Macropanax_chienii:18.777777)Macropanax:18.777777,(Oplopanax_elatus:18.777777)Oplopanax:18.777777)araliaceae:18.777779):18.777775,((Pittosporum_leptosepalum:25.037035,Pittosporum_tenuivalvatum:25.037035,Pittosporum_planilobum:25.037035,Pittosporum_podocarpum:25.037035,Pittosporum_undulatifolium:25.037035,Pittosporum_daphniphylloides_var.adaphniphylloides:25.037035,Pittosporum_heterophyllum_var.ledoides:25.037035,Pittosporum_napaulense:25.037035,Pittosporum_johnstonianum:25.037035,Pittosporum_merrillianum:25.037035,Pittosporum_brevicalyx:25.037035,Pittosporum_omeiense:25.037035,Pittosporum_perryanum:25.037035,Pittosporum_tubiflorum:25.037035,Pittosporum_glabratum:25.037035,Pittosporum_kwangsiense:25.037035,Pittosporum_kweichowense:25.037035,Pittosporum_illicioides:25.037035,Pittosporum_tobira:25.037035,Pittosporum_podocarpum_var.hejiangensis:25.037035,Pittosporum_fulvipilosum:25.037035,Pittosporum_rehderianum_var.ternstroemioides:25.037035,Pittosporum_rehderianum:25.037035,Pittosporum_kweichowense_var.buxifolium:25.037035,Pittosporum_viburnifolium:25.037035,Pittosporum_subulisepalum:25.037035,Pittosporum_balansae:25.037035,Pittosporum_kunmingense:25.037035,Pittosporum_trigonocarpum:25.037035,Pittosporum_lenticellatum:25.037035,Pittosporum_kweichowense_var.podocarpifolium:25.037035,Pittosporum_podocarpum_var.molle:25.037035,Pittosporum_johnstonianum_var.glomerulatum:25.037035,Pittosporum_xylocarpum:25.037035,Pittosporum_daphniphylloides:25.037035,Pittosporum_oligophlebium:25.037035,Pittosporum_perglabratum:25.037035,Pittosporum_pauciflorum:25.037035,Pittosporum_saxicola:25.037035,Pittosporum_tonkinense:25.037035,Pittosporum_pentandrum_var.hainanense:25.037035,Pittosporum_tobira_var.calvescens:25.037035,Pittosporum_elevaticostatum:25.037035,Pittosporum_perryanum_var.linearifolium:25.037035,Pittosporum_glabratum_var.neriifolium:25.037035,Pittosporum_podocarpum_var.angustatum:25.037035,Pittosporum_henryi:25.037035,Pittosporum_parvicapsulare:25.037035,Pittosporum_parvilimbum:25.037035,Pittosporum_pulchrum:25.037035,Pittosporum_truncatum:25.037035,Pittosporum_kerrii:25.037035,Pittosporum_heterophyllum:25.037035,Pittosporum_paniculiferum:25.037035,Pittosporum_angustilimbum:25.037035,Pittosporum_balansae_var.angustifolium:25.037035,Pittosporum_pauciflorum_var.oblongum:25.037035,Pittosporum_reflexisepalum:25.037035,Pittosporum_crispulum:25.037035)Pittosporum:25.037035)pittosporaceae:25.037037):18.777779):18.777779):18.777779)apiales:18.777771,((((Viburnum_henryi:30.044443,Viburnum_leiocarpum_var.punctatum:30.044443,Viburnum_sempervirens:30.044443,Viburnum_davidii:30.044443,Viburnum_punctatum_var.lepidotulum:30.044443,Viburnum_lutescens:30.044443,Viburnum_hanceanum:30.044443,Viburnum_brachybotryum:30.044443,Viburnum_leiocarpum:30.044443,Viburnum_hainanense:30.044443,Viburnum_inopinatum:30.044443,Viburnum_chinshanense:30.044443,Viburnum_chunii:30.044443,Viburnum_atrocyaneum:30.044443,Viburnum_punctatum:30.044443,Viburnum_luzonicum:30.044443,Viburnum_formosanum_var.pubigerum:30.044443,Viburnum_congestum:30.044443,Viburnum_fordiae:30.044443,Viburnum_lancifolium:30.044443,Viburnum_corymbiflorum_subsp.malifolium:30.044443,Viburnum_propinquum:30.044443,Viburnum_corymbiflorum:30.044443,Viburnum_odoratissimum:30.044443,Viburnum_oliganthum:30.044443,Viburnum_cylindricum:30.044443,Viburnum_propinquum_var.mairei:30.044443,Viburnum_utile:30.044443,Viburnum_chingii:30.044443,Viburnum_squamulosum:30.044443,Viburnum_dalzielii:30.044443,Viburnum_cinnamomifolium:30.044443,Viburnum_longipedunculatum:30.044443,Viburnum_rhytidophyllum:30.044443,Viburnum_pyramidatum:30.044443)Viburnum:30.044443,(Lonicera_tricalysioides:30.044443,Lonicera_subaequalis:30.044443,Lonicera_hildebrandiana:30.044443,Lonicera_macrantha:30.044443,Lonicera_pampaninii:30.044443,Lonicera_calvescens:30.044443,Lonicera_japonica_var.chinensis:30.044443,Lonicera_confusa:30.044443,Lonicera_ligustrina_subsp.yunnanensis:30.044443,Lonicera_ligustrina:30.044443,Lonicera_crassifolia:30.044443,Lonicera_japonica:30.044443,Lonicera_pileata:30.044443,Lonicera_pileata_var.linearis:30.044443,Lonicera_macrantha_var.heterotricha:30.044443,Lonicera_fragrantissima_subsp.fragrantissima:30.044443,Lonicera_longiflora:30.044443,Lonicera_rhytidophylla:30.044443)Lonicera:30.044443,(Linnaea_borealis:30.044443)Linnaea:30.044443)caprifoliaceae:30.044445)dipsacales:30.044441):30.044441):18.777786):18.777771,((((((Scaevola_sericea:52.962963,Scaevola_hainanensis:52.962963)Scaevola:52.962963)goodeniaceae:52.962959,(((((((((((((((((((((Pluchea_indica:7.222222)pluchea:7.222222):7.222221):7.222223):7.222221):7.222221):7.222225):7.222221):7.222221):7.222221):7.222221):7.222221):7.222221):7.222229):7.222221):7.222221):7.222221):7.222221):7.222229):7.222214)asteraceae:7.222229):7.222214):7.222229):7.222214):7.222229)asterales:7.222214):7.222229,(((((Ilex_arisanensis:32.50000,Ilex_lohfauensis:32.50000,Ilex_chamaebuxus:32.500000,Ilex_championii:32.500000,Ilex_subcoriacea:32.500000,Ilex_tenuis:32.500000,Ilex_liangii:32.500000,Ilex_chengkouensis:32.500000,Ilex_crenata:32.500000,Ilex_bioritsensis:32.500000,Ilex_corallina_var.loeseneri:32.500000,Ilex_robustinervosa:32.500000,Ilex_strigillosa:32.500000,Ilex_robusta:32.500000,Ilex_intricata:32.500000,Ilex_dabieshanensis:32.500000,Ilex_formosana_var.macropyrena:32.500000,Ilex_latifolia:32.500000,Ilex_macrostigma:32.500000,Ilex_maximowicziana:32.500000,Ilex_dehongensis:32.500000,Ilex_dianguiensis:32.500000,Ilex_wardii:32.500000,Ilex_forrestii:32.500000,Ilex_punctatilimba:32.500000,Ilex_chinensis:32.500000,Ilex_yuiana:32.500000,Ilex_buergeri:32.500000,Ilex_brachyphylla:32.500000,Ilex_polypyrena:32.500000,Ilex_omeiensis:32.500000,Ilex_fengqingensis:32.500000,Ilex_fukienensis:32.500000,Ilex_excelsa:32.500000,Ilex_yunnanensis_var.gentilis:32.500000,Ilex_rockii:32.500000,Ilex_delavayi_var.exalta:32.500000,Ilex_hookeri:32.500000,Ilex_cornuta:32.500000,Ilex_memecylifolia:32.500000,Ilex_hylonoma_var.glabra:32.500000,Ilex_nuculicava_var.glabra:32.500000,Ilex_kwangtungensis:32.500000,Ilex_guangnanensis:32.500000,Ilex_pubescens_var.kwangsiensis:32.500000,Ilex_confertiflora_var.kwangsiensis:32.500000,Ilex_guizhouensis:32.500000,Ilex_szechwanensis_var.huiana:32.500000,Ilex_goshiensis:32.500000,Ilex_hainanensis:32.500000,Ilex_metabaptista:32.500000,Ilex_atrata:32.500000,Ilex_melanotricha:32.500000,Ilex_melanophylla:32.500000,Ilex_manneiensis:32.500000,Ilex_elmerrilliana:32.500000,Ilex_sterrophylla:32.500000,Ilex_centrochinensis:32.500000,Ilex_dasyphylla:32.500000,Ilex_buxoides:32.500000,Ilex_cinerea:32.500000,Ilex_tetramera:32.500000,Ilex_pseudomachilifolia:32.500000,Ilex_wattii:32.500000,Ilex_wangiana:32.500000,Ilex_lancilimba:32.500000,Ilex_tsiangiana:32.500000,Ilex_jiaolingensis:32.500000,Ilex_jinyunensis:32.500000,Ilex_cauliflora:32.500000,Ilex_gingtungensis:32.500000,Ilex_jiuwanshanensis:32.500000,Ilex_perlata:32.500000,Ilex_pedunculosa:32.500000,Ilex_tamii:32.500000,Ilex_franchetiana:32.500000,Ilex_kaushue:32.500000,Ilex_kunmingensis:32.500000,Ilex_latifrons:32.500000,Ilex_rarasanensis:32.500000,Ilex_angulata:32.500000,Ilex_pyrifolia:32.500000,Ilex_delavayi_var.comblriana:32.500000,Ilex_austro-sinensis:32.500000,Ilex_nitidissima:32.500000,Ilex_euryoides:32.500000,Ilex_suzukii:32.500000,Ilex_salicina:32.500000,Ilex_dunniana:32.500000,Ilex_cheniana:32.500000,Ilex_longzhouensis:32.500000,Ilex_ludianensis:32.500000,Ilex_tutcheri:32.500000,Ilex_viridis:32.500000,Ilex_marlipoensis:32.500000,Ilex_pernyi:32.500000,Ilex_excelsa_var.hypotricha:32.500000,Ilex_pubescens:32.500000,Ilex_trichocarpa:32.500000,Ilex_liana:32.500000,Ilex_pubilimba:32.500000,Ilex_szechwanensis_var.mollissima:32.500000,Ilex_dasyclada:32.500000,Ilex_miguensis:32.500000,Ilex_confertiflora:32.500000,Ilex_chingiana:32.500000,Ilex_medogensis:32.500000,Ilex_litseaefolia:32.500000,Ilex_delavayi_var.muliensis:32.500000,Ilex_nanchuanensis:32.500000,Ilex_nanningensis:32.500000,Ilex_machilifolia:32.500000,Ilex_subcrenata:32.500000,Ilex_subficoidea:32.500000,Ilex_sublongecaudata:32.500000,Ilex_ningdeensis:32.500000,Ilex_pingheensis:32.500000,Ilex_estriata:32.500000,Ilex_pingnanensis:32.500000,Ilex_syzygiophylla:32.500000,Ilex_stewardii:32.500000,Ilex_qianlingshanensis:32.500000,Ilex_hanceana:32.500000,Ilex_qingyuanensis:32.500000,Ilex_nuculicava_var.auctumnalis:32.500000,Ilex_integra:32.500000,Ilex_lonicerifolia:32.500000,Ilex_ficoidea:32.500000,Ilex_limii:32.500000,Ilex_triflora:32.500000,Ilex_godajam:32.500000,Ilex_umbellulata:32.500000,Ilex_corallina:32.500000,Ilex_peiradena:32.500000,Ilex_shennongjiaensis:32.500000,Ilex_verisimilis:32.500000,Ilex_shimeica:32.500000,Ilex_oligodonta:32.500000,Ilex_bidens:32.500000,Ilex_dicarpa:32.500000,Ilex_dipyrena:32.500000,Ilex_szechwanensis:32.500000,Ilex_suichangensis:32.500000,Ilex_formosana:32.500000,Ilex_sugerokii:32.500000,Ilex_rotunda:32.500000,Ilex_chuniana:32.500000,Ilex_cupreonitens:32.500000,Ilex_kobuskiana:32.500000,Ilex_glomerata:32.500000,Ilex_nuculicava:32.500000,Ilex_cyrtura:32.500000,Ilex_reticulata:32.500000,Ilex_retusifolia:32.500000,Ilex_venulosa:32.500000,Ilex_subodorata:32.500000,Ilex_wilsonii:32.500000,Ilex_wenchowensis:32.500000,Ilex_uraiensis:32.500000,Ilex_forrestii_var.glabra:32.500000,Ilex_tetramera_var.glabra:32.500000,Ilex_pentagona:32.500000,Ilex_wilsonii_var.handel-mazzettii:32.500000,Ilex_xizangensis:32.500000,Ilex_sikkimensis:32.500000,Ilex_lihuaensis:32.500000,Ilex_denticulata:32.500000,Ilex_hylonoma:32.500000,Ilex_venosa:32.500000,Ilex_tsangii:32.500000,Ilex_fargesii:32.500000,Ilex_ciliospinosa:32.500000,Ilex_graciliflora:32.500000,Ilex_gracilis:32.500000,Ilex_editicostata:32.500000,Ilex_fargesii_var.angustifolia:32.500000,Ilex_delavayi_var.linearifolia:32.500000,Ilex_delavayi:32.500000,Ilex_suaveolens:32.500000,Ilex_micropyrena:32.500000,Ilex_xiaojinensis:32.500000,Ilex_franchetiana_var.parvifolia:32.500000,Ilex_nothofagifolia:32.500000,Ilex_huiana:32.500000,Ilex_ferruginea:32.500000,Ilex_tugitakayamensis:32.500000,Ilex_yangchunensis:32.500000,Ilex_subrugosa:32.500000,Ilex_occulta:32.500000,Ilex_hirsuta:32.500000,Ilex_ficifolia:32.500000,Ilex_pubigera:32.500000,Ilex_cochinchinensis:32.500000,Ilex_yunnanensis:32.500000,Ilex_nubicola:32.500000,Ilex_hayataiana:32.500000,Ilex_dolichopoda:32.500000,Ilex_atrata_var.wangii:32.500000,Ilex_longecaudata:32.500000,Ilex_georgei:32.500000,Ilex_oblonga:32.500000,Ilex_maclurei:32.500000,Ilex_zhejiangensis:32.500000,Ilex_wuiana:32.500000,Ilex_chartaceifolia:32.500000,Ilex_sinica:32.500000,Ilex_intermedia:32.500000,Ilex_kengii:32.500000,Ilex_perryana:32.500000,Ilex_metabaptista_var.myrsinoides:32.500000)Ilex:32.500000,(Plagiopteron_suaveolens:32.500000)Plagiopteron:32.500000)aquifoliaceae:32.500000):32.500000):32.500000)aquifoliales:32.500000)campanulids:7.222229):7.222214,(((((((((Vaccinium_chamaebuxus:20.944445,Vaccinium_kengii:20.944445,Vaccinium_emarginatum:20.944445,Vaccinium_impressinerve:20.944445,Vaccinium_leucobotrys:20.944445,Vaccinium_albidens:20.944445,Vaccinium_moupinense:20.944445,Vaccinium_nummularia:20.944445,Vaccinium_conchophyllum:20.944445,Vaccinium_delavayi:20.944445,Vaccinium_pratense:20.944445,Vaccinium_arbutoides:20.944445,Vaccinium_fimbribracteatum:20.944445,Vaccinium_foetidissimum:20.944445,Vaccinium_trichocladum:20.944445,Vaccinium_fragile_var.mekongense:20.944445,Vaccinium_petelotii:20.944445,Vaccinium_dunalianum_var.megaphyllum:20.944445,Vaccinium_bulleyanum:20.944445,Vaccinium_brevipedicellatum:20.944445,Vaccinium_brachyandrum:20.944445,Vaccinium_carlesii:20.944445,Vaccinium_brachybotrys:20.944445,Vaccinium_pubicalyx_var.leucocalyx:20.944445,Vaccinium_omeiense:20.944445,Vaccinium_pseudospadiceum:20.944445,Vaccinium_glauco-album:20.944445,Vaccinium_papillatum:20.944445,Vaccinium_gaultheriifolium_var.glauco-rubrum:20.944445,Vaccinium_duclouxii_var.hirticaule:20.944445,Vaccinium_guangdongense:20.944445,Vaccinium_sinicum:20.944445,Vaccinium_hainanense:20.944445,Vaccinium_haitangense:20.944445,Vaccinium_urceolatum:20.944445,Vaccinium_oxycoccos:20.944445,Vaccinium_iteophyllum:20.944445,Vaccinium_glaucophyllum:20.944445,Vaccinium_sikkimense:20.944445,Vaccinium_mandarinorum:20.944445,Vaccinium_kachinense:20.944445,Vaccinium_chunii:20.944445,Vaccinium_subfalcatum:20.944445,Vaccinium_lamprophyllum:20.944445,Vaccinium_fimbricalyx:20.944445,Vaccinium_papulosum:20.944445,Vaccinium_randaiense:20.944445,Vaccinium_podocarpoideum:20.944445,Vaccinium_chengiae_var.pilosum:20.944445,Vaccinium_pubicalyx:20.944445,Vaccinium_bracteatum:20.944445,Vaccinium_pseudobullatum:20.944445,Vaccinium_bullatum:20.944445,Vaccinium_duclouxii_var.pubipes:20.944445,Vaccinium_gaultheriifolium:20.944445,Vaccinium_pubicalyx_var.anomalum:20.944445,Vaccinium_dendrocharis:20.944445,Vaccinium_chengiae:20.944445,Vaccinium_delavayi_subsp.merrillianum:20.944445,Vaccinium_cuspidifolium:20.944445,Vaccinium_supracostatum:20.944445,Vaccinium_chaetothrix:20.944445,Vaccinium_pseudorobustum:20.944445,Vaccinium_dunalianum_var.urophyllum:20.944445,Vaccinium_fragile:20.944445,Vaccinium_pseudotonkinense:20.944445,Vaccinium_iteophyllum_var.glandulosum:20.944445,Vaccinium_microcarpum:20.944445,Vaccinium_bracteatum_var.chinense:20.944445,Vaccinium_exaristatum:20.944445,Vaccinium_lanigerum:20.944445,Vaccinium_cavinerve:20.944445,Vaccinium_vitis-idaea:20.944445,Vaccinium_duclouxii:20.944445,Vaccinium_dunalianum:20.944445,Vaccinium_craspedotum:20.944445,Vaccinium_harmandianum:20.944445,Vaccinium_dunnianum:20.944445,Vaccinium_longicaudatum:20.944445,Vaccinium_kingdon-wardii:20.944445,Vaccinium_ardisioides:20.944445)Vaccinium:20.944445,(Chamaedaphne_calyculata:20.944445)Chamaedaphne:20.944445,(Ledum_palustre_var.dilatatum:20.944445,Ledum_palustre_var.decumbens:20.944445)Ledum:20.944445,(Phyllodoce_caerulea:20.944445)Phyllodoce:20.944445,(Pieris_japonica:20.944445,Pieris_formosa:20.944445,Pieris_swinhoei:20.944445)Pieris:20.944445,(Lyonia_macrocalyx:20.944445,Lyonia_ovalifolia_var.rubrovenia:20.944445,Lyonia_ovalifolia_var.hebecarpa:20.944445,Lyonia_ovalifolia_var.tomentosa:20.944445,Lyonia_ovalifolia_var.lanceolata:20.944445,Lyonia_ovalifolia_var.elliptica:20.944445,Lyonia_doyonensis:20.944445,Lyonia_ovalifolia:20.944445)Lyonia:20.944445,(Craibiodendron_scleranthum_var.kwangtungense:20.944445,Craibiodendron_stellatum:20.944445,Craibiodendron_henryi:20.944445,Craibiodendron_yunnanense:20.944445)Craibiodendron:20.944445,(Agapetes_aborensis:20.944445,Agapetes_leucocarpa:20.944445,Agapetes_mannii:20.944445,Agapetes_pseudo-griffithii:20.944445,Agapetes_marginata:20.944445,Agapetes_inopinata:20.944445,Agapetes_praeclara:20.944445,Agapetes_camelliifolia:20.944445,Agapetes_nutans:20.944445,Agapetes_megacarpa:20.944445,Agapetes_macrophylla:20.944445,Agapetes_pensilis:20.944445,Agapetes_obovata:20.944445,Agapetes_lacei:20.944445,Agapetes_listeri:20.944445,Agapetes_leiocarpa:20.944445,Agapetes_rubrobracteata:20.944445,Agapetes_brandisiana:20.944445,Agapetes_flava:20.944445,Agapetes_buxifolia:20.944445,Agapetes_neriifolia:20.944445,Agapetes_epacridea:20.944445,Agapetes_angulata:20.944445,Agapetes_mitrarioides:20.944445,Agapetes_salicifolia:20.944445,Agapetes_pyrolifolia:20.944445,Agapetes_malipoensis:20.944445,Agapetes_pubiflora:20.944445,Agapetes_burmanica:20.944445,Agapetes_medogensis:20.944445,Agapetes_lacei_var.tomentella:20.944445,Agapetes_forrestii:20.944445,Agapetes_lobbii:20.944445,Agapetes_miranda:20.944445,Agapetes_praestigiosa:20.944445,Agapetes_hyalocheilos:20.944445,Agapetes_griffithii:20.944445,Agapetes_lacei_var.glaberrima:20.944445,Agapetes_serpens:20.944445,Agapetes_ciliata:20.944445,Agapetes_brachypoda_var.gracilis:20.944445,Agapetes_linearifolia:20.944445,Agapetes_anonyma:20.944445,Agapetes_discolor:20.944445,Agapetes_oblonga:20.944445,Agapetes_refracta:20.944445,Agapetes_interdicta:20.944445,Agapetes_pilifera:20.944445,Agapetes_incurvata:20.944445,Agapetes_miniata:20.944445)Agapetes:20.944445,(Cassiope_nana:20.944445,Cassiope_pectinata:20.944445,Cassiope_palpebrata:20.944445,Cassiope_abbreviata:20.944445,Cassiope_fujianensis:20.944445,Cassiope_selaginoides:20.944445,Cassiope_membranifolia:20.944445,Cassiope_fastigiata:20.944445,Cassiope_myosuroides:20.944445,Cassiope_argyrotricha:20.944445,Cassiope_wardii:20.944445)Cassiope:20.944445,(Diplarche_multiflora:20.944445,Diplarche_pauciflora:20.944445)Diplarche:20.944445,(Rhododendron_pseudochrysanthum:20.944445,Rhododendron_proteoides:20.944445,Rhododendron_pumilum:20.944445,Rhododendron_amundsenianum:20.944445,Rhododendron_atropuniceum:20.944445,Rhododendron_balangense:20.944445,Rhododendron_leucaspis:20.944445,Rhododendron_mucronatum:20.944445,Rhododendron_callimorphum_var.myiagrum:20.944445,Rhododendron_taggianum:20.944445,Rhododendron_hongkongense:20.944445,Rhododendron_vellereum:20.944445,Rhododendron_balfourianum_var.aganniphoides:20.944445,Rhododendron_eudoxum_var.mesopolium:20.944445,Rhododendron_zaleucum:20.944445,Rhododendron_souliei:20.944445,Rhododendron_cretaceum:20.944445,Rhododendron_tsariense:20.944445,Rhododendron_liliiflorum:20.944445,Rhododendron_punctifolium:20.944445,Rhododendron_thomsonii:20.944445,Rhododendron_catacosmum:20.944445,Rhododendron_crassimedium:20.944445,Rhododendron_bracteatum:20.944445,Rhododendron_taronense:20.944445,Rhododendron_tenuilaminare:20.944445,Rhododendron_tenuifolium:20.944445,Rhododendron_leptothrium:20.944445,Rhododendron_moupinense:20.944445,Rhododendron_spinuliferum:20.944445,Rhododendron_pocophorum:20.944445,Rhododendron_dichroanthum_subsp.scyphocalyx:20.944445,Rhododendron_nivale_subsp.boreale:20.944445,Rhododendron_hypoblematosum:20.944445,Rhododendron_bijiangense:20.944445,Rhododendron_microphyton_var.trichanthum:20.944445,Rhododendron_calvescens:20.944445,Rhododendron_simiarum_var.versicolor:20.944445,Rhododendron_sanguineum_var.didymoides:20.944445,Rhododendron_pomense:20.944445,Rhododendron_hemsleyanum:20.944445,Rhododendron_griffithianum:20.944445,Rhododendron_insigne:20.944445,Rhododendron_tubiforme:20.944445,Rhododendron_dimitrium:20.944445,Rhododendron_charitopes_subsp.tsangpoense:20.944445,Rhododendron_oreogenum:20.944445,Rhododendron_principis:20.944445,Rhododendron_scabrifolium:20.944445,Rhododendron_meridionale_var.setistylum:20.944445,Rhododendron_fragariflorum:20.944445,Rhododendron_telmateium:20.944445,Rhododendron_lateriflorum:20.944445,Rhododendron_camelliiflorum:20.944445,Rhododendron_apricum:20.944445,Rhododendron_piercei:20.944445,Rhododendron_lepidostylum:20.944445,Rhododendron_citriniflorum:20.944445,Rhododendron_fletcherianum:20.944445,Rhododendron_hoi:20.944445,Rhododendron_traillianum:20.944445,Rhododendron_sparsifolium:20.944445,Rhododendron_sikangense:20.944445,Rhododendron_hookeri:20.944445,Rhododendron_unciferum:20.944445,Rhododendron_sperabile:20.944445,Rhododendron_chrysodoron:20.944445,Rhododendron_championae:20.944445,Rhododendron_beanianum:20.944445,Rhododendron_exasperatum:20.944445,Rhododendron_coeloneurum:20.944445,Rhododendron_habrotrichum:20.944445,Rhododendron_basilicum:20.944445,Rhododendron_crassistylum:20.944445,Rhododendron_wallichii:20.944445,Rhododendron_decorum:20.944445,Rhododendron_megacalyx:20.944445,Rhododendron_atrovirens:20.944445,Rhododendron_glanduliferum:20.944445,Rhododendron_lindleyi:20.944445,Rhododendron_excellens:20.944445,Rhododendron_taliense:20.944445,Rhododendron_huguangense:20.944445,Rhododendron_nigroglandulosum:20.944445,Rhododendron_taipaoense:20.944445,Rhododendron_protistum_var.giganteum:20.944445,Rhododendron_rex:20.944445,Rhododendron_tashiroi:20.944445,Rhododendron_gemmiferum:20.944445,Rhododendron_grande:20.944445,Rhododendron_faberi_subsp.prattii:20.944445,Rhododendron_faithae:20.944445,Rhododendron_ririei:20.944445,Rhododendron_danbaense:20.944445,Rhododendron_uniflorum:20.944445,Rhododendron_flavidum:20.944445,Rhododendron_lanatoides:20.944445,Rhododendron_oblancifolium:20.944445,Rhododendron_dawuense:20.944445,Rhododendron_nakotiltum:20.944445,Rhododendron_temenium:20.944445,Rhododendron_rufohirtum:20.944445,Rhododendron_coelicum:20.944445,Rhododendron_hancockii:20.944445,Rhododendron_valentinianum_var.oblongilobatum:20.944445,Rhododendron_euchroum:20.944445,Rhododendron_annae_subsp.laxiflorum:20.944445,Rhododendron_maddenii_subsp.crassum:20.944445,Rhododendron_aberconwayi:20.944445,Rhododendron_tingwuense:20.944445,Rhododendron_shanii:20.944445,Rhododendron_roxieanum_var.cucullatum:20.944445,Rhododendron_keleticum:20.944445,Rhododendron_brevipetiolatum:20.944445,Rhododendron_chamaethomsonii_var.chamaethauma:20.944445,Rhododendron_brachypodum:20.944445,Rhododendron_ochraceum_var.brevicarpum:20.944445,Rhododendron_brachyanthum:20.944445,Rhododendron_breviperulatum:20.944445,Rhododendron_brevinerve:20.944445,Rhododendron_microgynum:20.944445,Rhododendron_brevicaudatum:20.944445,Rhododendron_farinosum:20.944445,Rhododendron_kendrickii:20.944445,Rhododendron_selense:20.944445,Rhododendron_cavaleriei:20.944445,Rhododendron_hodgsonii:20.944445,Rhododendron_polylepis:20.944445,Rhododendron_polytrichum:20.944445,Rhododendron_stewartianum:20.944445,Rhododendron_rupicola:20.944445,Rhododendron_bathypyllum:20.944445,Rhododendron_polycladum:20.944445,Rhododendron_arizelum:20.944445,Rhododendron_ebianense:20.944445,Rhododendron_ochraceum:20.944445,Rhododendron_argyophyllum_var.omeiense:20.944445,Rhododendron_praeteritum:20.944445,Rhododendron_auriculatum:20.944445,Rhododendron_floribundum:20.944445,Rhododendron_thayerianum:20.944445,Rhododendron_fangchengense:20.944445,Rhododendron_hypoglaucum:20.944445,Rhododendron_selense_subsp.jucundum:20.944445,Rhododendron_hemitrichotum:20.944445,Rhododendron_hylaeum:20.944445,Rhododendron_×_duclouxii:20.944445,Rhododendron_temenium_var.dealbatum:20.944445,Rhododendron_oreodoxa_var.fargesii:20.944445,Rhododendron_arboreum_var.roseum:20.944445,Rhododendron_balfourianum:20.944445,Rhododendron_impeditum:20.944445,Rhododendron_baileyi:20.944445,Rhododendron_dendricola:20.944445,Rhododendron_preptum:20.944445,Rhododendron_potaninii:20.944445,Rhododendron_detersile:20.944445,Rhododendron_setiferum:20.944445,Rhododendron_setosum:20.944445,Rhododendron_lapponicum:20.944445,Rhododendron_decorum_subsp.diaprepes:20.944445,Rhododendron_coriaceum:20.944445,Rhododendron_kongboense:20.944445,Rhododendron_gongshanense:20.944445,Rhododendron_keysii:20.944445,Rhododendron_dumicola:20.944445,Rhododendron_nitidulum:20.944445,Rhododendron_coryanum:20.944445,Rhododendron_haofui:20.944445,Rhododendron_tanastylum:20.944445,Rhododendron_agastum_var.pennivenium:20.944445,Rhododendron_ludlowii:20.944445,Rhododendron_guangnanense:20.944445,Rhododendron_kwangsiense:20.944445,Rhododendron_fuchsiifolium:20.944445,Rhododendron_guizhouense:20.944445,Rhododendron_gologense:20.944445,Rhododendron_pingianum:20.944445,Rhododendron_hainanense:20.944445,Rhododendron_flumineum:20.944445,Rhododendron_henanense:20.944445,Rhododendron_wasonii:20.944445,Rhododendron_pseudociliipes:20.944445,Rhododendron_eudoxum_var.bruneifolium:20.944445,Rhododendron_sanguineum_var.didymum:20.944445,Rhododendron_meddianum:20.944445,Rhododendron_spanotrichum:20.944445,Rhododendron_irroratum_subsp.pogonostylum:20.944445,Rhododendron_vialii:20.944445,Rhododendron_glischrum_subsp.rude:20.944445,Rhododendron_chihsinianum:20.944445,Rhododendron_dalhousiae_var.rhabdotum:20.944445,Rhododendron_roseatum:20.944445,Rhododendron_sherriffii:20.944445,Rhododendron_rubiginosum:20.944445,Rhododendron_wightii:20.944445,Rhododendron_faucium:20.944445,Rhododendron_simiarum:20.944445,Rhododendron_pachyphyllum:20.944445,Rhododendron_hunanense:20.944445,Rhododendron_huadingense:20.944445,Rhododendron_eudoxum:20.944445,Rhododendron_sinonuttallii:20.944445,Rhododendron_complexum:20.944445,Rhododendron_wardii:20.944445,Rhododendron_kasoense:20.944445,Rhododendron_minyaense:20.944445,Rhododendron_temenium_var.gilvum:20.944445,Rhododendron_seinghkuense:20.944445,Rhododendron_xanthocodon:20.944445,Rhododendron_rufum:20.944445,Rhododendron_hunnewellianum_subsp.rockii:20.944445,Rhododendron_aganniphum_var.flavorufum:20.944445,Rhododendron_maculiferum_subsp.anhweiense:20.944445,Rhododendron_flavantherum:20.944445,Rhododendron_lanatum:20.944445,Rhododendron_genestierianum:20.944445,Rhododendron_hippophaeoides:20.944445,Rhododendron_tephropeplum:20.944445,Rhododendron_heliolepis_var.fumidum:20.944445,Rhododendron_huidongense:20.944445,Rhododendron_esetulosum:20.944445,Rhododendron_neriiflorum:20.944445,Rhododendron_pemakoense:20.944445,Rhododendron_rex_subsp.fictolacteum:20.944445,Rhododendron_calophytum_var.openshawianum:20.944445,Rhododendron_subflumineum:20.944445,Rhododendron_kiangsiense:20.944445,Rhododendron_rubiginosum_var.leclerei:20.944445,Rhododendron_ciliatum:20.944445,Rhododendron_ciliicalyx:20.944445,Rhododendron_clementinae_subsp.aureodorsale:20.944445,Rhododendron_faberi:20.944445,Rhododendron_calophytum_var.jingfuense:20.944445,Rhododendron_rupicola_var.chryseum:20.944445,Rhododendron_elegantulum:20.944445,Rhododendron_jinpingense:20.944445,Rhododendron_leptocladon:20.944445,Rhododendron_pilostylum:20.944445,Rhododendron_longipes_var.chienianum:20.944445,Rhododendron_jinxiuense:20.944445,Rhododendron_pulchrum:20.944445,Rhododendron_jinggangshanicum:20.944445,Rhododendron_circinnatum:20.944445,Rhododendron_roxieanum:20.944445,Rhododendron_haematodes_subsp.chaetomallum:20.944445,Rhododendron_invictum:20.944445,Rhododendron_westlandii:20.944445,Rhododendron_sperabiloides:20.944445,Rhododendron_rex_subsp.gratum:20.944445,Rhododendron_dichroanthum_subsp.apodectum:20.944445,Rhododendron_adenosum:20.944445,Rhododendron_sinofalconeri:20.944445,Rhododendron_rothschildii:20.944445,Rhododendron_eurysiphon:20.944445,Rhododendron_sphaeroblastum:20.944445,Rhododendron_beesianum:20.944445,Rhododendron_platypodum:20.944445,Rhododendron_labolengense:20.944445,Rhododendron_discolor:20.944445,Rhododendron_subcerinum:20.944445,Rhododendron_lukiangense:20.944445,Rhododendron_cyanocarpum:20.944445,Rhododendron_trilectorum:20.944445,Rhododendron_laojunshanense:20.944445,Rhododendron_leiboense:20.944445,Rhododendron_leishanicum:20.944445,Rhododendron_trichogynum:20.944445,Rhododendron_tatsienense_var.nudatum:20.944445,Rhododendron_litchiifolium:20.944445,Rhododendron_phaeochrysum:20.944445,Rhododendron_comisteum:20.944445,Rhododendron_fulvum:20.944445,Rhododendron_huangum:20.944445,Rhododendron_tsoi:20.944445,Rhododendron_dichroanthum:20.944445,Rhododendron_albertsenianum:20.944445,Rhododendron_heliolepis:20.944445,Rhododendron_microphyton:20.944445,Rhododendron_vernicosum:20.944445,Rhododendron_anthopogonoides:20.944445,Rhododendron_schistocalyx:20.944445,Rhododendron_×sinosimulans:20.944445,Rhododendron_aganniphum_var.schizopeplum:20.944445,Rhododendron_lanigerum:20.944445,Rhododendron_nyingchiense:20.944445,Rhododendron_trichostomum_var.radinum:20.944445,Rhododendron_lepidotum:20.944445,Rhododendron_rhombifolium:20.944445,Rhododendron_sulfureum:20.944445,Rhododendron_asperulum:20.944445,Rhododendron_virgatum:20.944445,Rhododendron_chunii:20.944445,Rhododendron_lungchiense:20.944445,Rhododendron_florulentum:20.944445,Rhododendron_dekatanum:20.944445,Rhododendron_przewalskii:20.944445,Rhododendron_dasycladoides:20.944445,Rhododendron_luhuoense:20.944445,Rhododendron_flavoflorum:20.944445,Rhododendron_lulangense:20.944445,Rhododendron_irroratum:20.944445,Rhododendron_callimorphum:20.944445,Rhododendron_detonsum:20.944445,Rhododendron_searsiae:20.944445,Rhododendron_brachyanthum_subsp.hypolepidotum:20.944445,Rhododendron_clementinae:20.944445,Rhododendron_maculiferum:20.944445,Rhododendron_malipoense:20.944445,Rhododendron_barkamense:20.944445,Rhododendron_maguanense:20.944445,Rhododendron_ovatum:20.944445,Rhododendron_delavayi:20.944445,Rhododendron_strigillosum:20.944445,Rhododendron_maoerense:20.944445,Rhododendron_dasypetalum:20.944445,Rhododendron_chamaethomsonii_var.chamaedoron:20.944445,Rhododendron_valentinianum:20.944445,Rhododendron_bainbridgeanum:20.944445,Rhododendron_laudandum:20.944445,Rhododendron_heliolepis_var.oporinum:20.944445,Rhododendron_seniavinii:20.944445,Rhododendron_poilanei:20.944445,Rhododendron_stamineum_var.lasiocarpum:20.944445,Rhododendron_cephalanthum:20.944445,Rhododendron_hypenanthum:20.944445,Rhododendron_lasiostylum:20.944445,Rhododendron_augustinii:20.944445,Rhododendron_pubicostatum:20.944445,Rhododendron_moulmainense:20.944445,Rhododendron_websterianum:20.944445,Rhododendron_zaleucum_var.pubifolium:20.944445,Rhododendron_radendum:20.944445,Rhododendron_selense_subsp.dasycladum:20.944445,Rhododendron_alutaceum_var.iodes:20.944445,Rhododendron_venator:20.944445,Rhododendron_rubiginosum_var.ptilostylum:20.944445,Rhododendron_delavayi_var.pilostylum:20.944445,Rhododendron_trichostomum:20.944445,Rhododendron_maowenense:20.944445,Rhododendron_calostrotum:20.944445,Rhododendron_campylocarpum_subsp.caloxanthum:20.944445,Rhododendron_calophytum:20.944445,Rhododendron_citriniflorum_var.horaeum:20.944445,Rhododendron_mengtszense:20.944445,Rhododendron_agastum:20.944445,Rhododendron_mainlingense:20.944445,Rhododendron_miyiense:20.944445,Rhododendron_densifolium:20.944445,Rhododendron_fastigiatum:20.944445,Rhododendron_sanguineum_var.himertum:20.944445,Rhododendron_populare:20.944445,Rhododendron_floccigerum:20.944445,Rhododendron_facetum:20.944445,Rhododendron_mianningense:20.944445,Rhododendron_hunnewellianum:20.944445,Rhododendron_montroseanum:20.944445,Rhododendron_medoense:20.944445,Rhododendron_nuttallii:20.944445,Rhododendron_nakaharai:20.944445,Rhododendron_meridionale:20.944445,Rhododendron_nanjianense:20.944445,Rhododendron_naamkwanense:20.944445,Rhododendron_levinei:20.944445,Rhododendron_nanpingense:20.944445,Rhododendron_glischrum:20.944445,Rhododendron_viscidum:20.944445,Rhododendron_erosum:20.944445,Rhododendron_phaeochrysum_var.agglutinatum:20.944445,Rhododendron_aureum:20.944445,Rhododendron_saluenense:20.944445,Rhododendron_parmulatum:20.944445,Rhododendron_vesiculiferum:20.944445,Rhododendron_edgeworthii:20.944445,Rhododendron_pronum:20.944445,Rhododendron_saluenense_var.prostratum:20.944445,Rhododendron_pingbianense:20.944445,Rhododendron_erastum:20.944445,Rhododendron_pugeense:20.944445,Rhododendron_paradoxum:20.944445,Rhododendron_thymifolium:20.944445,Rhododendron_polyraphidoideum:20.944445,Rhododendron_argyophyllum_var.nankingense:20.944445,Rhododendron_feddei:20.944445,Rhododendron_protistum:20.944445,Rhododendron_tsinlingense:20.944445,Rhododendron_qinghaiense:20.944445,Rhododendron_emarginatum:20.944445,Rhododendron_anthopogon:20.944445,Rhododendron_loniceraeflorum:20.944445,Rhododendron_pachytrichum:20.944445,Rhododendron_pubescens:20.944445,Rhododendron_mollicomum:20.944445,Rhododendron_igneum:20.944445,Rhododendron_lacteum:20.944445,Rhododendron_galactinum:20.944445,Rhododendron_papillatum:20.944445,Rhododendron_forrestii_subsp.papillatum:20.944445,Rhododendron_rhuyuenense:20.944445,Rhododendron_shweliense:20.944445,Rhododendron_triflorum:20.944445,Rhododendron_montigenum:20.944445,Rhododendron_oreodoxa:20.944445,Rhododendron_oreotrephes:20.944445,Rhododendron_taibaiense:20.944445,Rhododendron_martinianum:20.944445,Rhododendron_yungchangense:20.944445,Rhododendron_scopulorum:20.944445,Rhododendron_shimianense:20.944445,Rhododendron_haematodes:20.944445,Rhododendron_petrocharis:20.944445,Rhododendron_calophytum_var.pauciflorum:20.944445,Rhododendron_dignabile:20.944445,Rhododendron_hanceanum:20.944445,Rhododendron_changii:20.944445,Rhododendron_dendrocharis:20.944445,Rhododendron_arboreum:20.944445,Rhododendron_sargentianum:20.944445,Rhododendron_nymphaeoides:20.944445,Rhododendron_sutchuenense:20.944445,Rhododendron_jasminoides:20.944445,Rhododendron_spiciferum:20.944445,Rhododendron_rubropilosum:20.944445,Rhododendron_formosanum:20.944445,Rhododendron_purdomii:20.944445,Rhododendron_taishunense:20.944445,Rhododendron_pruniflorum:20.944445,Rhododendron_annae:20.944445,Rhododendron_diphrocalyx:20.944445,Rhododendron_tianlinense:20.944445,Rhododendron_myrsinifolium:20.944445,Rhododendron_viscidifolium:20.944445,Rhododendron_trichostomum_var.ledoides:20.944445,Rhododendron_capitatum:20.944445,Rhododendron_mitriforme:20.944445,Rhododendron_sinogrande:20.944445,Rhododendron_hirsutipetiolatum:20.944445,Rhododendron_pendulum:20.944445,Rhododendron_henryi_var.dunnii:20.944445,Rhododendron_declivatum:20.944445,Rhododendron_anthosphaerum:20.944445,Rhododendron_orbiculare:20.944445,Rhododendron_sanguineum_var.cloiophorum:20.944445,Rhododendron_campylocarpum:20.944445,Rhododendron_adenopodum:20.944445,Rhododendron_henryi:20.944445,Rhododendron_campylogynum:20.944445,Rhododendron_bulu:20.944445,Rhododendron_serotinum:20.944445,Rhododendron_neriiflorum_var.agetum:20.944445,Rhododendron_primulaeflorum_var.cephalanthoides:20.944445,Rhododendron_hyperythrum:20.944445,Rhododendron_sperabile_var.weihsiense:20.944445,Rhododendron_urophyllum:20.944445,Rhododendron_ambiguum:20.944445,Rhododendron_wasonii_var.wenchuanense:20.944445,Rhododendron_asterochnoum:20.944445,Rhododendron_wolongense:20.944445,Rhododendron_roxieoides:20.944445,Rhododendron_watsonii:20.944445,Rhododendron_hemsleyanum_var.chengianum:20.944445,Rhododendron_wumingense:20.944445,Rhododendron_megalanthum:20.944445,Rhododendron_niveum:20.944445,Rhododendron_xichangense:20.944445,Rhododendron_xiguense:20.944445,Rhododendron_latoucheae:20.944445,Rhododendron_oligocarpum:20.944445,Rhododendron_tenue:20.944445,Rhododendron_amandum:20.944445,Rhododendron_tapetiforme:20.944445,Rhododendron_delavayi_var.peramoenum:20.944445,Rhododendron_meridionale_var.minor:20.944445,Rhododendron_xanthostephanum:20.944445,Rhododendron_erythrocalyx:20.944445,Rhododendron_linearilobum:20.944445,Rhododendron_ramipilosum:20.944445,Rhododendron_roxieanum_var.oreonastes:20.944445,Rhododendron_crinigerum_var.euadenium:20.944445,Rhododendron_pocophorum_var.hemidartum:20.944445,Rhododendron_mitriforme_var.setaceum:20.944445,Rhododendron_bachii:20.944445,Rhododendron_adenogynum:20.944445,Rhododendron_meddianum_var.atrokermesinum:20.944445,Rhododendron_neriiflorum_var.approinquans:20.944445,Rhododendron_alutaceum_var.russotinctum:20.944445,Rhododendron_dichroanthum_subsp.septentrionale:20.944445,Rhododendron_davidii:20.944445,Rhododendron_adenanthum:20.944445,Rhododendron_leptopeplum:20.944445,Rhododendron_codonanthum:20.944445,Rhododendron_guizhongense:20.944445,Rhododendron_ciliipes:20.944445,Rhododendron_tutcherae:20.944445,Rhododendron_thomsonii_subsp.lopsangianum:20.944445,Rhododendron_minutiflorum:20.944445,Rhododendron_xiaoxidongense:20.944445,Rhododendron_calostrotum_var.calciphilum:20.944445,Rhododendron_decorum_subsp.parvistigmaticum:20.944445,Rhododendron_cuneatum:20.944445,Rhododendron_kyawi:20.944445,Rhododendron_fulgens:20.944445,Rhododendron_dauricum:20.944445,Rhododendron_pudorosum:20.944445,Rhododendron_aperantum:20.944445,Rhododendron_concinnum:20.944445,Rhododendron_bureavii:20.944445,Rhododendron_siderophyllum:20.944445,Rhododendron_nivale:20.944445,Rhododendron_riparioides:20.944445,Rhododendron_aganniphum:20.944445,Rhododendron_sanguineum:20.944445,Rhododendron_saxatile:20.944445,Rhododendron_charitopes:20.944445,Rhododendron_rupivalleculatum:20.944445,Rhododendron_miniatum:20.944445,Rhododendron_mallotum:20.944445,Rhododendron_yaogangxianense:20.944445,Rhododendron_yaoshanicum:20.944445,Rhododendron_racemosum:20.944445,Rhododendron_monanthum:20.944445,Rhododendron_yizhangense:20.944445,Rhododendron_leptocarpum:20.944445,Rhododendron_sidereum:20.944445,Rhododendron_argyrophyllum:20.944445,Rhododendron_maddenii:20.944445,Rhododendron_intricatum:20.944445,Rhododendron_primulaeflorum:20.944445,Rhododendron_cerasinum:20.944445,Rhododendron_barbatum:20.944445,Rhododendron_hirtipes:20.944445,Rhododendron_tatsienense:20.944445,Rhododendron_yungningense:20.944445,Rhododendron_praestans:20.944445,Rhododendron_mimetes:20.944445,Rhododendron_morii:20.944445,Rhododendron_semnoides:20.944445,Rhododendron_williamsianum:20.944445,Rhododendron_vaccinioides:20.944445,Rhododendron_fortunei:20.944445,Rhododendron_pachypodum:20.944445,Rhododendron_chamaethomsonii:20.944445,Rhododendron_eclecteum:20.944445,Rhododendron_praevernum:20.944445,Rhododendron_araiophyllum:20.944445,Rhododendron_phaeochrysum_var.levistratum:20.944445,Rhododendron_augustinii_subsp.chasmanthum:20.944445,Rhododendron_longipes:20.944445,Rhododendron_eclecteum_var.bellatulum:20.944445,Rhododendron_crinigerum:20.944445,Rhododendron_longicalyx:20.944445,Rhododendron_calvescens_var.duseimatum:20.944445,Rhododendron_tubulosum:20.944445,Rhododendron_longifalcatum:20.944445,Rhododendron_longesquamatum:20.944445,Rhododendron_longiperulatum:20.944445,Rhododendron_trichanthum:20.944445,Rhododendron_stamineum:20.944445,Rhododendron_dalhousiae:20.944445,Rhododendron_ramsdenianum:20.944445,Rhododendron_longistylum:20.944445,Rhododendron_hippophaeoides_var.occidentale:20.944445,Rhododendron_ciliicalyx_subsp.lyi:20.944445,Rhododendron_orthocladum_var.longistylum:20.944445,Rhododendron_megeratum:20.944445,Rhododendron_tsaii:20.944445,Rhododendron_micranthum:20.944445,Rhododendron_bonvalotii:20.944445,Rhododendron_auritum:20.944445,Rhododendron_spadiceum:20.944445,Rhododendron_zheguense:20.944445,Rhododendron_kawakamii:20.944445,Rhododendron_orthocladum:20.944445,Rhododendron_zhongdianense:20.944445,Rhododendron_campanulatum:20.944445,Rhododendron_wiltonii:20.944445,Rhododendron_denudatum:20.944445,Rhododendron_griersonianum:20.944445,Rhododendron_cinnabarinum:20.944445,Rhododendron_oldhamii:20.944445,Rhododendron_joniense:20.944445,Rhododendron_ziyuanense:20.944445,Rhododendron_flosculum:20.944445,Rhododendron_strigillosum_var.monosematum:20.944445,Rhododendron_forrestii:20.944445,Rhododendron_amesiae:20.944445,Rhododendron_russatum:20.944445,Rhododendron_naamkwanense_var.cryptonerve:20.944445,Rhododendron_sanguineum_var.haemaleum:20.944445,Rhododendron_uvarifolium:20.944445,Rhododendron_traillianum_var.dictyotum:20.944445,Rhododendron_alutaceum:20.944445,Rhododendron_fuscipilum:20.944445)Rhododendron:20.944445,(Leucothoe_griffithiana:20.944445,Leucothoe_tonkinensis:20.944445)Leucothoe:20.944445,(Empetrum_nigrum_var.japonicum:20.944445)Empetrum:20.944445,(Gaultheria_leucocarpa:20.944445,Gaultheria_sinensis_var.nivea:20.944445,Gaultheria_cardiosepala:20.944445,Gaultheria_praticola:20.944445,Gaultheria_straminea:20.944445,Gaultheria_trichophylla:20.944445,Gaultheria_dumicola:20.944445,Gaultheria_dumicola_var.aspera:20.944445,Gaultheria_leucocarpa_var.yunnanensis:20.944445,Gaultheria_brevistipes:20.944445,Gaultheria_notabilis:20.944445,Gaultheria_fragrantissima:20.944445,Gaultheria_suborbicularis:20.944445,Gaultheria_borneensis:20.944445,Gaultheria_dumicola_var.petanoneuron:20.944445,Gaultheria_hookeri:20.944445,Gaultheria_sinensis:20.944445,Gaultheria_pseudonotabilis:20.944445,Gaultheria_pyroloides:20.944445,Gaultheria_hypochlora:20.944445,Gaultheria_leucocarpa_var.crenulata:20.944445,Gaultheria_prostrata:20.944445,Gaultheria_cuneata:20.944445,Gaultheria_tetramera:20.944445,Gaultheria_nummularioides:20.944445,Gaultheria_griffithiana:20.944445,Gaultheria_semi-infera:20.944445,Gaultheria_wardii:20.944445,Gaultheria_hookeri_var.angustifolia:20.944445,Gaultheria_dolichopoda:20.944445,Gaultheria_longiracemosa:20.944445)Gaultheria:20.944445)ericaceae:20.944443):20.944447,((Clethra_petelotii:27.925926,Clethra_bodinieri:27.925926)Clethra:27.925926)clethraceae:27.925926):20.944443,((((Actinidia_fulvicoma_var.hirsuta:20.944445,Actinidia_farinosa:20.944445,Actinidia_rubricaulis_var.coriacea:20.944445,Actinidia_rubricaulis:20.944445,Actinidia_rufotricha:20.944445,Actinidia_fulvicoma_var.pachyphylla:20.944445,Actinidia_fulvicoma:20.944445,Actinidia_fulvicoma_var.cinerascens:20.944445,Actinidia_liangguangensis:20.944445,Actinidia_melliana:20.944445,Actinidia_rufotricha_var.glomerata:20.944445,Actinidia_cylindrica_var.reticulata:20.944445,Actinidia_cylindrica:20.944445)Actinidia:20.944445,(Saurauia_erythrocarpa_var.grosseserrata:20.944445,Saurauia_punduana:20.944445,Saurauia_rubricalyx:20.944445,Saurauia_erythrocarpa:20.944445,Saurauia_cerea:20.944445,Saurauia_griffithii:20.944445,Saurauia_napaulensis:20.944445,Saurauia_polyneura_var.paucinervis:20.944445,Saurauia_tristyla:20.944445,Saurauia_griffithii_var.annamica:20.944445,Saurauia_yunnanensis:20.944445,Saurauia_miniata:20.944445)Saurauia:20.944445)actinidiaceae:20.944443):20.944447):20.944443):20.944443,((Pyrenaria_hirta:41.888889,Pyrenaria_spectabilis:41.888889,Pyrenaria_jonquieriana_subsp.multisepala:41.888889,Pyrenaria_kwangsiensis:41.888889,Pyrenaria_microcarpa_var.ovalifolia:41.888889,Pyrenaria_menglaensis:41.888889,Pyrenaria_pingpienensis:41.888889,Pyrenaria_microcarpa:41.888889,Pyrenaria_diospyricarpa:41.888889,Pyrenaria_khasiana:41.888889,Pyrenaria_sophiae:41.888889,Pyrenaria_wuana:41.888889,Pyrenaria_oblongicarpa:41.888889,Pyrenaria_spectabilis_var.greeniae:41.888889)Pyrenaria:41.888889,(Adinandra_howii:41.888889,Adinandra_bockiana:41.888889,Adinandra_hirta:41.888889,Adinandra_glischroloma_var.macrosepala:41.888889,Adinandra_grandis:41.888889,Adinandra_megaphylla:41.888889,Adinandra_hainanensis:41.888889,Adinandra_bockiana_var.acutifolia:41.888889,Adinandra_latifolia:41.888889,Adinandra_glischroloma:41.888889,Adinandra_nitida:41.888889,Adinandra_lasiostyla:41.888889,Adinandra_pingbianensis:41.888889,Adinandra_formosana:41.888889,Adinandra_epunctata:41.888889,Adinandra_filipes:41.888889,Adinandra_lancipetala:41.888889,Adinandra_nigroglandulosa:41.888889,Adinandra_millettii:41.888889,Adinandra_elegans:41.888889,Adinandra_glischroloma_var.jubata:41.888889)Adinandra:41.888889,(Anneslea_fragrans:41.888889)Anneslea:41.888889,(Stewartia_pteropetiolata:41.888889,Stewartia_obovata:41.888889,Stewartia_villosa_var.kwangtungensis:41.888889,Stewartia_crassifolia:41.888889,Stewartia_sinii:41.888889,Stewartia_laotica:41.888889,Stewartia_medogensis:41.888889,Stewartia_villosa:41.888889,Stewartia_sinensis_var.shensiensis:41.888889,Stewartia_sichuanensis:41.888889,Stewartia_densivillosa:41.888889,Stewartia_micrantha:41.888889,Stewartia_cordifolia:41.888889,Stewartia_calcicola:41.888889)Stewartia:41.888889,(Apterosperma_oblata:41.888889)Apterosperma:41.888889,(Gordonia_axillaris:41.888889,Gordonia_hainanensis:41.888889,Gordonia_chrysandra:41.888889,Gordonia_speciosa:41.888889,Gordonia_longicarpa:41.888889)Gordonia:41.888889,(Cleyera_incornuta:41.888889,Cleyera_lipingensis:41.888889,Cleyera_japonica_var.wallichiana:41.888889,Cleyera_obovata:41.888889,Cleyera_japonica:41.888889,Cleyera_pachyphylla:41.888889,Cleyera_parvifolia:41.888889,Cleyera_yangchunensis:41.888889,Cleyera_obscurinervis:41.888889,Cleyera_longicarpa:41.888889)Cleyera:41.888889,(Ternstroemia_insignis:41.888889,Ternstroemia_hainanensis:41.888889,Ternstroemia_gymnanthera:41.888889,Ternstroemia_kwangtungensis:41.888889,Ternstroemia_luteoflora:41.888889,Ternstroemia_biangulipes:41.888889,Ternstroemia_gymnanthera_var.wightii:41.888889,Ternstroemia_nitida:41.888889,Ternstroemia_japonica:41.888889,Ternstroemia_simaoensis:41.888889,Ternstroemia_sichuanensis:41.888889,Ternstroemia_microphylla:41.888889,Ternstroemia_yunnanensis:41.888889,Ternstroemia_conicocarpa:41.888889)Ternstroemia:41.888889,(Eurya_impressinervis:41.888889,Eurya_semiserrata:41.888889,Eurya_leptophylla:41.888889,Eurya_emarginata:41.888889,Eurya_alata:41.888889,Eurya_fangii_var.fangii:41.888889,Eurya_acuminoides:41.888889,Eurya_amplexifolia:41.888889,Eurya_metcalfiana:41.888889,Eurya_glandulosa_var.dasyclados:41.888889,Eurya_chukiangensis:41.888889,Eurya_megatrichocarpa:41.888889,Eurya_magniflora:41.888889,Eurya_fangii_var.megaphylla:41.888889,Eurya_quinquelocularis:41.888889,Eurya_weissiae:41.888889,Eurya_paratetragonoclada:41.888889,Eurya_brevistyla:41.888889,Eurya_crenatifolia:41.888889,Eurya_obtusifolia:41.888889,Eurya_polyneura:41.888889,Eurya_hupehensis:41.888889,Eurya_auriformis:41.888889,Eurya_distichophylla:41.888889,Eurya_groffii:41.888889,Eurya_muricata:41.888889,Eurya_gungshanensis:41.888889,Eurya_glaberrima:41.888889,Eurya_chinensis_var.glabra:41.888889,Eurya_cuneata_var.glabra:41.888889,Eurya_kueichowensis:41.888889,Eurya_hainanensis:41.888889,Eurya_pittosporifolia:41.888889,Eurya_macartneyi:41.888889,Eurya_rubiginosa:41.888889,Eurya_crassilimba:41.888889,Eurya_ciliata:41.888889,Eurya_gnaphalocarpa:41.888889,Eurya_pyracanthifolia:41.888889,Eurya_perserrata:41.888889,Eurya_acutisepala:41.888889,Eurya_acuminata:41.888889,Eurya_acuminatissima:41.888889,Eurya_persicaefolia:41.888889,Eurya_obtusifolia_var.aurea:41.888889,Eurya_loquaiana_var.aureo-punctata:41.888889,Eurya_jintungensis:41.888889,Eurya_oblonga_var.oblonga:41.888889,Eurya_handel-mazzettii:41.888889,Eurya_rengechiensis:41.888889,Eurya_japonica:41.888889,Eurya_lunglingensis:41.888889,Eurya_ovatifolia:41.888889,Eurya_marlipoensis:41.888889,Eurya_trichocarpa:41.888889,Eurya_stenophylla_var.pubescens:41.888889,Eurya_muricata_var.huiana:41.888889,Eurya_chinensis:41.888889,Eurya_tsaii:41.888889,Eurya_lanciformis:41.888889,Eurya_henryi:41.888889,Eurya_inaequalis:41.888889,Eurya_bifidostyla:41.888889,Eurya_tetragonoclada:41.888889,Eurya_strigillosa:41.888889,Eurya_prunifolia:41.888889,Eurya_disticha:41.888889,Eurya_hebeclados:41.888889,Eurya_pentagyna:41.888889,Eurya_nitida:41.888889,Eurya_loquaiana:41.888889,Eurya_glandulosa:41.888889,Eurya_pseudocerasifera:41.888889,Eurya_glandulosa_var.cuneiformis:41.888889,Eurya_cuneata:41.888889,Eurya_obliquifolia:41.888889,Eurya_velutina:41.888889,Eurya_saxicola:41.888889,Eurya_cavinervis_f.cavinervis:41.888889,Eurya_rubiginosa_var.attenuata:41.888889,Eurya_stenophylla:41.888889,Eurya_patentipila:41.888889,Eurya_stenophylla_var.caudata:41.888889,Eurya_rugosa:41.888889)Eurya:41.888889,(Euryodendron_excelsum:41.888889)Euryodendron:41.888889,(Schima_sericans_var.paracrenata:41.888889,Schima_brevipedicellata:41.888889,Schima_crenata:41.888889,Schima_multibracteata:41.888889,Schima_sericans:41.888889,Schima_wallichii:41.888889,Schima_sinensis:41.888889,Schima_villosa:41.888889,Schima_superba:41.888889,Schima_noronhae:41.888889,Schima_remotiserrata:41.888889,Schima_parviflora:41.888889,Schima_argentea:41.888889,Schima_khasiana:41.888889)Schima:41.888889,(Camellia_anlungensis:41.888889,Camellia_impressinervis:41.888889,Camellia_sinensis_var.pubilimba:41.888889,Camellia_candida:41.888889,Camellia_szemaoensis:41.888889,Camellia_punctata:41.888889,Camellia_chrysanthoides:41.888889,Camellia_amplexifolia:41.888889,Camellia_furfuracea:41.888889,Camellia_sinensis:41.888889,Camellia_longissima:41.888889,Camellia_synaptica:41.888889,Camellia_rosthorniana:41.888889,Camellia_granthamiana:41.888889,Camellia_grandibracteata:41.888889,Camellia_tachangensis:41.888889,Camellia_assimiloides:41.888889,Camellia_semiserrata_var.magnocarpa:41.888889,Camellia_cuspidata_var.grandiflora:41.888889,Camellia_fluviatilis_var.megalantha:41.888889,Camellia_taliensis:41.888889,Camellia_kissi_var.confusa:41.888889,Camellia_flavida:41.888889,Camellia_sinensis_var.dehungensis:41.888889,Camellia_wardii:41.888889,Camellia_pachyandra:41.888889,Camellia_cupiformis:41.888889,Camellia_mairei_var.velutina:41.888889,Camellia_reticulata:41.888889,Camellia_edithae:41.888889,Camellia_indochinensis_var.tunghinensis:41.888889,Camellia_ilicifolia:41.888889,Camellia_azalea:41.888889,Camellia_brevistyla:41.888889,Camellia_pitardii_var.compressa:41.888889,Camellia_polyodonta:41.888889,Camellia_fangchengensis:41.888889,Camellia_crassicolumna_var.multiplex:41.888889,Camellia_cordifolia_var.glabrisepala:41.888889,Camellia_melliana:41.888889,Camellia_kwangsiensis:41.888889,Camellia_huana:41.888889,Camellia_costei:41.888889,Camellia_hekouensis:41.888889,Camellia_pyxidiacea_var.rubituberculata:41.888889,Camellia_crapnelliana:41.888889,Camellia_yunnanensis:41.888889,Camellia_crassicolumna:41.888889,Camellia_xanthochroma:41.888889,Camellia_anlungensis_var.acutiperulata:41.888889,Camellia_forrestii_var.acutisepala:41.888889,Camellia_petelotii:41.888889,Camellia_furfuracea_var.latipetiolate:41.888889,Camellia_cuspidata:41.888889,Camellia_euryoides:41.888889,Camellia_tuberculata:41.888889,Camellia_salicifolia:41.888889,Camellia_kissi:41.888889,Camellia_viridicalyx:41.888889,Camellia_pubipetala:41.888889,Camellia_pubifurfuracea:41.888889,Camellia_wardii_var.muricatula:41.888889,Camellia_kwangsiensis_var.kwangsiensis:41.888889,Camellia_transarisanensis:41.888889,Camellia_tsingpienensis_var.pubisepala:41.888889,Camellia_yunnanensis_var.camellioides:41.888889,Camellia_fraterna:41.888889,Camellia_synaptica_var.parviovata:41.888889,Camellia_euryoides_var.nokoensis:41.888889,Camellia_mairei:41.888889,Camellia_cuspidata_var.trichandra:41.888889,Camellia_ptilophylla:41.888889,Camellia_trichoclada:41.888889,Camellia_pilosperma:41.888889,Camellia_mileensis:41.888889,Camellia_leptophylla:41.888889,Camellia_semiserrata:41.888889,Camellia_indochinensis:41.888889,Camellia_saluenensis:41.888889,Camellia_pinggaoensis_var.pinggaoensis:41.888889,Camellia_tsingpienensis:41.888889,Camellia_sinensis_var.assamica:41.888889,Camellia_subintegra:41.888889,Camellia_pyxidiacea:41.888889,Camellia_japonica:41.888889,Camellia_mairei_var.lapidea:41.888889,Camellia_szechuanensis:41.888889,Camellia_lawii:41.888889,Camellia_lutchuensis:41.888889,Camellia_gymnogyna:41.888889,Camellia_glabricostata:41.888889,Camellia_costata:41.888889,Camellia_stuartiana:41.888889,Camellia_pitardii:41.888889,Camellia_parviflora:41.888889,Camellia_ilicifolia_var.neriifolia:41.888889,Camellia_gracilipes:41.888889,Camellia_euphlebia:41.888889,Camellia_viridicalyx_var.linearifolia:41.888889,Camellia_hongkongensis:41.888889,Camellia_tenii:41.888889,Camellia_villicarpa:41.888889,Camellia_micrantha:41.888889,Camellia_luteoflora:41.888889,Camellia_parvimuricata:41.888889,Camellia_grijsii_var.shensiensis:41.888889,Camellia_mileensis_var.microphylla:41.888889,Camellia_rhytidocarpa_var.microphylla:41.888889,Camellia_caudata_var.gracilis:41.888889,Camellia_cordifolia:41.888889,Camellia_gaudichaudii:41.888889,Camellia_oleifera:41.888889,Camellia_drupifera:41.888889,Camellia_fascicularis:41.888889,Camellia_forrestii:41.888889,Camellia_tsaii:41.888889,Camellia_fluviatilis:41.888889,Camellia_grijsii:41.888889,Camellia_longicalyx:41.888889,Camellia_lingipedicellata:41.888889,Camellia_elongata:41.888889,Camellia_polyodonta_var.longicaudata:41.888889,Camellia_caudata:41.888889,Camellia_cuspidata_var.chekiangensis:41.888889,Camellia_chekiangoleosa:41.888889,Camellia_gilbertii:41.888889,Camellia_rhytidocarpa:41.888889)Camellia:41.888889)theaceae:41.888885,((((Styrax_macrocarpus:25.133333,Styrax_grandiflorus:25.133333,Styrax_benzoinoides:25.133333,Styrax_roseus:25.133333,Styrax_hainanensis:25.133333,Styrax_confusus_var.superbus:25.133333,Styrax_chrysocarpus:25.133333,Styrax_agrestis:25.133333,Styrax_macranthus:25.133333,Styrax_argentifolius:25.133333,Styrax_zhejiangensis:25.133333,Styrax_chinensis:25.133333)Styrax:25.133333,(Parastyrax_macrophyllus:25.133333,Parastyrax_lacei:25.133333)Parastyrax:25.133333)styracaceae:25.133335):25.133331,((Symplocos_anomala:33.511112,Symplocos_poilanei:33.511112,Symplocos_ovatilobata:33.511112,Symplocos_hookeri:33.511112,Symplocos_pendula:33.511112,Symplocos_ramosissima:33.511112,Symplocos_fukienensis:33.511112,Symplocos_atriolivacea:33.511112,Symplocos_sulcata:33.511112,Symplocos_lucida:33.511112,Symplocos_lancifolia:33.511112,Symplocos_hainanensis:33.511112,Symplocos_heishanensis:33.511112,Symplocos_crassilimba:33.511112,Symplocos_cochinchinensis_subsp.Laurina:33.511112,Symplocos_dryophila:33.511112,Symplocos_ulotricha:33.511112,Symplocos_stellaris:33.511112,Symplocos_pyrifolia:33.511112,Symplocos_euryoides:33.511112,Symplocos_spectabilis:33.511112,Symplocos_groffii:33.511112,Symplocos_congesta:33.511112,Symplocos_austrosinensis:33.511112,Symplocos_pendula_var.hirtistylis:33.511112,Symplocos_nokoensis:33.511112,Symplocos_hookeri_var.tomentosa:33.511112,Symplocos_fordii:33.511112,Symplocos_sumuntia:33.511112,Symplocos_paucinervia:33.511112,Symplocos_pseudobarberina:33.511112,Symplocos_stellaris_var.aenea:33.511112,Symplocos_glomerata:33.511112,Symplocos_wikstroemiifolia:33.511112,Symplocos_glauca_var.epapillata:33.511112,Symplocos_cochinchinensis_var.angustifolia:33.511112,Symplocos_adenopus:33.511112,Symplocos_adenophylla:33.511112,Symplocos_glandulifera:33.511112,Symplocos_glauca:33.511112,Symplocos_cochinchinensis:33.511112,Symplocos_modesta:33.511112,Symplocos_dolichotricha:33.511112,Symplocos_racemosa:33.511112)Symplocos:33.511112)symplocaceae:33.511108):25.133331):20.944450,((((Rapanea_linearis:29.322224,Rapanea_cicatricosa:29.322224,Rapanea_kwangsiensis:29.322224,Rapanea_verruculosa:29.322224,Rapanea_neriifolia:29.322224,Rapanea_affinis:29.322224,Rapanea_faberi:29.322224)Rapanea:29.322224,(Ardisia_humilis:29.322224,Ardisia_brunnescens:29.322224,Ardisia_merrillii:29.322224,Ardisia_crispa:29.322224,Ardisia_baotingensis:29.322224,Ardisia_dasyrhizomatica:29.322224,Ardisia_crassinervosa:29.322224,Ardisia_hanceana:29.322224,Ardisia_elliptica:29.322224,Ardisia_sieboldii:29.322224,Ardisia_aberrans:29.322224,Ardisia_omissa:29.322224,Ardisia_mamillata:29.322224,Ardisia_perreticulata:29.322224,Ardisia_fordii:29.322224,Ardisia_ensifolia:29.322224,Ardisia_violacea:29.322224,Ardisia_brevicaulis:29.322224,Ardisia_pusilla:29.322224,Ardisia_replicata:29.322224,Ardisia_olivacea:29.322224,Ardisia_primulaefolia:29.322224,Ardisia_hypargyrea:29.322224,Ardisia_ordinata:29.322224,Ardisia_quinquegona:29.322224,Ardisia_malipoensis:29.322224,Ardisia_pubivenula:29.322224,Ardisia_densilepidotula:29.322224,Ardisia_thyrsiflora:29.322224,Ardisia_virens:29.322224,Ardisia_carnosicaulis:29.322224,Ardisia_shweliensis:29.322224,Ardisia_corymbifera:29.322224,Ardisia_conspersa:29.322224,Ardisia_lindleyana:29.322224,Ardisia_alyxiaefolia:29.322224,Ardisia_balansana:29.322224,Ardisia_solanacea:29.322224,Ardisia_ramondiiformis:29.322224,Ardisia_scalarinervis:29.322224,Ardisia_obtusa:29.322224,Ardisia_retroflexa:29.322224,Ardisia_caudata:29.322224,Ardisia_argenticaulis:29.322224,Ardisia_porifera:29.322224,Ardisia_sinoaustralis:29.322224,Ardisia_filiformis:29.322224,Ardisia_alutacea:29.322224,Ardisia_cornudentata:29.322224,Ardisia_garrettii:29.322224,Ardisia_chinensis:29.322224,Ardisia_maclurei:29.322224,Ardisia_nigropilosa:29.322224,Ardisia_villosa:29.322224,Ardisia_faberi:29.322224,Ardisia_waitakii:29.322224,Ardisia_verbascifolia:29.322224,Ardisia_pingbienensis:29.322224,Ardisia_curvula:29.322224,Ardisia_crenata:29.322224,Ardisia_japonica:29.322224,Ardisia_purpureovillosa:29.322224,Ardisia_gigantifolia:29.322224)Ardisia:29.322224,(Myrsine_stolonifera:29.322224,Myrsine_elliptica:29.322224,Myrsine_africana:29.322224,Myrsine_semiserrata:29.322224)Myrsine:29.322224,(Embelia_ribes:29.322224,Embelia_parviflora:29.322224,Embelia_sessiliflora:29.322224,Embelia_floribunda:29.322224,Embelia_ribes_var.pachyphylla:29.322224,Embelia_scandens:29.322224,Embelia_polypodioides:29.322224,Embelia_henryi:29.322224,Embelia_vestita:29.322224,Embelia_undulata:29.322224,Embelia_procumbens:29.322224,Embelia_carnosisperma:29.322224,Embelia_laeta:29.322224,Embelia_gamblei:29.322224)Embelia:29.322224,(Maesa_cavinervis:29.322224,Maesa_indica:29.322224,Maesa_macilentoides:29.322224,Maesa_ramentacea:29.322224,Maesa_japonica:29.322224,Maesa_brevipaniculata:29.322224,Maesa_hupehensis:29.322224,Maesa_densistriata:29.322224,Maesa_perlarius:29.322224,Maesa_ambigua:29.322224,Maesa_montana:29.322224,Maesa_salicifolia:29.322224,Maesa_permollis:29.322224,Maesa_marionae:29.322224,Maesa_insignis:29.322224,Maesa_acuminatissima:29.322224,Maesa_chisia:29.322224,Maesa_consanguinea:29.322224,Maesa_reticulata:29.322224,Maesa_striata_var.opaca:29.322224,Maesa_confusa:29.322224,Maesa_macilenta:29.322224,Maesa_membranacea:29.322224,Maesa_parvifolia:29.322224,Maesa_argentea:29.322224,Maesa_manipurensis:29.322224,Maesa_rugosa:29.322224)Maesa:29.322224)primulaceae:29.322220,((Diospyros_xishuangbannaensis:29.322222,Diospyros_balfouriana:29.322222,Diospyros_kerrii:29.322222,Diospyros_unisemina:29.322222,Diospyros_punctilimba:29.322222,Diospyros_fanjingshanica:29.322222,Diospyros_diversilimba:29.322222,Diospyros_esquirolii:29.322222,Diospyros_susarticulata:29.322222,Diospyros_maritima:29.322222,Diospyros_hainanensis:29.322222,Diospyros_hasseltii:29.322222,Diospyros_nigrocortex:29.322222,Diospyros_nitida:29.322222,Diospyros_ehretioides:29.322222,Diospyros_kotoensis:29.322222,Diospyros_oliviformis:29.322222,Diospyros_tutcheri:29.322222,Diospyros_hexamera:29.322222,Diospyros_longshengensis:29.322222,Diospyros_morrisiana:29.322222,Diospyros_strigosa:29.322222,Diospyros_caloneura:29.322222,Diospyros_miaoshanica:29.322222,Diospyros_inflata:29.322222,Diospyros_armata:29.322222,Diospyros_rubra:29.322222,Diospyros_maclurei:29.322222,Diospyros_howii:29.322222,Diospyros_siderophylla:29.322222,Diospyros_saxicola:29.322222,Diospyros_forrestii:29.322222,Diospyros_reticulinervis:29.322222,Diospyros_eriantha:29.322222,Diospyros_cathayensis:29.322222,Diospyros_reticulinervis_var.glabrescens:29.322222,Diospyros_corallina:29.322222,Diospyros_sichourensis:29.322222,Diospyros_xiangguiensis:29.322222,Diospyros_ferrea:29.322222,Diospyros_vaccinioides:29.322222,Diospyros_sunyiensis:29.322222,Diospyros_chunii:29.322222,Diospyros_tsangii:29.322222,Diospyros_dumetorum:29.322222,Diospyros_anisocalyx:29.322222,Diospyros_philippensis:29.322222,Diospyros_yunnanensis:29.322222,Diospyros_longibracteata:29.322222,Diospyros_zhenfengensis:29.322222)Diospyros:29.322222)ebenaceae:29.322224):29.322227,((Diploknema_butyracea:39.096298,Diploknema_yunnanensis:39.096298)Diploknema:39.096298,(Chrysophyllum_lanceolatum_var.stellatocarpon:39.096298)Chrysophyllum:39.096298,(Eberhardtia_tonkinensis:39.096298,Eberhardtia_aurata:39.096298)Eberhardtia:39.096298,(Sarcosperma_arboreum:39.096298,Sarcosperma_kachinense_var.simondii:39.096298,Sarcosperma_kachinense:39.096298,Sarcosperma_laurinum:39.096298,Sarcosperma_griffithii:39.096298)Sarcosperma:39.096298,(Pouteria_grandifolia:39.096298,Pouteria_annamensis:39.096298)Pouteria:39.096298,(Sinosideroxylon_yunnanense:39.096298,Sinosideroxylon_wightianum:39.096298,Sinosideroxylon_pedunculatum_var.pubifolium:39.096298,Sinosideroxylon_pedunculatum:39.096298)Sinosideroxylon:39.096298,(Manilkara_hexandra:39.096298)Manilkara:39.096298,(Palaquium_formosanum:39.096298)Palaquium:39.096298,(Xantolis_stenosepala:39.096298,Xantolis_stenosepala_var.brevistylis:39.096298,Xantolis_boniana_var.rostrata:39.096298,Xantolis_longispinosa:39.096298,Xantolis_shweliensis:39.096298)Xantolis:39.096298,(Planchonella_obovata:39.096298,Planchonella_clemensii:39.096298)Planchonella:39.096298,(Madhuca_hainanensis:39.096298,Madhuca_pasquieri:39.096298)Madhuca:39.096298)sapotaceae:39.096298):29.322220,(((Sladenia_celastrifolia:36.652779)Sladenia:36.652779)sladeniaceae:36.652779,((Pentaphylax_euryoides:36.652779)Pentaphylax:36.652779)pentaphylacaceae:36.652779):36.652779):20.944443,((Barringtonia_asiatica:55.851852,Barringtonia_fusicarpa:55.851852,Barringtonia_racemosa:55.851852)Barringtonia:55.851852)lecythidaceae:55.851852):20.944443)ericales:20.944443)ericales_to_asterales:7.222229,((((Swida_oblonga_var.griffithii:43.333336,Swida_oblonga_var.glabrescens:43.333336,Swida_oblonga:43.333336)Swida:43.333336,(Helwingia_japonica_var.hypoleuca:43.333336,Helwingia_chinensis_var.Crenata:43.333336,Helwingia_omeiensis:43.333336,Helwingia_japonica_var.zhejiangensis:43.333336,Helwingia_chinensis:43.333336)Helwingia:43.333336,(Mastixia_pentandra_subsp.cambodiana:43.333336)Mastixia:43.333336,(Aucuba_albo-punctifolia:43.333336,Aucuba_robusta:43.333336,Aucuba_himalaica_var.oblanceolata:43.333336,Aucuba_japonica_var.variegata:43.333336,Aucuba_confertiflora:43.333336,Aucuba_himalaica_var.pilosissima:43.333336,Aucuba_eriobotryaefolia:43.333336,Aucuba_japonica:43.333336,Aucuba_filicauda_var.pauciflora:43.333336,Aucuba_chinensis_subsp.chinensis:43.333336,Aucuba_himalaica:43.333336,Aucuba_chlorascens:43.333336,Aucuba_chinensis_var.angusta:43.333336,Aucuba_filicauda:43.333336,Aucuba_albo-punctifolia_var.angustula:43.333336,Aucuba_himalaica_var.dolichophylla:43.333336)Aucuba:43.333336,(Dendrobenthamia_hongkongensis_subsp.gigantea:43.333336,Dendrobenthamia_hongkongensis_subsp.tonkinensis:43.333336,Dendrobenthamia_hongkongensis_var.ferruginea:43.333336,Dendrobenthamia_hongkongensis_subsp.melanotricha:43.333336,Dendrobenthamia_elliptica:43.333336,Dendrobenthamia_capitata_var.capitata:43.333336,Dendrobenthamia_hongkongensis:43.333336,Dendrobenthamia_hongkongensis_subsp.elegans:43.333336)Dendrobenthamia:43.333336)cornaceae:43.333328):43.333344)cornales:43.333328)asterids:7.222214,((((((((Ceratoides_latens:37.314816)Ceratoides:37.314816,(Cladostachys_frutescens:37.314816)Cladostachys:37.314816)amaranthaceae:37.314812):37.314819,(((((((((Commicarpus_lantsangensis:14.925925)Commicarpus:14.925925,(Pisonia_grandis:14.925925,Pisonia_aculeata:14.925925)Pisonia:14.925925)nyctaginaceae:14.925928):14.925922):14.925930):14.925926):14.925926):14.925919):14.925926):14.925934):14.925919):14.925934):14.925919):14.925934,(((((Ceratostigma_griffithii:34.827160)Ceratostigma:34.827160,(Plumbago_zeylanica:34.827160)Plumbago:34.827160)plumbaginaceae:34.827164):34.827156):34.827164):34.827164)caryophyllales:14.925919):7.222229,(((((Urobotrya_latisquama:38.518520)Urobotrya:38.518520,(Champereia_manillana:38.518520)Champereia:38.518520,(Lepionurus_sylvestris:38.518520)Lepionurus:38.518520,(Opilia_amentacea:38.518520)Opilia:38.518520,(Cansjera_rheedii:38.518520)Cansjera:38.518520)opiliaceae:38.518517,((Viscum_articulatum:38.518520,Viscum_multinerve:38.518520,Viscum_liquidambaricola:38.518520,Viscum_coloratum:38.518520,Viscum_loranthi:38.518520,Viscum_ovalifolium:38.518520,Viscum_album_var.meridianum:38.518520,Viscum_nudum:38.518520,Viscum_diospyrosicola:38.518520,Viscum_monoicum:38.518520,Viscum_fargesii:38.518520,Viscum_yunnanense:38.518520)Viscum:38.518520,(Korthalsella_japonica:38.518520)Korthalsella:38.518520,(Arceuthobium_pini:38.518520,Arceuthobium_tibetense:38.518520,Arceuthobium_chinense:38.518520,Arceuthobium_oxycedri:38.518520,Arceuthobium_pini_var.sichuanense:38.518520)Arceuthobium:38.518520,(Dendrotrophe_polyneura:38.518520,Dendrotrophe_buxifolia:38.518520,Dendrotrophe_frutescens:38.518520,Dendrotrophe_umbellata:38.518520,Dendrotrophe_heterantha:38.518520,Dendrotrophe_granulata:38.518520)Dendrotrophe:38.518520,(Osyris_wightiana:38.518520)Osyris:38.518520,(Scleropyrum_wallichianum_var.mekongense:38.518520,Scleropyrum_wallichianum:38.518520)Scleropyrum:38.518520)santalaceae:38.518517):38.518524,(((Helixanthera_scoriarum:38.518520,Helixanthera_guangxiensis:38.518520,Helixanthera_coccinea:38.518520,Helixanthera_parasitica:38.518520,Helixanthera_terrestris:38.518520,Helixanthera_pierrei:38.518520,Helixanthera_sampsoni:38.518520)Helixanthera:38.518520,(Loranthus_pseudo-odoratus:38.518520,Loranthus_kaoi:38.518520,Loranthus_delavayi:38.518520)Loranthus:38.518520,(Dendrophthoe_pentandra:38.518520)Dendrophthoe:38.518520,(Taxillus_thibetensis:38.518520,Taxillus_pseudochinensis:38.518520,Taxillus_chinensis:38.518520,Taxillus_sutchuenensis_var.duclouxii:38.518520,Taxillus_liquidambaricola:38.518520,Taxillus_balansae:38.518520,Taxillus_limprichtii_var.longiflorus:38.518520,Taxillus_delavayi:38.518520,Taxillus_sericus:38.518520,Taxillus_nigrans:38.518520,Taxillus_limprichtii:38.518520,Taxillus_umbelifer:38.518520,Taxillus_sutchuenensis:38.518520,Taxillus_caloreas:38.518520,Taxillus_theifer:38.518520,Taxillus_caloreas_var.fargesii:38.518520,Taxillus_kaempferi:38.518520,Taxillus_levinei:38.518520)Taxillus:38.518520,(Elytranthe_albida:38.518520)Elytranthe:38.518520,(Macrosolen_robinsonii:38.518520,Macrosolen_suberosus:38.518520,Macrosolen_cochinchinensis:38.518520,Macrosolen_bibracteolatus:38.518520)Macrosolen:38.518520,(Scurrula_pulverulenta:38.518520,Scurrula_buddleioides:38.518520,Scurrula_chingii_var.yunnanensis:38.518520,Scurrula_elata:38.518520,Scurrula_gongshanensis:38.518520,Scurrula_parasitica:38.518520,Scurrula_philippensis:38.518520,Scurrula_chingii:38.518520,Scurrula_parasitica_var.graciliflora:38.518520,Scurrula_notothixoides:38.518520,Scurrula_ferruginea:38.518520)Scurrula:38.518520)loranthaceae:38.518517):38.518524):38.518509,((Olax_austrosinensis:64.197533,Olax_wightiana:64.197533)Olax:64.197533,(Ximenia_americana:64.197533)Ximenia:64.197533,(Erythropalum_scandens:64.197533)Erythropalum:64.197533,(Schoepfia_fragrans:64.197533)Schoepfia:64.197533,(Malania_oleifera:64.197533)Malania:64.197533)olacaceae:64.197525)santalales:38.518524):7.222214):7.222229,((Dillenia_turbinata:81.851852,Dillenia_indica:81.851852)Dillenia:81.851852,(Tetracera_scandens:81.851852,Tetracera_asiatica:81.851852)Tetracera:81.851852)dilleniaceae:81.851852):7.222214)core_eudicots:7.222229,(((Sarcococca_vagans:65.000000,Sarcococca_saligna:65.000000,Sarcococca_ruscifolia:65.000000,Sarcococca_hookeriana:65.000000,Sarcococca_wallichii:65.000000,Sarcococca_longipetiolata:65.000000,Sarcococca_longifolia:65.000000)Sarcococca:65.000000,(Pachysandra_axillaris:65.000000,Pachysandra_terminalis:65.000000)Pachysandra:65.000000,(Buxus_mollicula_var.glabra:65.000000,Buxus_henryi:65.000000,Buxus_megistophylla:65.000000,Buxus_austro-yunnanensis:65.000000,Buxus_hainanensis:65.000000,Buxus_sinica:65.000000,Buxus_sinica_subsp.aemulans:65.000000,Buxus_latistyla:65.000000,Buxus_hebecarpa:65.000000,Buxus_pubiramea:65.000000,Buxus_rugulosa_var.prostrata:65.000000,Buxus_mollicula:65.000000,Buxus_harlandii:65.000000,Buxus_stenophylla:65.000000,Buxus_myrica_var.angustifolia:65.000000,Buxus_linearifolia:65.000000,Buxus_sinica_var.parvifolia:65.000000,Buxus_rugulosa_subsp.rupicola:65.000000,Buxus_myrica:65.000000,Buxus_ichangensis:65.000000,Buxus_sinica_var.vacciniifolia:65.000000,Buxus_sinica_var.intermedia:65.000000,Buxus_rugulosa:65.000000)Buxus:65.000000)buxaceae:65.000000)buxales:65.000000,(((Trochodendron_aralioides:65.000000)Trochodendron:65.000000)trochodendraceae:65.000000)trochodendrales:65.000000)trochodendrales_to_asterales:7.222229,((Meliosma_angustifolia:89.074074,Meliosma_glandulosa:89.074074,Meliosma_fordii:89.074074,Meliosma_fordii_var.sinii:89.074074,Meliosma_rhoifolia_var.barbulata:89.074074,Meliosma_pinnata:89.074074,Meliosma_yunnanensis:89.074074,Meliosma_rigida_var.pannosa:89.074074,Meliosma_squamulata:89.074074,Meliosma_callicarpaefolia:89.074074,Meliosma_rigida:89.074074,Meliosma_dumicola:89.074074,Meliosma_henryi:89.074074,Meliosma_laui:89.074074,Meliosma_arnottiana:89.074074,Meliosma_rhoifolia:89.074074,Meliosma_thorelii:89.074074,Meliosma_longipes:89.074074,Meliosma_bifida:89.074074,Meliosma_paupera:89.074074)Meliosma:89.074074,(Sabia_fasciculata:89.074074,Sabia_coriacea:89.074074,Sabia_swinhoei:89.074074,Sabia_limoniacea:89.074074,Sabia_parviflora:89.074074,Sabia_nervosa:89.074074,Sabia_paniculata:89.074074)Sabia:89.074074)sabiaceae:89.074081,((((Helicia_reticulata:100.208336,Helicia_tibetensis:100.208336,Helicia_cochinchinensis:100.208336,Helicia_vestita_var.longipes:100.208336,Helicia_pyrrhobotrya:100.208336,Helicia_yangchunensis:100.208336,Helicia_longipetiolata:100.208336,Helicia_grandis:100.208336,Helicia_obovatifolia:100.208336,Helicia_cauliflora:100.208336,Helicia_kwangtungensis:100.208336,Helicia_hainanensis:100.208336,Helicia_falcata:100.208336,Helicia_silvicola:100.208336,Helicia_tsaii:100.208336,Helicia_vestita:100.208336,Helicia_obovatifolia_var.mixta:100.208336,Helicia_shweliensis:100.208336,Helicia_formosana:100.208336,Helicia_nilagirica:100.208336)Helicia:100.208336,((((((((Heliciopsis_henryi:22.268518,Heliciopsis_lobata:22.268518,Heliciopsis_terminalis:22.268518)heliciopsis:22.268518):22.268520):22.268517):22.268524):22.268517):22.268524):22.268509):22.268524)proteaceae:22.268524):22.268509)proteales:22.268524)sabiales_to_asterales:7.222229,((((((Pycnarrhena_poilanei:45.740742)Pycnarrhena:45.740742,(Stephania_sasakii:45.740742)Stephania:45.740742,(Diploclisia_glaucescens:45.740742,Diploclisia_affinis:45.740742)Diploclisia:45.740742,(Cissampelos_pareira_var.hirsuta:45.740742)Cissampelos:45.740742,(Pericampylus_glaucus:45.740742)Pericampylus:45.740742,(Fibraurea_recisa:45.740742)Fibraurea:45.740742,(Cyclea_hypoglauca:45.740742,Cyclea_insularis:45.740742,Cyclea_polypetala:45.740742,Cyclea_wattii:45.740742,Cyclea_debiliflora:45.740742,Cyclea_meeboldii:45.740742)Cyclea:45.740742,(Tinomiscium_petiolare:45.740742)Tinomiscium:45.740742,(Sinomenium_acutum:45.740742)Sinomenium:45.740742,(Albertisia_laurifolia:45.740742)Albertisia:45.740742,(Pachygone_valida:45.740742)Pachygone:45.740742,(Aspidocarya_uvifera:45.740742)Aspidocarya:45.740742,(Eleutharrhena_macrocarpa:45.740742)Eleutharrhena:45.740742,(Cocculus_orbiculatus_var.mollis:45.740742,Cocculus_laurifolius:45.740742)Cocculus:45.740742,(Hypserpa_nitida:45.740742)Hypserpa:45.740742)menispermaceae:45.740746,(((Berberis_grodtmannia:34.305557,Berberis_veitchii:34.305557,Berberis_guizhouensis:34.305557,Berberis_tsangpoensis:34.305557,Berberis_vernalis:34.305557,Berberis_sargentiana:34.305557,Berberis_polyantha:34.305557,Berberis_dumicola:34.305557,Berberis_dasyclada:34.305557,Berberis_griffithiana:34.305557,Berberis_taliensis:34.305557,Berberis_ferdinandi-coburgii:34.305557,Berberis_candidula:34.305557,Berberis_taronensis:34.305557,Berberis_centiflora:34.305557,Berberis_delavayi:34.305557,Berberis_bicolor:34.305557,Berberis_xanthoclada:34.305557,Berberis_pruinosa:34.305557,Berberis_holocraspedon:34.305557,Berberis_fengqingensis:34.305557,Berberis_alpicola:34.305557,Berberis_cavaleriei:34.305557,Berberis_bergmanniae:34.305557,Berberis_julianae:34.305557,Berberis_atrocarpa:34.305557,Berberis_gagnepainii_var.gagnepainii:34.305557,Berberis_hookeri:34.305557,Berberis_chingii:34.305557,Berberis_chrysosphaera:34.305557,Berberis_griffithiana_var.pallida:34.305557,Berberis_paraspecta:34.305557,Berberis_soulieana:34.305557,Berberis_fallax:34.305557,Berberis_acuminata:34.305557,Berberis_jiangxiensis:34.305557,Berberis_wilsonae:34.305557,Berberis_sublevis:34.305557,Berberis_jiulongensis:34.305557,Berberis_replicata:34.305557,Berberis_coxii:34.305557,Berberis_lijiangensis:34.305557,Berberis_lubrica:34.305557,Berberis_obovatifolia:34.305557,Berberis_nemorosa:34.305557,Berberis_heteropsis:34.305557,Berberis_malipoensis:34.305557,Berberis_triacanthophora:34.305557,Berberis_calliantha:34.305557,Berberis_aristato-serrulata:34.305557,Berberis_davidii:34.305557,Berberis_jinfoshanensis:34.305557,Berberis_fallaciosa:34.305557,Berberis_hayatana:34.305557,Berberis_impedita:34.305557,Berberis_nantoensis:34.305557,Berberis_levis:34.305557,Berberis_pinshanensis:34.305557,Berberis_pulangensis:34.305557,Berberis_insignis_subsp.incrassata:34.305557,Berberis_sabulicola:34.305557,Berberis_potaninii:34.305557,Berberis_photiniaefolia:34.305557,Berberis_petrogena:34.305557,Berberis_kawakamii:34.305557,Berberis_lempergiana:34.305557,Berberis_fujianensis:34.305557,Berberis_insolita:34.305557,Berberis_wangii:34.305557,Berberis_sikkimensis:34.305557,Berberis_asiatica:34.305557,Berberis_phanera:34.305557,Berberis_simulans:34.305557,Berberis_silvicola:34.305557,Berberis_hsuyunensis:34.305557,Berberis_sanguinea:34.305557,Berberis_subacuminata:34.305557,Berberis_yingjingensis:34.305557,Berberis_verruculosa:34.305557,Berberis_asmyana:34.305557,Berberis_deinacantha:34.305557,Berberis_ziyunensis:34.305557)Berberis:34.305557,(Nandina_domestica:34.305557)Nandina:34.305557,(Mahonia_oiwakensis:34.305557,Mahonia_eurybracteata_subsp.ganpinensis:34.305557,Mahonia_fordii:34.305557,Mahonia_calamicaulis_subsp.kingdon-wardiana:34.305557,Mahonia_setosa:34.305557,Mahonia_hancockiana:34.305557,Mahonia_taronensis:34.305557,Mahonia_breviracema:34.305557,Mahonia_polydonta:34.305557,Mahonia_decipiens:34.305557,Mahonia_bracteolata:34.305557,Mahonia_paucijuga:34.305557,Mahonia_subimbricata:34.305557,Mahonia_eurybracteata:34.305557,Mahonia_bealei:34.305557,Mahonia_nitens:34.305557,Mahonia_monyulensis:34.305557,Mahonia_conferta:34.305557,Mahonia_napaulensis:34.305557,Mahonia_shenii:34.305557,Mahonia_fortunei:34.305557,Mahonia_japonica:34.305557,Mahonia_retinervis:34.305557,Mahonia_gracilipes:34.305557,Mahonia_leptodonta:34.305557,Mahonia_bodinieri:34.305557,Mahonia_longibracteata:34.305557,Mahonia_sheridaniana:34.305557,Mahonia_duclouxiana:34.305557)Mahonia:34.305557)berberidaceae:34.305557,((Naravelia_zeylanica:34.305557)Naravelia:34.305557)ranunculaceae:34.305557):34.305557):34.305557,(((Stauntonia_conspicua:42.881947,Stauntonia_duclouxii:42.881947,Stauntonia_yaoshanensis:42.881947,Stauntonia_chinensis:42.881947,Stauntonia_maculata:42.881947,Stauntonia_decora:42.881947,Stauntonia_obovata:42.881947,Stauntonia_obcordatilimba:42.881947,Stauntonia_leucantha:42.881947,Stauntonia_glauca:42.881947,Stauntonia_brachyanthera:42.881947,Stauntonia_pseudomaculata:42.881947,Stauntonia_elliptica:42.881947,Stauntonia_trinervia:42.881947,Stauntonia_brunoniana:42.881947,Stauntonia_oligophylla:42.881947,Stauntonia_obovatifoliola:42.881947,Stauntonia_obovatifoliola_subsp.urophylla:42.881947,Stauntonia_cavalerieana:42.881947)Stauntonia:42.881947,(Holboellia_latifolia:42.881947,Holboellia_pterocaulis:42.881947,Holboellia_grandiflora:42.881947,Holboellia_chapaensis:42.881947,Holboellia_fargesii:42.881947,Holboellia_parviflora:42.881947,Holboellia_coriacea:42.881947)Holboellia:42.881947,(Akebia_longeracemosa:42.881947)Akebia:42.881947)lardizabalaceae:42.881950):42.881943):34.305557):34.305557)ranunculales:34.305557)eudicots:7.222198)ceratophyllales_and_eudicots:7.222229)poales_to_asterales:7.222229,(((((((((Orophea_hainanensis:29.611111,Orophea_multiflora:29.611111,Orophea_polycarpa:29.611111,Orophea_hirsuta:29.611111,Orophea_yunnanensis:29.611111)Orophea:29.611111,(Uvaria_calamistrata:29.611111,Uvaria_grandiflora:29.611111,Uvaria_tonkinensis:29.611111,Uvaria_boniana:29.611111,Uvaria_kweichowensis:29.611111,Uvaria_kurzii:29.611111,Uvaria_rufa:29.611111,Uvaria_microcarpa:29.611111)Uvaria:29.611111,(Anaxagorea_luzonensis:29.611111)Anaxagorea:29.611111,(Mezzettiopsis_creaghii:29.611111)Mezzettiopsis:29.611111,(Fissistigma_retusum:29.611111,Fissistigma_glaucescens:29.611111,Fissistigma_latifolium:29.611111,Fissistigma_tungfangense:29.611111,Fissistigma_cavaleriei:29.611111,Fissistigma_bracteolatum:29.611111,Fissistigma_polyanthum:29.611111,Fissistigma_balansae:29.611111,Fissistigma_oldhamii:29.611111,Fissistigma_kwangsiense:29.611111,Fissistigma_wallichii:29.611111,Fissistigma_poilanei:29.611111,Fissistigma_acuminatissimum:29.611111,Fissistigma_cupreonitens:29.611111,Fissistigma_chloroneurum:29.611111,Fissistigma_tonkinense:29.611111,Fissistigma_xylopetalum:29.611111,Fissistigma_shangtzeense:29.611111,Fissistigma_tientangense:29.611111,Fissistigma_uonicum:29.611111,Fissistigma_polyanthoides:29.611111)Fissistigma:29.611111,(Cyathostemma_yunnanense:29.611111)Cyathostemma:29.611111,(Popowia_pisocarpa:29.611111)Popowia:29.611111,(Pseuduvaria_indochinensis:29.611111)Pseuduvaria:29.611111,(Alphonsea_squamosa:29.611111,Alphonsea_tsangyuanensis:29.611111,Alphonsea_hainanensis:29.611111,Alphonsea_boniana:29.611111,Alphonsea_mollis:29.611111,Alphonsea_monogyna:29.611111)Alphonsea:29.611111,(Meiogyne_kwangtungensis:29.611111)Meiogyne:29.611111,(Goniothalamus_gabriacianus:29.611111,Goniothalamus_griffithii:29.611111,Goniothalamus_chinensis:29.611111,Goniothalamus_howii:29.611111,Goniothalamus_leiocarpus:29.611111,Goniothalamus_cheliensis:29.611111,Goniothalamus_amuyon:29.611111,Goniothalamus_donnaiensis:29.611111,Goniothalamus_yunnanensis:29.611111,Goniothalamus_gardneri:29.611111)Goniothalamus:29.611111,(Desmos_macrocalyx:29.611111,Desmos_grandifolius:29.611111,Desmos_robinsonii:29.611111,Desmos_sootepensis:29.611111,Desmos_rostratus:29.611111,Desmos_chinensis:29.611111,Desmos_saccopetaloides:29.611111,Desmos_dumosus:29.611111,Desmos_yunnanensis:29.611111)Desmos:29.611111,(Xylopia_vielana:29.611111)Xylopia:29.611111,(Artabotrys_punctulatus:29.611111,Artabotrys_multiflorus:29.611111,Artabotrys_hainanensis:29.611111,Artabotrys_rhynchocarpus:29.611111,Artabotrys_pilosus:29.611111,Artabotrys_hongkongensis:29.611111,Artabotrys_fragrans:29.611111,Artabotrys_hexapetalus:29.611111)Artabotrys:29.611111,(Richella_hainanensis:29.611111)Richella:29.611111,(Miliusa_prolifica:29.611111,Miliusa_cuneata:29.611111,Miliusa_chunii:29.611111,Miliusa_tenuistipitata:29.611111,Miliusa_sinensis:29.611111)Miliusa:29.611111,(Mitrephora_maingayi:29.611111,Mitrephora_thorelii:29.611111,Mitrephora_wangii:29.611111)Mitrephora:29.611111,(Polyalthia_suberosa:29.611111,Polyalthia_pingpienensis:29.611111,Polyalthia_laui:29.611111,Polyalthia_lancilimba:29.611111,Polyalthia_nemoralis:29.611111,Polyalthia_viridis:29.611111,Polyalthia_litseifolia:29.611111,Polyalthia_obliqua:29.611111,Polyalthia_cerasoides:29.611111,Polyalthia_simiarum:29.611111,Polyalthia_rumphii:29.611111,Polyalthia_florulenta:29.611111,Polyalthia_plagioneura:29.611111,Polyalthia_verrucipes:29.611111,Polyalthia_petelotii:29.611111)Polyalthia:29.611111,(Oncodostigma_hainanense:29.611111)Oncodostigma:29.611111)annonaceae:29.611115):29.611107):29.611115,((Michelia_mediocris:49.351852,Michelia_szechuanica:49.351852,Michelia_floribunda:49.351852,Michelia_wilsonii:49.351852,Michelia_fujianensis:49.351852,Michelia_guangdongensis:49.351852,Michelia_guangxiensis:49.351852,Michelia_baillonii:49.351852,Michelia_xanthantha:49.351852,Michelia_champaca:49.351852,Michelia_martinii:49.351852,Michelia_foveolata:49.351852,Michelia_balansae:49.351852,Michelia_cavaleriei_var.platypetala:49.351852,Michelia_chapensis:49.351852,Michelia_champaca_var.pubinervia:49.351852,Michelia_doltsopa:49.351852,Michelia_cavaleriei:49.351852,Michelia_masticata:49.351852,Michelia_sphaerantha:49.351852,Michelia_velutina:49.351852,Michelia_maudiae:49.351852,Michelia_shiluensis:49.351852,Michelia_iteophylla:49.351852,Michelia_flaviflora:49.351852,Michelia_compressa:49.351852,Michelia_kisopa:49.351852,Michelia_coriacea:49.351852,Michelia_angustioblonga:49.351852,Michelia_gioi:49.351852,Michelia_elegans:49.351852,Michelia_skinneriana:49.351852,Michelia_yunnanensis:49.351852,Michelia_leveilleana:49.351852,Michelia_lacei:49.351852,Michelia_crassipes:49.351852,Michelia_fulva:49.351852,Michelia_macclurei:49.351852)Michelia:49.351852,(Lirianthe_odoratissima:49.351852,Lirianthe_coco:49.351852,Lirianthe_henryi:49.351852,Lirianthe_fujianensis:49.351852,Lirianthe_albosericea:49.351852,Lirianthe_delavayi:49.351852,Lirianthe_fistulosa:49.351852,Lirianthe_championii:49.351852)Lirianthe:49.351852,(Tsoongiodendron_odorum:49.351852)Tsoongiodendron:49.351852,(Manglietia_patungensis:49.351852,Manglietia_glaucifolia:49.351852,Manglietia_duclouxii:49.351852,Manglietia_crassipes:49.351852,Manglietia_grandis:49.351852,Manglietia_dandyi:49.351852,Manglietia_obovalifolia:49.351852,Manglietia_forrestii:49.351852,Manglietia_conifera:49.351852,Manglietia_fordiana_var.hainanensis:49.351852,Manglietia_hongheensis:49.351852,Manglietia_pachyphylla:49.351852,Manglietia_lucida:49.351852,Manglietia_ovoidea:49.351852,Manglietia_rufibarbata:49.351852,Manglietia_ventii:49.351852,Manglietia_kwangtungensis:49.351852,Manglietia_fordiana:49.351852,Manglietia_chevalieri:49.351852,Manglietia_sinoconifera:49.351852,Manglietia_calcarea:49.351852,Manglietia_szechuanica:49.351852,Manglietia_garrettii:49.351852,Manglietia_oblonga:49.351852,Manglietia_caveana:49.351852,Manglietia_aromatica:49.351852,Manglietia_zhengyiana:49.351852,Manglietia_hookeri:49.351852)Manglietia:49.351852,(Manglietiastrum_sinicum:49.351852)Manglietiastrum:49.351852,(Parakmeria_omeiensis:49.351852,Parakmeria_nitida:49.351852,Parakmeria_kachirachirai:49.351852,Parakmeria_lotungensis:49.351852,Parakmeria_yunnanensis:49.351852)Parakmeria:49.351852,(Talauma_hodgsoni:49.351852)Talauma:49.351852)magnoliaceae:49.351852):29.611115,((Knema_linifolia:59.222225,Knema_tenuinervia:59.222225,Knema_elegans:59.222225,Knema_tonkinensis:59.222225,Knema_lenta:59.222225,Knema_globularia:59.222225)Knema:59.222225,(Myristica_yunnanensis:59.222225)Myristica:59.222225,(Horsfieldia_kingii:59.222225,Horsfieldia_glabra:59.222225,Horsfieldia_prainii:59.222225)Horsfieldia:59.222225)myristicaceae:59.222221)magnoliales:29.611115,((((((Actinodaphne_glaucina:29.611113,Actinodaphne_paotingensis:29.611113,Actinodaphne_obovata:29.611113,Actinodaphne_omeiensis:29.611113,Actinodaphne_koshepangii:29.611113,Actinodaphne_cupularis:29.611113,Actinodaphne_lecomtei:29.611113,Actinodaphne_tsaii:29.611113,Actinodaphne_trichocarpa:29.611113,Actinodaphne_pilosa:29.611113,Actinodaphne_forrestii:29.611113,Actinodaphne_menghaiensis:29.611113,Actinodaphne_acuminata:29.611113,Actinodaphne_kweichowensis:29.611113,Actinodaphne_henryi:29.611113,Actinodaphne_mushanensis:29.611113,Actinodaphne_obscurinervia:29.611113)Actinodaphne:29.611113,(Dodecadenia_grandiflora_var.griffithii:29.611113)Dodecadenia:29.611113,(Cinnamomum_ilicioides:29.611113,Cinnamomum_tamala:29.611113,Cinnamomum_micranthum:29.611113,Cinnamomum_wilsonii:29.611113,Cinnamomum_validinerve:29.611113,Cinnamomum_iners:29.611113,Cinnamomum_pittosporoides:29.611113,Cinnamomum_austro-yunnanense:29.611113,Cinnamomum_brachythyrsum:29.611113,Cinnamomum_bejolghota:29.611113,Cinnamomum_philippinense:29.611113,Cinnamomum_kwangtungense:29.611113,Cinnamomum_bodinieri:29.611113,Cinnamomum_austrosinense:29.611113,Cinnamomum_parthenoxylon:29.611113,Cinnamomum_tonkinense:29.611113,Cinnamomum_chartophyllum:29.611113,Cinnamomum_contractum:29.611113,Cinnamomum_platyphyllum:29.611113,Cinnamomum_tsangii:29.611113,Cinnamomum_kotoense:29.611113,Cinnamomum_rigidissimum:29.611113,Cinnamomum_appelianum:29.611113,Cinnamomum_mollifolium:29.611113,Cinnamomum_migao:29.611113,Cinnamomum_tsoi:29.611113,Cinnamomum_pingbienense:29.611113,Cinnamomum_rufotomentosum:29.611113,Cinnamomum_cassia:29.611113,Cinnamomum_liangii:29.611113,Cinnamomum_pauciflorum:29.611113,Cinnamomum_japonicum:29.611113,Cinnamomum_osmophloeum:29.611113,Cinnamomum_reticulatum:29.611113,Cinnamomum_foveolatum:29.611113,Cinnamomum_tenuipilum:29.611113,Cinnamomum_heyneanum:29.611113,Cinnamomum_subavenium:29.611113,Cinnamomum_saxatile:29.611113,Cinnamomum_jensenianum:29.611113,Cinnamomum_burmannii:29.611113,Cinnamomum_septentrionale:29.611113,Cinnamomum_mairei:29.611113,Cinnamomum_longepaniculatum:29.611113,Cinnamomum_glanduliferum:29.611113,Cinnamomum_camphora:29.611113,Cinnamomum_longipetiolatum:29.611113,Cinnamomum_javanicum:29.611113)Cinnamomum:29.611113,(Phoebe_glaucifolia:29.611113,Phoebe_neurantha:29.611113,Phoebe_hainanensis:29.611113,Phoebe_crassipedicella:29.611113,Phoebe_megacalyx:29.611113,Phoebe_macrocarpa:29.611113,Phoebe_brachythyrsa:29.611113,Phoebe_neurantha_var.brevifolia:29.611113,Phoebe_sheareri_var.omeiensis:29.611113,Phoebe_glaucophylla:29.611113,Phoebe_neuranthoides:29.611113,Phoebe_kwangsiensis:29.611113,Phoebe_nigrifolia:29.611113,Phoebe_rufescens:29.611113,Phoebe_hungmaoensis:29.611113,Phoebe_yunnanensis:29.611113,Phoebe_lichuanensis:29.611113,Phoebe_bournei:29.611113,Phoebe_motuonan:29.611113,Phoebe_zhennan:29.611113,Phoebe_lanceolata:29.611113,Phoebe_puwenensis:29.611113,Phoebe_chinensis:29.611113,Phoebe_calcarea:29.611113,Phoebe_formosana:29.611113,Phoebe_tavoyana:29.611113,Phoebe_hui:29.611113,Phoebe_hunanensis:29.611113,Phoebe_minutiflora:29.611113,Phoebe_microphylla:29.611113,Phoebe_neurantha_var.cavaleriei:29.611113,Phoebe_yaiensis:29.611113,Phoebe_legendrei:29.611113,Phoebe_forrestii:29.611113,Phoebe_angustifolia:29.611113,Phoebe_chekiangensis:29.611113,Phoebe_faberi:29.611113,Phoebe_sheareri:29.611113)Phoebe:29.611113,(Litsea_kobuskiana:29.611113,Litsea_glutinosa_var.brideliifolia:29.611113,Litsea_albescens:29.611113,Litsea_coreana_var.sinensis:29.611113,Litsea_depressa:29.611113,Litsea_liboshengii:29.611113,Litsea_vang_var.lobata:29.611113,Litsea_cangyuanensis:29.611113,Litsea_mishmiensis:29.611113,Litsea_rotundifolia_var.oblongifolia:29.611113,Litsea_glutinosa:29.611113,Litsea_coreana:29.611113,Litsea_baviensis:29.611113,Litsea_lancilimba:29.611113,Litsea_monantha:29.611113,Litsea_martabanica:29.611113,Litsea_foveolata:29.611113,Litsea_viridis:29.611113,Litsea_gongshanensis:29.611113,Litsea_kwangtungensis:29.611113,Litsea_subcoriacea:29.611113,Litsea_litseaefolia:29.611113,Litsea_pittosporifolia:29.611113,Litsea_salicifolia:29.611113,Litsea_honghoensis:29.611113,Litsea_kwangsiensis:29.611113,Litsea_pedunculata:29.611113,Litsea_hupehana:29.611113,Litsea_hunanensis:29.611113,Litsea_greenmaniana:29.611113,Litsea_variabilis:29.611113,Litsea_elongata:29.611113,Litsea_hypophaea:29.611113,Litsea_balansae:29.611113,Litsea_monopetala:29.611113,Litsea_acutivena:29.611113,Litsea_lancifolia:29.611113,Litsea_chinpingensis:29.611113,Litsea_elongata_var.subverticillata:29.611113,Litsea_akoensis_var.sasakii:29.611113,Litsea_garciae:29.611113,Litsea_coelestis:29.611113,Litsea_rotundifolia_var.ovatifolia:29.611113,Litsea_verticillata:29.611113,Litsea_coreana_var.lanuginosa:29.611113,Litsea_pedunculata_var.pubescens:29.611113,Litsea_variabilis_var.oblonga:29.611113,Litsea_akoensis:29.611113,Litsea_verticillifolia:29.611113,Litsea_beilschmiediifolia:29.611113,Litsea_wilsonii:29.611113,Litsea_machiloides:29.611113,Litsea_umbellata:29.611113,Litsea_biflora:29.611113,Litsea_oligophlebia:29.611113,Litsea_elongata_var.faberi:29.611113,Litsea_chengshuzhii:29.611113,Litsea_suberosa:29.611113,Litsea_szemois:29.611113,Litsea_lancifolia_var.ellipsoidea:29.611113,Litsea_dilleniifolia:29.611113,Litsea_tibetana:29.611113,Litsea_panamonja:29.611113,Litsea_yaoshanensis:29.611113,Litsea_lancifolia_var.pedicellata:29.611113,Litsea_semecarpifolia:29.611113,Litsea_morrisonensis:29.611113,Litsea_sinoglobosa:29.611113,Litsea_rotundifolia:29.611113,Litsea_liyuyingi:29.611113,Litsea_yunnanensis:29.611113,Litsea_longistaminata:29.611113,Litsea_pseudoelongata:29.611113)Litsea:29.611113,(Beilschmiedia_fasciata:29.611113,Beilschmiedia_baotingensis:29.611113,Beilschmiedia_roxburghiana:29.611113,Beilschmiedia_robusta:29.611113,Beilschmiedia_yunnanensis:29.611113,Beilschmiedia_punctilimba:29.611113,Beilschmiedia_glauca_var.glaucoides:29.611113,Beilschmiedia_tungfangensis:29.611113,Beilschmiedia_brevipaniculata:29.611113,Beilschmiedia_brevifolia:29.611113,Beilschmiedia_glauca:29.611113,Beilschmiedia_fordii:29.611113,Beilschmiedia_kweichowensis:29.611113,Beilschmiedia_wangii:29.611113,Beilschmiedia_henghsienensis:29.611113,Beilschmiedia_rufohirtella:29.611113,Beilschmiedia_laevis:29.611113,Beilschmiedia_percoriacea:29.611113,Beilschmiedia_furfuracea:29.611113,Beilschmiedia_linocieroides:29.611113,Beilschmiedia_muricata:29.611113,Beilschmiedia_ovoidea:29.611113,Beilschmiedia_delicata:29.611113,Beilschmiedia_brachythyrsa:29.611113,Beilschmiedia_ningmingensis:29.611113,Beilschmiedia_intermedia:29.611113,Beilschmiedia_macropoda:29.611113,Beilschmiedia_appendiculata:29.611113,Beilschmiedia_shangsiensis:29.611113,Beilschmiedia_pauciflora:29.611113,Beilschmiedia_erythrophloia:29.611113,Beilschmiedia_tsangii:29.611113,Beilschmiedia_sichourensis:29.611113,Beilschmiedia_obconica:29.611113,Beilschmiedia_obscurinervia:29.611113,Beilschmiedia_percoriacea_var.ciliata:29.611113,Beilschmiedia_longipetiolata:29.611113,Beilschmiedia_pergamentacea:29.611113,Beilschmiedia_cylindrica:29.611113,Beilschmiedia_purpurascens:29.611113)Beilschmiedia:29.611113,(Cryptocarya_maclurei:29.611113,Cryptocarya_maculata:29.611113,Cryptocarya_densiflora:29.611113,Cryptocarya_brachythyrsa:29.611113,Cryptocarya_impressinervia:29.611113,Cryptocarya_elliptifolia:29.611113,Cryptocarya_kwangtungensis:29.611113,Cryptocarya_hainanensis:29.611113,Cryptocarya_tsangii:29.611113,Cryptocarya_chinensis:29.611113,Cryptocarya_concinna:29.611113,Cryptocarya_leiana:29.611113,Cryptocarya_acutifolia:29.611113,Cryptocarya_lyoniifolia:29.611113,Cryptocarya_depauperata:29.611113,Cryptocarya_amygdalina:29.611113,Cryptocarya_yaanica:29.611113,Cryptocarya_calcicola:29.611113,Cryptocarya_chingii:29.611113,Cryptocarya_yunnanensis:29.611113,Cryptocarya_metcalfiana:29.611113)Cryptocarya:29.611113,(Machilus_cavaleriei:29.611113,Machilus_melanophylla:29.611113,Machilus_leptophylla:29.611113,Machilus_platycarpa:29.611113,Machilus_ovatiloba:29.611113,Machilus_submultinervia:29.611113,Machilus_chayuensis:29.611113,Machilus_chuanchienensis:29.611113,Machilus_robusta:29.611113,Machilus_fasciculata:29.611113,Machilus_grandibracteata:29.611113,Machilus_obovatifolia:29.611113,Machilus_daozhenensis:29.611113,Machilus_yunnanensis:29.611113,Machilus_dinganensis:29.611113,Machilus_longipes:29.611113,Machilus_velutinoides:29.611113,Machilus_breviflora:29.611113,Machilus_multinervia:29.611113,Machilus_microcarpa_var.omeiensis:29.611113,Machilus_glaucifolia:29.611113,Machilus_phoenicis:29.611113,Machilus_gongshanensis:29.611113,Machilus_dumicola:29.611113,Machilus_glabrophylla:29.611113,Machilus_kwangtungensis:29.611113,Machilus_rufipes:29.611113,Machilus_thunbergii:29.611113,Machilus_chinensis:29.611113,Machilus_salicoides:29.611113,Machilus_ichangensis_var.leiophylla:29.611113,Machilus_chrysotricha:29.611113,Machilus_grijsii:29.611113,Machilus_gamblei:29.611113,Machilus_calcicola:29.611113,Machilus_decursinervis:29.611113,Machilus_monticola:29.611113,Machilus_oreophila:29.611113,Machilus_cicatricosa:29.611113,Machilus_lohuiensis:29.611113,Machilus_pomifera:29.611113,Machilus_lichuanensis:29.611113,Machilus_salicina:29.611113,Machilus_oculodracontis:29.611113,Machilus_viridis:29.611113,Machilus_mangdangshanensis:29.611113,Machilus_miaoshanensis:29.611113,Machilus_minkweiensis:29.611113,Machilus_fukienensis:29.611113,Machilus_litseifolia:29.611113,Machilus_nakao:29.611113,Machilus_nanchuanensis:29.611113,Machilus_pauhoi:29.611113,Machilus_bonii:29.611113,Machilus_chienkweiensis:29.611113,Machilus_austroguizhouensis:29.611113,Machilus_zuihoensis_var.mushaensis:29.611113,Machilus_foonchewii:29.611113,Machilus_velutina:29.611113,Machilus_glauecscens:29.611113,Machilus_gracillima:29.611113,Machilus_shweliensis:29.611113,Machilus_nanmu:29.611113,Machilus_parabreviflora:29.611113,Machilus_shiwandashanica:29.611113,Machilus_sichuanensis:29.611113,Machilus_pyramidalis:29.611113,Machilus_kurzii:29.611113,Machilus_reticulata:29.611113,Machilus_wenshanensis:29.611113,Machilus_sichourensis:29.611113,Machilus_tenuipila:29.611113,Machilus_rehderi:29.611113,Machilus_zuihoensis:29.611113,Machilus_microcarpa:29.611113,Machilus_wangchiana:29.611113,Machilus_minutiloba:29.611113,Machilus_ichangensis:29.611113,Machilus_obscurinervia:29.611113,Machilus_lenticellata:29.611113,Machilus_verruculosa:29.611113,Machilus_duthiei:29.611113,Machilus_chekiangensis:29.611113)Machilus:29.611113,(Neocinnamomum_mekongense:29.611113,Neocinnamomum_fargesii:29.611113,Neocinnamomum_caudatum:29.611113,Neocinnamomum_lecomtei:29.611113,Neocinnamomum_delavayi:29.611113)Neocinnamomum:29.611113,(Neolitsea_impressa:29.611113,Neolitsea_howii:29.611113,Neolitsea_variabillima:29.611113,Neolitsea_undulatifolia:29.611113,Neolitsea_confertifolia:29.611113,Neolitsea_daibuensis:29.611113,Neolitsea_levinei:29.611113,Neolitsea_brevipes:29.611113,Neolitsea_obtusifolia:29.611113,Neolitsea_polycarpa:29.611113,Neolitsea_aurata_var.glauca:29.611113,Neolitsea_kwangsiensis:29.611113,Neolitsea_hainanensis:29.611113,Neolitsea_pallens:29.611113,Neolitsea_acuminatissima:29.611113,Neolitsea_chrysotricha:29.611113,Neolitsea_villosa:29.611113,Neolitsea_lunglingensis:29.611113,Neolitsea_ovatifolia:29.611113,Neolitsea_velutina:29.611113,Neolitsea_pulchella:29.611113,Neolitsea_menglaensis:29.611113,Neolitsea_hiiranensis:29.611113,Neolitsea_zeylanica:29.611113,Neolitsea_pingbienensis:29.611113,Neolitsea_tomentosa:29.611113,Neolitsea_sutchuanensis:29.611113,Neolitsea_aciculata:29.611113,Neolitsea_homilantha:29.611113,Neolitsea_wushanica:29.611113,Neolitsea_konishii:29.611113,Neolitsea_buisanensis:29.611113,Neolitsea_alongensis:29.611113,Neolitsea_phanerophlebia:29.611113,Neolitsea_cambodiana_var.glabra:29.611113,Neolitsea_ellipsoidea:29.611113,Neolitsea_hsiangkweiensis:29.611113,Neolitsea_parvigemma:29.611113,Neolitsea_aurata:29.611113,Neolitsea_shingningensis:29.611113,Neolitsea_cambodiana:29.611113,Neolitsea_chuii:29.611113,Neolitsea_pinninervis:29.611113,Neolitsea_aurata_var.paraciculata:29.611113,Neolitsea_longipedicellata:29.611113,Neolitsea_oblongifolia:29.611113,Neolitsea_aurata_var.chekiangensis:29.611113,Neolitsea_aurata_var.undulatula:29.611113,Neolitsea_sericea:29.611113,Neolitsea_purpurascens:29.611113)Neolitsea:29.611113,(Nothaphoebe_cavaleriei:29.611113,Nothaphoebe_konishii:29.611113)Nothaphoebe:29.611113,(Sinosassafras_flavinervium:29.611113)Sinosassafras:29.611113,(Alseodaphne_hokouensis:29.611113,Alseodaphne_huanglianshanensis:29.611113,Alseodaphne_marlipoensis:29.611113,Alseodaphne_andersonii:29.611113,Alseodaphne_sichourensis:29.611113,Alseodaphne_gracilis:29.611113,Alseodaphne_hainanensis:29.611113,Alseodaphne_yunnanensis:29.611113,Alseodaphne_petiolaris:29.611113,Alseodaphne_rugosa:29.611113)Alseodaphne:29.611113,(Caryodaphnopsis_laotica:29.611113,Caryodaphnopsis_tonkinensis:29.611113,Caryodaphnopsis_henryi:29.611113)Caryodaphnopsis:29.611113,(Parasassafras_confertiflorum:29.611113)Parasassafras:29.611113,(Syndiclis_anlungensis:29.611113,Syndiclis_fooningensis:29.611113,Syndiclis_kwangsiensis:29.611113,Syndiclis_lotungensis:29.611113,Syndiclis_furfuracea:29.611113,Syndiclis_marlipoensis:29.611113,Syndiclis_pingbienensis:29.611113,Syndiclis_sichourensis:29.611113,Syndiclis_chinensis:29.611113)Syndiclis:29.611113,(Lindera_pulcherrima_var.hemsleyana:29.611113,Lindera_metcalfiana:29.611113,Lindera_chunii:29.611113,Lindera_prattii:29.611113,Lindera_foveolata:29.611113,Lindera_doniana:29.611113,Lindera_kwangtungensis:29.611113,Lindera_guangxiensis:29.611113,Lindera_robusta:29.611113,Lindera_megaphylla:29.611113,Lindera_tonkinensis:29.611113,Lindera_supracostata:29.611113,Lindera_lungshengensis:29.611113,Lindera_limprichtii:29.611113,Lindera_villipes:29.611113,Lindera_menghaiensis:29.611113,Lindera_nacusua_var.monglunensis:29.611113,Lindera_floribunda:29.611113,Lindera_nacusua:29.611113,Lindera_thomsonii:29.611113,Lindera_longipedunculata:29.611113,Lindera_setchuenensis:29.611113,Lindera_akoensis:29.611113,Lindera_tienchuanensis:29.611113,Lindera_latifolia:29.611113,Lindera_metcalfiana_var.dictyophylla:29.611113,Lindera_aggregata:29.611113,Lindera_tonkinensis_var.subsessilis:29.611113,Lindera_pulcherrima:29.611113,Lindera_motuoensis:29.611113,Lindera_gracilipes:29.611113,Lindera_pulcherrima_var.attenuata:29.611113,Lindera_caudata:29.611113,Lindera_communis:29.611113,Lindera_fragrans:29.611113,Lindera_aggregata_var.playfairii:29.611113,Lindera_thomsonii_var.vernayana:29.611113)Lindera:29.611113,(Endiandra_coriacea:29.611113,Endiandra_hainanensis:29.611113,Endiandra_dolichocarpa:29.611113)Endiandra:29.611113,(Dehaasia_kwangtungensis:29.611113,Dehaasia_hainanensis:29.611113,Dehaasia_incrassata:29.611113)Dehaasia:29.611113)lauraceae:29.611111,((Hernandia_sonora:29.611113)Hernandia:29.611113,(Illigera_grandiflora:29.611113,Illigera_trifoliata_subsp.cucullata:29.611113,Illigera_brevistaminata:29.611113,Illigera_cordata_var.mollissima:29.611113,Illigera_rhodantha:29.611113,Illigera_celebica:29.611113,Illigera_luzonensis:29.611113,Illigera_pseudoparviflora:29.611113,Illigera_glabra:29.611113,Illigera_nervosa:29.611113,Illigera_aromatica:29.611113,Illigera_parviflora:29.611113,Illigera_cordata:29.611113,Illigera_rhodantha_var.dunniana:29.611113,Illigera_orbiculata:29.611113)Illigera:29.611113)hernandiaceae:29.611111):29.611115):29.611107):29.611115,((Chimonanthus_salicifolius:59.222225,Chimonanthus_nitens:59.222225,Chimonanthus_grammatus:59.222225,Chimonanthus_campanulatus:59.222225,Chimonanthus_zhejiangensis:59.222225)Chimonanthus:59.222225)calycanthaceae:59.222221)laurales:29.611115):29.611099,(((((Piper_laetispicum:39.481480,Piper_stipitiforme:39.481480,Piper_mullesua:39.481480,Piper_kadsura:39.481480,Piper_hainanense:39.481480,Piper_austrosinense:39.481480,Piper_pedicellatum:39.481480,Piper_lingshuiense:39.481480,Piper_pleiocarpum:39.481480,Piper_senporeiense:39.481480,Piper_polysyphorum:39.481480,Piper_chinense:39.481480,Piper_boehmeriaefolium:39.481480)Piper:39.481480)piperaceae:39.481483):39.481476)piperales:39.481491):39.481476)magnoliids:29.611115,(((Chloranthus_spicatus:66.625000,Chloranthus_elatior:66.625000)Chloranthus:66.625000,(Sarcandra_glabra:66.625000,Sarcandra_hainanensis:66.625000)Sarcandra:66.625000)chloranthaceae:66.625000)chloranthales:66.625000):29.611115)magnoliales_to_asterales:7.222229,((((Kadsura_coccinea:60.666668,Kadsura_oblongifolia:60.666668,Kadsura_induta:60.666668,Kadsura_longipedunculata:60.666668,Kadsura_renchangiana:60.666668,Kadsura_japonica:60.666668,Kadsura_gngustifolia:60.666668,Kadsura_heteroclita:60.666668)Kadsura:60.666668,(Illicium_verum:60.666668,Illicium_philippinense:60.666668,Illicium_majus:60.666668,Illicium_macranthum:60.666668,Illicium_angustisepalum:60.666668,Illicium_difengpi:60.666668,Illicium_modestum:60.666668,Illicium_merrillianum:60.666668,Illicium_pachyphyllum:60.666668,Illicium_brevistylum:60.666668,Illicium_wardii:60.666668,Illicium_lanceolatum:60.666668,Illicium_dunnianum:60.666668,Illicium_henryi:60.666668,Illicium_ternstroemioides:60.666668,Illicium_jiadifengpi:60.666668,Illicium_tashiroi:60.666668,Illicium_leiophyllum:60.666668,Illicium_petelotii:60.666668,Illicium_oligandrum:60.666668,Illicium_arborescens:60.666668,Illicium_tsaii:60.666668,Illicium_griffithii:60.666668,Illicium_micranthum:60.666668,Illicium_simonsii:60.666668,Illicium_tsangii:60.666668,Illicium_burmanicum:60.666668)Illicium:60.666668,(Schisandra_plena:60.666668)Schisandra:60.666668)schisandraceae:60.666664):60.666672)austrobaileyales:60.666672)austrobaileyales_to_asterales:7.222198)nymphaeales_to_asterales:7.222229)angiosperms:7.222222)seedplants:75.000000)euphyllophyte:1.000000;
